# FGFR4 phosphorylates MST1 to confer breast cancer cells resistance to MST1/2-dependent apoptosis

**DOI:** 10.1038/s41418-019-0321-x

**Published:** 2019-03-22

**Authors:** S. Pauliina Turunen, Pernilla von Nandelstadh, Tiina Öhman, Erika Gucciardo, Brinton Seashore-Ludlow, Beatriz Martins, Ville Rantanen, Huini Li, Katrin Höpfner, Päivi Östling, Markku Varjosalo, Kaisa Lehti

**Affiliations:** 1 Department of Microbiology, Tumor and Cell Biology (MTC), Karolinska Institutet, Stockholm, SE-171 77 Sweden; 2 Research Programs Unit, Genome-Scale Biology, Medicum, University of Helsinki and Helsinki University Hospital, Helsinki, FI-00014 Finland; 3 Institute of Biotechnology, Helsinki Institute of Life Science, University of Helsinki, Helsinki, FI-00014 Finland; 4 Department of Oncology and Pathology, Science for Life Laboratory, Karolinska Institutet, Stockholm, SE-171 77 Sweden

**Keywords:** Oncogenes, Kinases

## Abstract

Cancer cells balance with the equilibrium of cell death and growth to expand and metastasize. The activity of mammalian sterile20-like kinases (MST1/2) has been linked to apoptosis and tumor suppression via YAP/Hippo pathway-independent and -dependent mechanisms. Using a kinase substrate screen, we identified here MST1 and MST2 among the top substrates for fibroblast growth factor receptor 4 (FGFR4). In COS-1 cells, MST1 was phosphorylated at Y433 residue in an FGFR4 kinase activity-dependent manner, as assessed by mass spectrometry. Blockade of this phosphorylation by Y433F mutation induced MST1 activation, as indicated by increased threonine phosphorylation of MST1/2, and the downstream substrate MOB1, in FGFR4-overexpressing T47D and MDA-MB-231 breast cancer cells. Importantly, the specific knockdown or short-term inhibition of FGFR4 in endogenous models of human HER2^+^ breast cancer cells likewise led to increased MST1/2 activation, in conjunction with enhanced MST1 nuclear localization and generation of N-terminal cleaved and autophosphorylated MST1. Unexpectedly, MST2 was also essential for this MST1/N activation and coincident apoptosis induction, although these two kinases, as well as YAP, were differentially regulated in the breast cancer models analyzed. Moreover, pharmacological FGFR4 inhibition specifically sensitized the HER2^+^ MDA-MB-453 breast cancer cells, not only to HER2/EGFR and AKT/mTOR inhibitors, but also to clinically relevant apoptosis modulators. In TCGA cohort, FGFR4 overexpression correlated with abysmal HER2^+^ breast carcinoma patient outcome. Therefore, our results uncover a clinically relevant, targetable mechanism of FGFR4 oncogenic activity via suppression of the stress-associated MST1/2-induced apoptosis machinery in tumor cells with prominent HER/ERBB and FGFR4 signaling-driven proliferation.

## Introduction

Cancer cells rely on oncogenic signaling by receptor tyrosine kinases (RTKs) to drive tumor initiation and progression [1]. Upon tumor evolution, RTKs contribute to the development of resistance toward initially effective anticancer treatments. Owing to inhibitable enzyme activity and cell surface localization, RTKs thus serve as attractive therapy targets. Among RTKs, fibroblast growth factor receptors (FGFRs) trigger intracellular signaling cascades that control key cellular processes including survival, proliferation, differentiation, and migration/invasion, as well as angiogenesis—each dysregulated in cancer [2]. Four homologous FGFRs are expressed in humans [2]. Out of these, FGFR4 is dispensable for mouse development [3]. This coupled with specific FGFR4 induction in certain cancers, along with structural differences and drug selectivity relative to other FGFRs, supports the efficacy of FGFR4 as a therapeutic intervention [4–9].

Despite advances in diagnosis and treatment, subsets of breast cancer remain challenging to cure accounting for an estimated 15% of cancer deaths in women [10]. As in other cancers, the poor prognosis is partially attributed to therapy resistance and anti-apoptosis responses of the cancer cells [11]. FGFR4 overexpression and gene alterations, including G388R single-nucleotide polymorphism, have been associated with cancer invasion, drug resistance, and poor prognosis [12–14]. In breast cancers, FGFR4 is overexpressed in especially ERBB2/HER2-enriched tumors [4], where it has been linked to tumor growth and apoptosis resistance [4, 5, 15]. Acquired alterations have been reported in FGFR4 and ERBB2/HER2 upon metastatic cancer progression [16]. Despite these results, the molecular mechanisms explaining how FGFR4 confers aggressive cancer cell behavior remain incompletely understood.

Here, we screened for FGFR4 substrates using in vitro kinase substrate microarrays. Unexpectedly, Hippo tumor suppressor pathway components, including the serine/threonine kinases MST1/2 (mammalian sterile20-like kinases), were among the top tyrosine-phosphorylated substrates. Cytoplasmic MST1/2 comprises the core mammalian Hippo pathway kinase complex, which activation ultimately leads to serine phosphorylation-dependent cytoplasmic retention and inactivation of the oncogenic transcriptional regulators, YES-associated protein (YAP1), and transcriptional co-activator with PDZ-binding motif (TAZ) [17, 18]. Upon cell stress and apoptosis, caspase-3 cleavage removes the inhibitory C-terminal domains of autophosphorylated MST1/2 to induce transport of activated N-terminal MST1/2 into the nucleus [19]. Although overexpression results have shown that nuclear MST1/2 can promote apoptosis [20–23], and reduced MST1/2 activity associates with poor cancer prognosis [24–27], the functional contributions of endogenous MST1/2 in physiological or pathological apoptosis remain elusive [19]. Our screening results led us to investigate in more detail the functional interaction of FGFR4 and MST1/2. Altogether, our results establish a unique oncogenic signaling mechanism—the dominant FGFR4-mediated attenuation of MST1/2-mediated, stress-associated apoptosis in HER2^+^ breast cancer cells.

## Materials and methods

### Kinase substrate identification array

For in vitro FGFR4 substrate identification, the protein microarray containing 9483 human recombinant proteins (Protoarray human protein microarrays version 5.0; Invitrogen, Carlsbad, CA, USA) was blocked and incubated with the recombinant kinase domain of FGFR4 (50 nm; Invitrogen) in the presence of [γ-33P] ATP. The array was washed to remove unbound γ-33P, and exposed to X-ray film. The acquired array image was analyzed using ProtoArray Prospector software (Invitrogen). The raw data were subjected to background subtraction, signal scatter compensation, and outlier detection. The Z-factor cutoff value was set at ≥ 0.4. Phosphorylated proteins with a Z-score > 0.25 were considered as potential substrates.

### Cell culture

Human ZR-75.1, MCF7, BT474, T47D, MDA-MB-453, Hs578T, BT549, MDA-MB-231 (American Type Culture Collection; ATCC, Manassas, VA, USA) and SUM159 [28] breast carcinoma cells, and COS-1 cells (ATCC) were cultured according to manufacturer’s instructions. The MycoAlertPlus kit (LT07-705, Lonza, Basel, Switzerland) was used for confirming the cell cultures negative for mycoplasma.

### Antibodies, inhibitors, and growth factors

The antibodies used were as follows: mouse monoclonal antibodies against ERBB2/HER2 (MA5-13105, Thermo Fisher Scientific (Waltham, MA, USA) for IF, and NCL-L-CB11; Leica Biosystems, Wetzlar, Germany), FGFR4 (sc-136988, Santa Cruz Biotechnology, Dallas, TX, USA), FLAG (9A3; 8146, Cell Signaling Technology, Beverly, MA, USA), glyceraldehyde-3-phosphate dehydrogenase (G8795; Merck, Darmstadt, Germany), MST1 (sc-100449, Santa Cruz Biotechnology, for IF), phospho-tyrosine (05-321, Merck), YAP/TAZ (63.7; sc-101199, Santa Cruz Biotechnology), rabbit monoclonal antibodies against active YAP (EPR19812; ab205270, Abcam, Cambridge, UK), and MOB1 (13730 S), MST1 (D8B9Q; 14946), phospho-MST1/2 (T183/180) (E7U1D; 49332), phospho-MOB1 (pT35) (D2F10; 8699), and phospho-tyrosine (8954, MultiMab) all from Cell Signaling Technology. Rabbit polyclonal antibodies against FGFR4 (sc-124; Santa Cruz), phospho-FGFR4 (pY642; CSB-PA008250, Cusabio Technology, Houston, TX, USA) and phospho-FRS2-α (pY196; 3864), MST1 (3682), MST2 (3952), phospho-p44/42 MAPK (phospho-Erk1/2) (pT202/pY204; 9101), phospho-AKT (pS473; 9271), phospho-MST1/2 (pT183/pT180; 3681), phospho-YAP (pS127; 4911) all from Cell Signaling Technology, V5-tag (ab9116; Abcam), and horseradish peroxidase–conjugated secondary antibodies (P044701 and P044801, Dako, Santa Clara, CA, USA) for enhanced chemiluminescence detection of immunoblots. Species-matched Alexa Fluor conjugated secondary antibodies and phalloidin (Life Technologies, Carlsbad, CA, USA) were used for immunofluorescence. FGFR4 inhibitor BLU9931 (S7819) [29] was purchased from Selleckchem (Munich, Germany), and MST1/2 inhibitor XMU-MP-1 (6482) [30] from Tocris Bioscience (Bristol, UK). Okadaic acid (10011490) was purchased from Cayman Chemical (Ann Arbor, MI, USA). Recombinant human fibroblast growth factor 1 (GF002) and fibroblast growth factor 2 (GF003) were purchased from Merck.

### cDNA constructs, small interfering RNAs, and short hairpin RNAs

Flag epitope (N-terminal)-tagged MST1 and MST2 in the p3FLAG-CMV-10wt (Merck) expression vectors have been described previously [31]. MST1-Y433F mutant was generated by site-directed mutagenesis. FGFR4 and its kinase activity-deficient mutant (K503M) were cloned into pcDNA3.1/V5-His vector, and the expression vector for FGFR4 G388 variant was generated from R388 variant by site-directed mutagenesis [32, 33]. Pools of four siRNAs against human STK3 (MST2; L-004874), STK4 (MST1; L-004157), FGFR4 (L-003134), and nontargeting control siRNA (D-001206-14) (all from Dharmacon/Horizon Discovery, Cambridge, UK) were used; and for FGFR4 also SI02665306 (siFGFR4_6), SI00031360 (siFGFR4_2) and SI00031374 (siFGFR4_4) from Qiagen (Hilden, Germany).

Short hairpin RNA (shRNA) targeted against FGFR4 (TRCN0000000628 and TRCN0000199510; or nontargeting scrambled shRNA were used (Dharmacon/Horizon Discovery). The packaging plasmid (pCMVdr8.74), envelope plasmid (pMD2-VSVG), and FGFR4 or scrambled shRNA in pLKO.1 vector were co-transfected into 293FT producer cells using Lipofectamine 2000 reagent (Invitrogen). Complete breast carcinoma cell growth medium was changed on 293FT cells 24 h after transfection. The viral supernatants were collected after 48 h, passed through a 0.45-µm filter, and incubated with human breast carcinoma cells. After 16 h of infection, the supernatants were replaced with complete media followed by puromycin (2–5 µg/ml) selection of the transduced cells [34].

### Cell transfections, sphere preparation, and treatments

The cells were transfected with expression vectors using FuGENE HD (Promega, Madison, WI) and siRNAs using Lipofectamine 2000 (Invitrogen), Interferin (Polyplus-transfection SA, Illkirch-Graffenstaden, France) or Dharmafect 1 or Dharmafect 2 (Dharmacon/Horizon Discovery). For 2D immunofluorescence, cells were seeded on monomeric collagen type I-coated (50 μg/ml, C7661, Merck) coverslips, fixed in 4% paraformaldehyde (pH 7.5), and stained as previously described [35], and mounted in Vectashield with (DAPI; Vector Laboratories, Burlingame, CA, USA).

Spheres of 24,000 cells were allowed to form under non-adherent conditions in agarose-coated 96-well plates in cell culture media supplemented with 0.2 mg/ml Matrigel (growth factor reduced, 354230; Corning/Merck), or on ultra-low attachment 96-well plate (Corning® Costar® CLS7007, Merck) without Matrigel, and cultured for 2–8 days [36]. The spheres were cultured in the indicated serum concentrations, or serum-starved for 16 h before inhibitor treatment, followed by lysis with radioimmunoprecipitation assay (RIPA) buffer. For immunofluorescence, the spheres were fixed in 4% paraformaldehyde (pH 7.5), post-fixed in ice-cold acetone-methanol (1:1) solution, incubated in blocking buffer (5% bovine serum albumin (BSA), 0.1% Triton X-100 in PBS) and stained for MST1 and YAP. Nuclei were visualized with Hoechst 33342 stain (Thermo Fisher Scientific). The spheres were mounted on glass slides in Vectashield (Vector Laboratories). At least two independent cultures per stable lentiviral shRNA transduction were analyzed for MST1.

### Immunoprecipitation and immunoblotting

Immunoprecipitation and immunoblotting were performed as described previously [37]. Cells were lysed with RIPA buffer (50 mm Tris-HCl pH 7.4, 150 mm NaCl, 1% Igepal CA-630, 0.5% sodiumdeoxycholate) containing Complete protease inhibitor cocktail (Merck), 2 mm Na_3_VO_4_, and 2 mm NaF and cleared by centifugation. For immunoprecipitation from the soluble cell lysates, the samples were further diluted 1:3 with Triton lysis buffer (50 mm Tris-HCl pH 8.0, 150 mm NaCl, 1% Triton X-100, 5 mm CaCl_2_, 0.02% NaN_3_) containing Complete protease inhibitor cocktail, 2 mm Na_3_VO_4_, and 2 mm NaF and pre-cleared with protein A Sepharose. MST1 or MST2 were immunoprecipitated from cleared supernatants with EZview Red anti-FLAG M2 Affinity Gel (Merck) for 4 h at 4 °C. After washing with Triton lysis buffer, bound proteins were eluted with reducing sodium dodecyl sulphate (SDS-PAGE) sample buffer (0.12 m Tris-HCl pH 6.8, 0.02% bromophenol blue, 4% SDS, 50% glycerol, 0.1 m dithiotreitol). Protein concentrations were determined with BCA Protein Assay Kit (23225, Thermo Fischer Scientific), and equal protein amounts (µg) were separated by SDS-PAGE and transferred to nitroclellulose membranes for subsequent detection with primary antibodies and matched secondary antibodies conjugated to horseradish peroxidase. All immunoblots are representative images of experiments repeated independently at least three times; except for Figs. 5b, e and 6a, b, where the experiments were repeated twice.

### Mass spectrometry analysis of phosphorylation sites

All buffers of immunoprecipitation and elution procedures were supplemented with Complete protease inhibitor cocktail and PhosSTOP phosphatase inhibitor tablets (Merck). After the general immunoprecipitation washes, the anti-FLAG affinity gel was washed with pre-urea wash buffer (50 mm Tris pH 8.5, 1 mm ethylene glycol-bis(β-aminoethyl ether (EGTA), 75 mm KCl) before elution with urea buffer (6 m urea, 20 mm Tris pH 7.5, 100 mm sodium chloride). Elution cycles were repeated three times for 30 min each at room temperature with agitation. Proteins in eluates were reduced with (tris(2-carboxyethyl)phosphine), alkylated with iodoacetamide, and trypsin digested with Sequencing Grade Modified Trypsin (Promega). Phosphopeptide enrichment was performed using immobilized metal ion affinity chromatography with titanium (IV) ion (Ti^4+^-IMAC). IMAC material was prepared and used essentially as described [38]. The liquid chromatography with tandem mass spectrometry (LC-MS/MS) analysis was performed using a Q Exactive ESI-quadrupole-orbitrap mass spectrometer coupled to an EASY-nLC 1000 nanoflow LC (Thermo Fisher Scientific), using Xcalibur version 3.1.66.10 (Thermo Fisher Scientific). The phoshopeptide sample was loaded from an autosampler into a C18-packed precolumn (Acclaim PepMap™100; 100 μm×2 cm, 3 μm, 100 Å, Thermo Fisher Scientific) in buffer A (1 % acetonitrile, 0.1 %, formic acid). Peptides were transferred onward to a C18-packed analytical column (Acclaim PepMap™100 75 μm×15 cm, 2 μm, 100 Å, Thermo Fisher Scientific) and separated with 60-minute linear gradient from 5 to 35 % of buffer B (98 % acetonitrile, 0.1% formic acid) at the flow rate of 300 nl/minute. The MS analysis was performed in data-dependent acquisition in positive ion mode. MS spectra were acquired from m/z 300 to m/z 2000 with a resolution of 70,000 with Full AGC target value of 3,000,000 ions, and a maximal injection time of 120 ms, in profile mode. The ten most abundant ions with charge states from 2 + to 7 + were selected for subsequent fragmentation (higher energy collisional dissociation, HCD) and MS/MS spectra were acquired with a resolution of 17,500 with AGC target value of 5000, a maximal injection time of 120 ms, and the lowest mass fixed at m/z 120, in centroid mode. Dynamic exclusion duration was 30 s. Raw data files were analyzed with the Proteome Discoverer software version 1.3 (Thermo Fisher Scientific) connected a Sequest search engine version 28.0 (Thermo Fisher Scientific) against the human component of the Uniprot Database (version 07_2017). Carbamidomethylation (+57.021 Da) of cysteine residues was used as static modification. Phosphorylation of Ser/Thr/Tyr (+79.966 Da) and oxidation (+15.994 Da) of methionine was used as dynamic modification. Precursor mass tolerance and fragment mass tolerance were set to < 15 ppm and 0.05 Da, respectively. A maximum of two missed cleavages was allowed. The software phosphoRS [39] was used to calculate the indvidual site probabilities for phosphorylated peptides.

### Flow cytometry analysis of apoptosis

The proportions of apoptotic cells were determined by flow cytometry for annexin V and propidium iodide (PI) binding [40]. Stable shScr or shFGFR4 transduced MDA-MB-453 cells were transfected with siRNAs. After 72 h the cells, including floating cells in the medium, were collected by trypsinization, washed and labeled with annexin V-Alexa Fluor488 (A13201; Life Technologies) diluted into binding buffer (10 mm HEPES—140 mm sodium chloride—2.5 mm calcium chloride) for 15 min at room temperature. Cells were suspended into binding buffer, stained with 1 µg/ml PI (P3566, Life Technologies), and analyzed with BD Accuri C6 flow cytometer (BD Biosciences, San Jose, CA, USA). Gating and data analysis were performed using FlowJo v10.1 software (Tree Star Inc., Ashland, OR, USA). Geometric mean fluorescence of annexin V binding (FL1-A) alone or combined with PI binding (FL3-A) was quantified. Experiments were repeated three times.

### In vitro kinase assays

MST1 Kinase Enzyme System, including Axltide peptide (KKSRGDYMTMQIG) as MST1 substrate, with ADP-Glo reagent (V4153, Promega) was used according to manufacturer’s instructions to measure ADP production in kinase reactions. The reaction buffer (40 mm Tris, pH 7.5—20 mm magnesium chloride—0.1 mg/ml BSA—50 μm dithiotreitol) was supplemented with 2.5 µm manganese chloride to enhance tyrosine kinase activity. Recombinant MST1 kinase (3 ng) was incubated with 0–10 ng of recombinant kinase domain of FGFR4 (P3054, Thermo Fisher Scientific), together with 1 µm XMU-MP-1 (MST1/2 inhibitor) as an assay control. The kinase reactions with final 50 μm ATP concentration were incubated at room temperature for 1 h on ProxiPlate (PerkinElmer, Waltham, MA, USA), ADP-Glo reagent was added for 40 min, followed by addition of kinase detection reagent for 30 min’ incubation before reading the luminescence with EnSight plate reader (PerkinElmer). Experiment was repeated three times. For in vitro kinase assay detection by immunoblotting, 40 ng of MST1 kinase and 0–100 ng recombinant kinase domain of FGFR4 were incubated together for 30 min at 30 °C, with or without 1 µm XMU-MP-1 or 100 nm BLU9931 (FGFR4 inhibitor) using the abovementioned buffers and MST1 substrate. Proteins in the kinase reaction were denatured and reduced using SDS-PAGE sample buffer (0.12 m Tris-HCl pH 6.8, 0.02% bromophenol blue, 4% SDS, 50% glycerol) supplemented with 10% (v/v) β-mercaptoethanol, and subjected to immunoblotting for detecting phosphorylation of MST1 and FGFR4. Experiment was repeated two times.

### Drug sensitivity and resistance testing and data processing

Compounds (from FIMM Oncology set; https://www.fimm.fi/en/services/technology-centre/htb/equipment-and-libraries/chemical-libraries) and viability controls (DMSO, 100 µm benzethonium chloride) were predispensed on tissue culture treated 384-well plates (Corning, NY, USA). Each compound was plated as singlicate in five concentrations spanning a 10,000-fold concentration range (10-fold dilution). Assay ready plates were stored in pressurized StoragePods (Roylan Developments, Surrey, UK) under inert atmosphere until used. Using a MultiDrop Combi (Thermo Scientific) 5 µL media with or without 500 nm BLU9931 (5× concentration) was first dispensed into assay ready plates and centrifuged briefly. Twenty microliters of a single-cell suspension (2000 cells) was then seeded using MultiDrop Combi peristaltic pump to the plates, which were centrifuged briefly and transferred to an incubator (37 °C and 5% CO_2_). As a surrogate for cell viability, cellular ATP levels were assessed 72 h after plating using CellTiterGlo 2.0 (Promega) with detection on an EnSight plate reader (PerkinElmer). Using in house software, data from the plate reader were normalized per plate to percent viability using values from control wells. Concentration-response curves were fitted to percent viability values using a four-parameter logistic model, and processed further to a sensitivity metric (drug sensitivity score; DSS) using a weighted area-under-the-curve calculation [41]. DSS for each drug was compared between conditions tested.

### Real-time quantitative PCR (qPCR)

RNA was extracted with an RNeasy Plus Mini kit (Qiagen), coupled with RNeasy MinElute columns (Qiagen) for small amount of starting material from non-adherent sphere cultures, followed by reverse transcription with Maxima First Strand cDNA Synthesis Kit (Thermo Scientific). mRNA expression was quantified using TaqMan Fast Advanced Master Mix (Applied Biosystems, Foster City, CA, USA), and validated primers (connective tissue growth factor (CTGF), Hs01026927_g1; cysteine rich angiogenic inducer 61 (CYR61), Hs_00998500_g1; ankyrin repeat domain 1 (ANKRD1), Hs_00173317_m1; TATA-binding protein (TBP) Hs99999910_m1; Applied Biosystems). The expression was normalized with TBP mRNA expression. Two independent RNA extractions were performed, each analyzed in triplicates by qPCR.

### Fibrin-embedded 3D cell cultures for immunohistochemisrty

Preparation of cross-linked fibrin gels was perfomed essentially as described [42], and 17,250 cells were suspended into 75 µl of fibrin gel and casted to 96-well plate. Matrix-embedded single cells were cultured with complete growth medium atop for 7–8 days before first treatment with 100 nm BLU9931 and/or 30 ng/ml FGF1. Inhibitor treatment was added again 72 h later, and cells were fixed after 13–14 days of culture with 4% paraformaldehyde in PBS for 4 h at room temperature. Fibrin gels were dehydrated, paraffin-embedded for sectioning, and subjected immunohistochemistry essentially as described [43]. Sections were subjected to heat-induced antigen retrieval in Tris-ethylenediaminetetraacetic acid, pH 9.0 (for Ki67 antibody, ACK02, Leica Biosystems), or in sodium citrate, pH 6.0 (for Bax antibody, HPA027878, Sigma-Aldrich). Sections were subsequently incubated in 3% (v/v) hydrogen peroxide for 10 min. For antigen detection, tyramide signal amplification (TSA™, PerkinElmer) technology, followed by incubation with 3‐amino‐9‐ethyl‐carbazole solution, were used according to manufacturer’s instructions. Total 30 colonies from two replicate samples were quantified for positively stained vs. total number of cells.

### Imaging and image quantification

Fluorescence images were obtained using an AxioImager.Z2 upright epifluorescence microscope with Plan-Apochromat × 20/0.8 NA dry or × 40/1.4 NA oil objective. In addition, an LSM 780 confocal microscope with Plan-Neofluar × 40/1.3 NA oil objective was used (all from Carl Zeiss, Oberkochen, Germany). Brightness and contrast were linearly adjusted using ZEN 2012 (blue edition; Carl Zeiss). Single optical sections or a combination of two serial optical sections were used for image display.

The MST1 and YAP-stained sphere images were analyzed using Anima [44]. Cell nuclei were detected from 4′,6-diamidino-2-phenylindole (DAPI) channel by first normalizing images with adaptive histogram equalization, and then using the Shape filtering segmentation method found in Anima. The MST1 or YAP signal intensity values were measured from the nucleus area and from a ring around the nucleus representing the cytoplasm. The ring width was determined as 50% of the radius of the nucleus. The nucleus/cytoplasm intensity ratio was calculated by dividing nuclear intensity with cytoplasmic intensity for each nucleus, and then calculating a median value for each image.

Image quantifications of western blotting were performed by processing all obtained micrographs with ImageJ software.

### Statistics

All numerical values represent mean ± SD or SEM as indicated in figure legends. Statistical significance was determined using two-tailed Student's *t* tests for analysis of apoptosis, quantifications of immunoblots and immunohistochemistry. The Kolmogorov–Smirnov test was used for computerized image analysis data on MST1 and YAP-stained sphere images.

## Results

### FGFR4 tyrosine phosphorylates Hippo pathway proteins in vitro and in cells

**Fig. 1:**
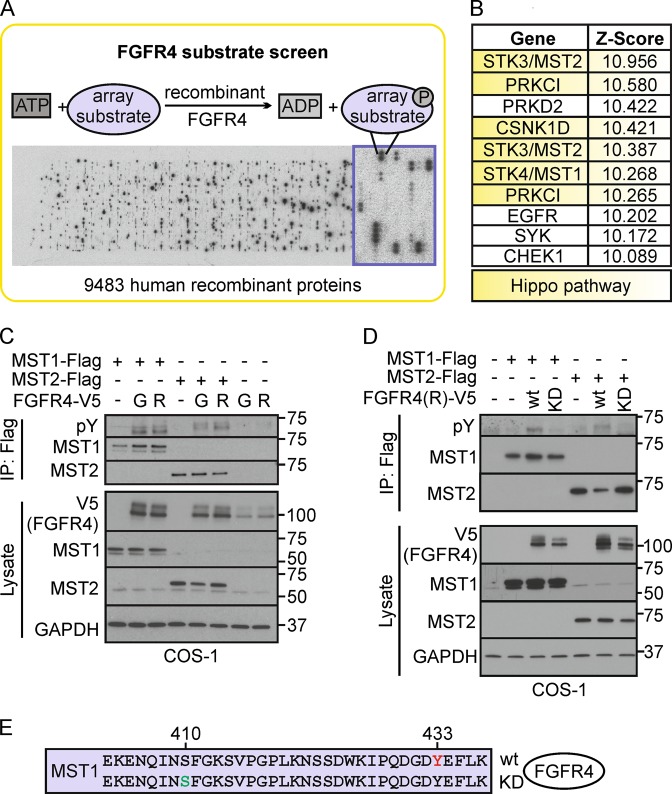
FGFR4 substrate screen identifies tyrosine-phosphorylated Hippo pathway proteins including MST1/2. **a** Scheme of the substrate screen with recombinant FGFR4 kinase domain. **b** Top 10 FGFR4 substrates ranked by the Z-score include Hippo pathway -associated proteins (yellow). See Table S1 for the full substrate list. **c**, **d** MST1/2 are tyrosine phosphorylated by FGFR4 in COS-1 cells. Flag-tagged MST1/2 were immunoprecipitated after transfection of MST1 and MST2 alone or in combination with FGFR4 G388 (G), or R388 (R) kinase (wt), or kinase-dead (KD) variants, and detected by immunoblotting. **e** MST1 immunoprecipitates from COS-1 cells co-transfected with FGFR4 (R)-wt or FGFR4 (R)-KD (See Fig. S1A) were trypsin digested and subjected to phoshopeptide enrichment prior to LC-MS/MS analysis (*N* = 3) that identified phosphorylated Y433 (red) on MST1 only with FGFR4 (R)-wt, and phosphorylated S410 (green) only with FGFR4 (R)-KD

To systematically screen for substrates serving as downstream effectors of FGFR4, we used recombinant FGFR4 kinase domain to assess in vitro phosphorylation of 9483 human recombinant proteins (Fig. 1a, Table S1, Z-score ranking). Unexpectedly, the top five substrates included four Hippo tumor suppressor pathway proteins; MST2 (*STK3*), protein kinase C iota (*PRKCI*), casein kinase I delta (*CSNK1D*) [45], and MST1 (*STK4*) (Fig. 1b; STK3 and PRKCI identified as two splice variants), suggesting that FGFR4 can directly phosphorylate the Hippo serine/threonine kinase pathway proteins (Fig. 1b). To validate this activity, MST1/2 were immunoprecipitated from COS-1 cells after transfection of MST1 and MST2 alone or in combination with FGFR4-G (G388) or the cancer-associated FGFR4-R (R388) variant. Notably, both variants induced MST1 and MST2 tyrosine phosphorylation, revealing these Hippo kinases as novel FGFR4 substrates (Fig. 1c). Importantly, the FGFR4-R-mediated MST1/2 phoshorylation was not detected with kinase activity-deficient FGFR4-R-KD (Fig. 1d).

To identify the FGFR4 phosphorylated tyrosine residue(s), immunoprecipitated MST1 was subjected to mass spectrometry (Fig. S1A). Notably, MST1 from FGFR4 expressing cells was phosphorylated at Y433, whereas MST1 from cells with inactive FGFR4 KD lacked detectable tyrosine phosphorylation (Fig. 1e, Table 1). In addition, an uncharacterized MST1 phosphorylation at S410 near the pY433-site was detected only with kinase-deficient FGFR4 (Fig. 1e, Table 1), whereas MST1 S320 and T177 were phosphorylated in both samples independently of FGFR4 activity (Table 1).

**Table 1:** List of MST1 phoshopeptides identified by mass spectrometry

Co-transfected kinase	Protein	Phospho-residue	Peptide sequence	Peptide FDR	phosphoRS site probabilities (%)
FGFR4/R-wt	MST1_Q13043	Y(433)	IPQDGD**Y**EFLK	<0.01	100
	MST1_Q13043	S(320)	EVDQDDEEN**S**EEDEMDSGTMVR	<0.01	100
	MST1_Q13043	S(410)	EKENQIN**S**FGK	N/A	0
	MST1_Q13043	T(177)	LADFGVAGQLTD**T**MAK	<0.01	99.9
FGFR4/R-KD	MST1_Q13043	Y(433)	IPQDGD**Y**EFLK	N/A	0
	MST1_Q13043	S(320)	EVDQDDEEN**S**EEDEMDSGTMVR	<0.01	100
	MST1_Q13043	S(410)	EKENQIN**S**FGK	<0.01	100
	MST1_Q13043	T(177)	LADFGVAGQLTD**T**MAK	<0.01	100

### FGFR4 is overexpressed in HER2^+^, MST1/2^low^ breast cancer cells, and correlates with adverse outcome in HER2-enriched breast cancer patients

**Fig. 2:**
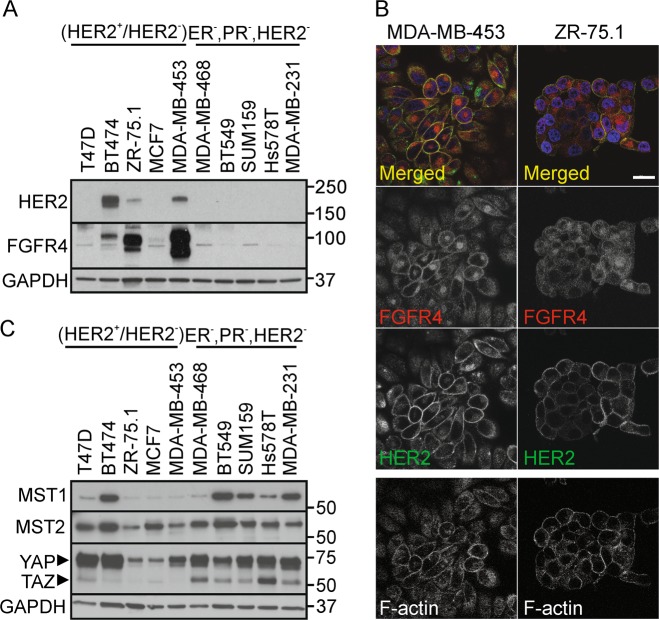
FGFR4 is overexpressed in HER2^+^, MST1/2^low^ breast cancer cells. **a**, **b** FGFR4 and HER2 expression in luminal MDA-MB-453, ZR-75.1, and BT474, MCF7, and T47D, and five triple-negative breast cancer cell lines by **a** immunoblotting and **b** immunofluorescence. Scale bar 20 μm. **c** MST1, MST2, and YAP/TAZ expression in these cell lines, detected by immunoblotting (*N* = 3)

To understand the significance of the identified novel FGFR4 activity, and to establish relevant cell models, we first analyzed FGFR4 expression using TCGA by cBioPortal for Cancer Genomics [46, 47]. In breast cancer, FGFR4 was overexpressed in 33% of HER2-enriched tumors (PAM50 classified, *n* = 58) [48], and significantly associated with poorer overall survival compared with the non-overexpressing group (Fig. S1B; *P* = 0.044; FGFR4 overexpression in 4% of all breast cancers; *n* = 825 [4]). Consistently, MDA-MB-453 (ER^−^, PR^−^, HER2^+^) as well as ZR-75.1 and BT474 (ER^+^, PR^+/−^, HER2^+^) breast cancer cells expressed FGFR4, whereas both FGFR4 and HER2 were low in MCF7 and T47D (ER^+^, PR^+/−^, HER2^−^), and negligible in triple-negative (ER^−^, PR^−^, HER2^−^) cells (Fig. 2a, S1C) [49]. By immunofluorescence, strong intracellular and cell surface FGFR4 was associated with cell surface HER2 in MDA-MB-453, which harbor an activating FGFR4 mutation [50, 51], and in ZR-75.1, rendering these cells suitable endogenous models for FGFR4 function (Fig. 2b).

Among these cell lines, MST1 was lowest in ZR-75.1, MCF7, and MDA-MB-453 (Fig. 2c). Moreover, FGFR4^+^ ZR-75.1 and MDA-MB-453 expressed low levels of MST2 and TAZ, whereas TAZ was prominent in triple-negative, and YAP in all except ZR-75.1 and MCF7 cells (Fig. 2c).

### Endogenous FGFR4 inhibits MST1/2 activation and cleavage

**Fig. 3:**
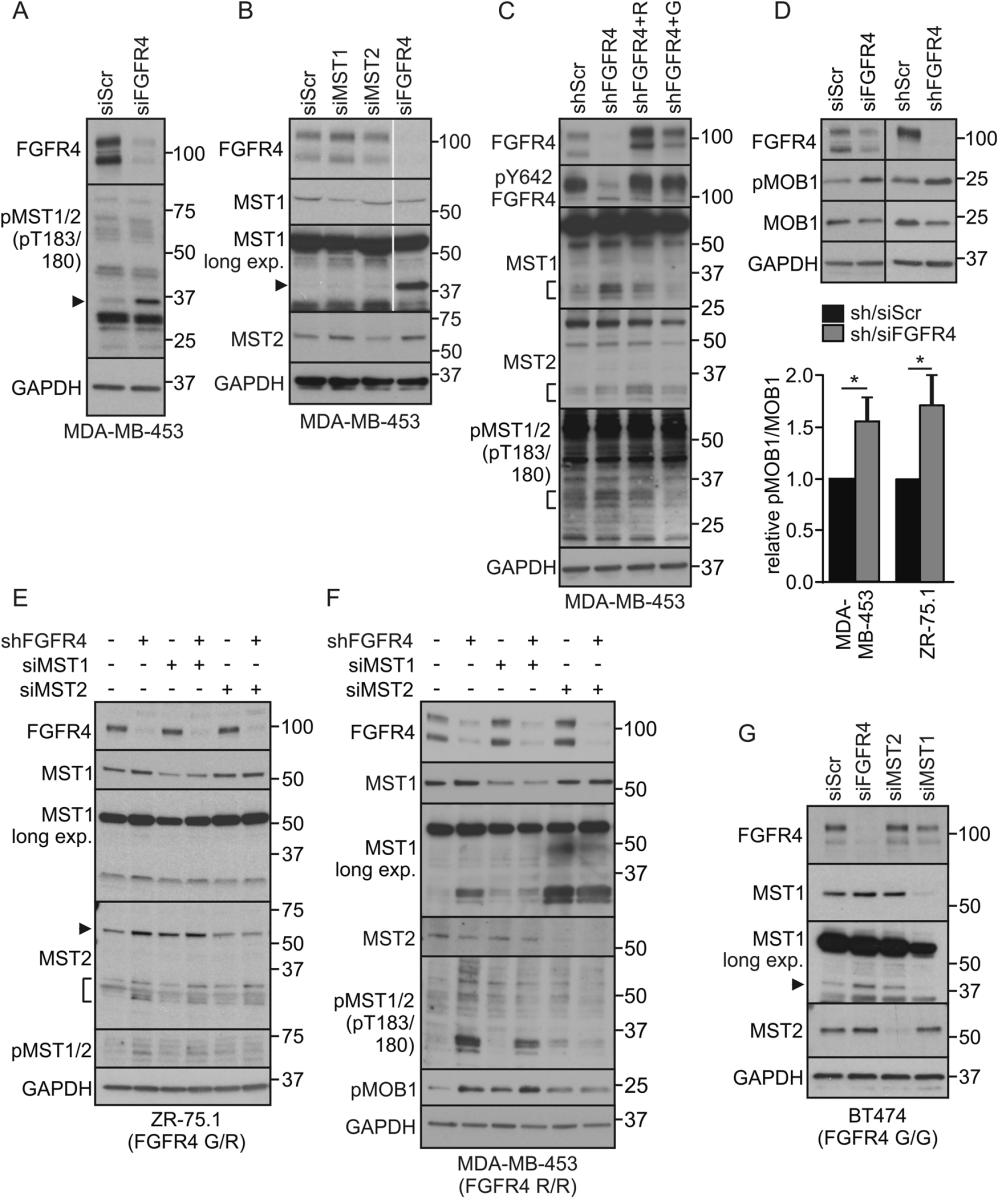
FGFR4 suppresses MST1/2 activation and cleavage in HER2^+^ breast cancer cells. **a**, **b** MDA-MB-453 cells transfected with indicated siRNAs were subjected to immunoblotting for **a** T183/180 phosphorylated MST1/2, and **b** MST1 and MST2. Note cleaved ~ 37 kDa MST1/N in FGFR4 knockdown cells (arrowhead). Thin gray line indicates cropping to leave out irrelevant sample lane; see uncropped immunoblots in Fig. S8. **c** MDA-MB-453 cells transduced with indicated shRNAs were transfected with siScr or siFGFR4 siRNA to 3’UTR before transfection of mock or FGFR4 (R) or (G) overexpression plasmid for a rescue experiment. Lysates were subjected to immunoblotting as indicated. Brackets indicate the cleaved MST1 and MST2 fragments. See Fig. S2A for phopsho-FRS2α and short exposure of MST1. **d** MDA-MB-453 and ZR-75.1 cells were transduced with indicated si/shRNAs; upper, indicated immunoblots of lysates; lower, quantification of pMOB1/MOB1 ratio, *N* = 3, mean ± SEM; *P < 0.05. For MST1/2 knockdown **e** ZR-75.1 and **f** MDA-MB-453 were transduced with shRNAs followed by transfection with siRNAs as indicated, and **g** BT474 cells were transfected with indicated siRNAs, and subjected to immunoblotting for pT183/180 MST1/2, MST1, MST2, and pMOB1 as indicated (in **e** arrowhead points to a full-length, bracket to the cleaved MST2) **a–g**. *N* = 3 independent repeats for all; except *N* = 2 in **f** and **g**

To test if FGFR4 regulates the tumor-suppressive MST1/2 kinases, MDA-MB-453 cells were transfected with siRNAs specific for FGFR4, MST1, and MST2. In control cells, weak protein bands corresponding to full length and cleaved MST1/2 were detected with antibodies against active, pT183/180-autophosphorylated MST1/2 (Fig. 3a). Markedly, FGFR4 knockdown enhanced the cleaved active pMST1/2 (Fig. 3a, arrowhead). Total MST1, present in control cells as full-length protein, became detectable as an additional 36/37 kDa form after FGFR4 knockdown (Fig. 3b; see N-terminal form similar to the caspase-cleaved MST1/N; [19, 23]). MST2 instead was detected as full-length protein (Fig. 3b). Importantly, after lentiviral FGFR4 silencing (shFGFR4), ectopic FGFR4 (R388 and G388 variant) expression rescued the cleaved MST1 and pMST1/2 back to the low levels of controls (shScr) (Fig. 3c, S2A). In contrast, cancer-associated FGFR4-R slightly induced MST2 cleavage (Fig. 3c; FGFR4-G/R vary in stability and signal output; endogenous FGFR4 in MDA-MB-453 contains G388R and the activating Y367C alterations [12, 33, 50–52]). In MDA-MB-231, FGFR4-R, and -G also decreased the cleaved MST1 (Fig. S2B), and even more efficiently upon FGF1 and FGF2 stimulation as shown in FGFR4-R cells (Fig. S2C).

Coincident with increased MST1/2 cleavage and activation, FGFR4 knockdown resulted in significantly increased threonine (T35) phosphorylation of the MST1/2 substrate MOB1 in MDA-MB-453 (Fig. 3d; *P* < 0.05; [53]). In ZR-75.1, FGFR4 silencing likewise enhanced pMOB1 (Fig. 3d; *P* < 0.05) and pMST1/2 (Fig. 3e). However, this was coupled with increased full-length and cleaved MST2, whereas cleaved MST1 was less enhanced (Fig. 3e). To clarify distinct MST contributions to FGFR4 depletion-induced pMST1/2, FGFR4 was silenced alone and in combination with MST1 or MST2 in MDA-MB-453. In subconfluent low-serum cultures with prominent activation of MST1/N upon FGFR4 depletion, partial MST1 knockdown increased pMOB1 with and without FGFR4 (Fig. 3f; note that serum suppresses distinctively MST2 [54]). Instead, MST2 double knockdown with FGFR4 blocked the enhanced threonine phosphorylation of MOB1 and cleaved MST1/2, while even increasing MST1 cleavage (Fig. 3f). Thus, MST1/2 were interregulated, and MST2 was essential for the induction of active MST1/N and pMOB1 after FGFR4 depletion.

As MDA-MB-453 and ZR-75.1 both contain the cancer-associated FGFR4-R388 (MDA-MB-453, homozygous; ZR-75.1, heterozygous), we further silenced FGFR4 in BT474 homozygous for FGFR4 G388, which increased MST1/N as well as full-length MST1/2 (Fig. 3g), indicating that both FGFR4 variants can suppress MST1.

### FGFR4 counteracts MST1/2-mediated apoptosis

**Fig. 4:**
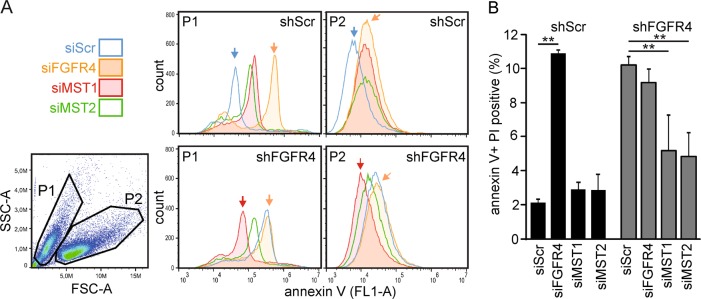
FGFR4 counteracts MST1/2-mediated apoptosis. MDA-MB-453 cells transduced with shScr or shFGFR4 shRNAs were transfected with siRNA pools specific for FGFR4, MST1 or MST2, and analyzed for annexin V and propidium iodide (PI) binding by flow cytometry using two different gating strategies for data visualization. **a** Gating to populations P1 (smaller) and P2 (larger), and annexin V binding (FL1-A) histograms as a marker for early apoptotic cells. **b** Quantification (% of total, 100,000 events) of apoptosis based on double-positive (annexin V + PI) cells, including both early and late apoptotic stages. See Fig. S3B for representative contour plots and quadrant gating. Mean ± SD of triplicates shown, **P < 0.01; (repeated three times; *N* = 3). FSC-A; forward scatter, and SSC-A; side scatter

MST1/2 activation by T183/180 autophosphorylation and cleavage is associated with apoptosis [19]. To investigate if FGFR4 alters the pro-apoptotic MST1/2 function, MDA-MB-453 cells were transduced with shScr and shFGFR4, followed by transfection of FGFR4, MST1, and MST2 siRNAs. Apoptosis was measured by annexin V and PI binding with flow cytometry using two gating strategies: P1 (smaller) and P2 (larger) cells for early apoptosis by annexin V (Fig. 4a), and the whole-cell population for combined early and late apoptotic cells by double positivity (Fig. 4b, S3B). Stable FGFR4 silencing significantly increased apoptosis relative to control (Fig. 4a, b, S3A; *P* ≤ 0.02). Similarly, FGFR4 siRNAs increased apoptosis in shScr cells (Fig. 4a, b; *P* < 0.001). MST1 or MST2 knockdown did not decrease apoptosis in shScr cells with high endogenous FGFR4, and FGFR4 siRNAs did not further increase apoptosis in shFGFR4 cells (Fig. 4a, b). Notably, the apoptosis induction by stable FGFR4 silencing was rescued close to the low level of shScr cell apoptosis by MST1 (*P* = 0.02) or MST2 (*P* = 0.003) knockdown in shFGFR4 cells (Fig. 4a, b, and S3B), suggesting that MST1 and MST2 act together to induce apoptosis, unless attenuated by FGFR4 in MDA-MB-453.

### FGFR4 silencing increases MST1/2 activation and MST1 nuclear translocation in cancer cell spheres

**Fig. 5:**
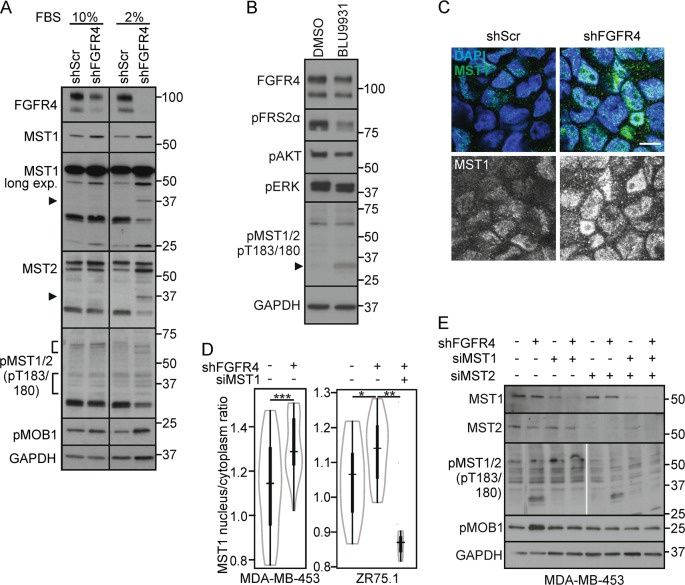
FGFR4 suppresses MST1/2 activation and nuclear localization in cancer cell spheres. **a** shScr and shFGFR4 MDA-MB-453 cell spheres were cultured under non-adherent conditions (10% or 2% FBS), and subjected to immunoblotting. Arrowhead; cleaved N-terminal MST1/2 (in 2% FBS), brackets highlight the fragments of autoactivated MST1/2. **b** MDA-MB-453 cell spheres were treated with 100 nm BLU9931 for 15 min, and subjected to immunoblotting. **c**, **d** shScr and shFGFR4 MDA-MB-453 and ZR-75.1 spheres were analyzed for MST1 expression by **c** immunofluorescence, and **d** MST1 nuclear/cytoplasmic ratio was quantified (*n* = 4–6 MDA-MB-453 spheres, ≥ 6 microscopic fields/sphere; *n* = 2–3 ZR-75.1 spheres, ≥ 8 microscopic fields/ sphere; mean ± SEM of two independent experiments. Scale bar 10 µm. **e** shScr and shFGFR4 MDA-MB-453 cells were transfected with indicated siRNAs before sphere formation, cultured under non-adherent conditions (1% FBS) for 48 h, and subjected to immunoblotting

Three-dimensional (3D) cell spheres recapitulate the cell-cell contacts and dimensionality of in vivo tumors. To investigate MST1/2 regulation in 3D, MDA-MB-453 cells were cultured in non-adherent conditions for sphere formation. In complete medium, control and FGFR4 knockdown spheres had minor differences in MST1/2 and MOB1 total protein and phoshorylation (Fig. 5a; 10% fetal bovine serum; FBS). Markedly, FGFR4 depletion in low serum increased MST1/2 cleavage (total and pT183/180) and pMOB1 (Fig. 5a; 2% FBS). Treatment with FGFR4-specific inhibitor BLU9931 [29] for 15 min likewise increased pMST1/2 (Fig. 5b). During this short FGFR4 inhibition, pFRS2α was diminished, whereas pAKT and pERK remained less affected (Fig. 5b), suggesting that MST1/2 activation upon FGFR4 inhibition is an early event in the apoptosis induction. By immunofluorescence, both MDA-MB-453 and ZR-75.1 spheres had low nuclear and cytoplasmic MST1, whereas FGFR4 silencing specifically increased punctate, nuclear MST1, significantly increasing the nucleus/cytoplasm ratio (Fig. 5c, d, S3C), thus suggesting that FGFR4 inhibits MST1/2 activation and MST1/N nuclear localization to counteract apoptosis.

To further examine the FGFR4-dependent regulation and individual MST1 and MST2 functions, all three genes were silenced in distinct combinations in 3D. As expected, FGFR4 knockdown increased the cleaved pMST1/2 and pMOB1, which response was prevented by MST1 depletion in MDA-MB-453 (Fig. 5e). After MST2 silencing alone or in combinations with FGFR4 and/or MST1, the higher molecular weight pMST1/2 forms were reduced. The cleaved pMST1/2, although reduced, remained detectable after combined MST2 and FGFR4 silencing, suggesting that it corresponds to MST1/N (Fig. 5e). However, combined knockdown of FGFR4 with MST1, MST2 or both the MSTs, prevented pMOB1 induction (Fig. 5e). In ZR-75.1 with MST2-dominated response to FGFR4 knockdown (see Fig. 3e), FGFR4 and/or MST1 knockdown instead enhanced pMOB1, whereas MST2 and FGFR4 double depletion fully prevented this induction (Fig. S4A). Therefore, MST1 and MST2 were cooperatively activated upon FGFR4 depletion, leading to context-dependent dominance of partially competing outcomes of MST2-dependent MST1/N activation or (cytoplasmic) MST2-MOB1 axis.

### Mutation of MST1-pY433-site restores MST1/2 activation in FGFR4 expressing cells

**Fig. 6:**
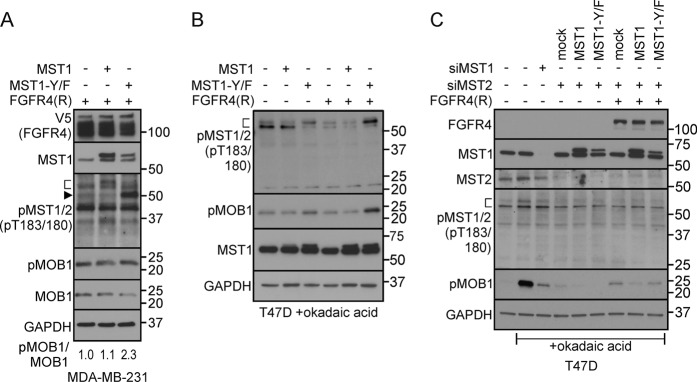
MST1-Y433F phosphosite mutant restores MST1/2 activation in FGFR4 expressing cancer cells. **a** MDA-MB-231 cells co-transfected with FGFR4 (R) and wild-type or phosphosite mutant MST1-Y433F were subjected to immunoblotting as indicated. Ratio of pMOB1/MOB1 is indicated below the immunoblot panel. **b** T47D cells (co-)transfected with wild-type or MST1-Y433F alone or with FGFR4 (R) were treated with 1 µm okadaic acid for 1 h before cell lysis, and subjected to immunoblotting. See corresponding T47D immunoblots without okadaic acid in Fig. S4C. **c** T47D cells with indicated siRNAs, and (co-)transfected with wild-type or MST1-Y433F alone or with FGFR4 (R) were treated with 1 µm okadaic acid as above, and subjected to immunoblotting. **a–c** Brackets and arrowhead indicate the activated pMST1/2 fragments. *N* = 2 independent repeats

To examine the effects of the identified FGFR4-dependent tyrosine phosphorylation in MST1, wild-type MST1 and the pY433-site mutant MST1-Y/F were overexpressed alone and in combination with FGFR4-R in MDA-MB-231 cells. Although weak autoactivated pMST1/2 was detected in cells overexpressing MST1 (wild-type or mutant) alone or MST1 (wild-type) and FGFR4-R, co-expression of MST1-Y433F and FGFR4 resulted in prominent pMST1/2 autophosphorylation (Fig. 6a and Fig. S4B). Coincidentally, the downstream pMOB1 was increased relative to total MOB1 (Fig. 6a). Since inactivating *NF2* mutation in MDA-MB-231 can affect MST1/2 activation [55], we next transfected T47D cells to express FGFR4-R and MST1 (wild-type or Y433F). In these cells, MST1 remained inactive as reflected by unchanged pMST1/2 and pMOB1, except if okadaic acid, known to enhance pMST1/2 by PP2A phosphatase inhibition [56], was added (Fig. S4C). This treatment dramatically increased pMOB1 along with pMST1/2 detection as a doublet in mock and MST1-overexpressing cells (Fig. 6b, S4C). Notably, FGFR4 suppressed pMST1/2 in okadaic acid-treated mock and wild-type MST1 cells, whereas pMST1/2 and pMOB1 were increased after MST1-Y433F and FGFR4 co-expression (Fig. 6b, S4C).

In these okadaic acid-treated cells, MST1 or MST2 knockdown suppressed pMOB1, MST2 depletion being most effective, and decreasing also pMST1/2 doublet (Fig. 6c). Strikingly, MST2 knockdown also blocked the MST1-Y433F-mediated pMST1/2 induction (Fig. 6c). After MST2 depletion, MST1-Y433F even reduced pMOB1, which effect was reverted by FGFR4 (Fig. 6c). Considering such MST2-dependence of FGFR4-mediated MST1 regulation, suggestive of key changes in MST1/2 heterodimer interactions and activity, we tested if FGFR4 can directly alter MST1 activity. The recombinant MST1 activity, measured as ADP generation in vitro, was not altered by recombinant FGFR4, while being inhibited by MST1 inhibitor XMU-MP-1 [30] (Fig. S5A, B). Altogether, this is consistent with mutually competitive MST2 cytoplasmic functions reflected by pMOB1, and pro-apoptotic MST1/2 heterotypic activation, whereby specifically the MST1/2 activation is suppressed by FGFR4-dependent MST1-Y433 phosphorylation.

### Regulation of YAP differs from pro-apoptotic MST1/2 activation

We next analyzed protein alterations and target gene transcription of the canonical Hippo pathway effector YAP, to test if FGFR4 affects cytoplasmic MST1/2 signaling [18, 19]. The nuclear/cytoplasmic YAP ratio remained essentially unaltered in 3D cell spheres (Fig. S6A). However, YAP localization shifted from irregular to more polarized and membrane-proximal pattern after FGFR4 silencing in MDA-MB-453 (Fig. S6B). This coincided with subtle alterations in inactive (pS127-YAP), total, or active (non-pS127) YAP (Fig. S6C, D), without significant changes in mRNAs for the canonical YAP target genes CTGF, CYR61, and ANKRD1 (Fig. S6E). In ZR-75.1, FGFR4 depletion enhanced inactive/total and decreased active YAP in more MST2-dependent manner (Fig. S7A). Therefore, FGFR4 had variable effects on YAP, which did not concur with the pro-apoptotic pMST1/2 regulation.

### Apoptosis evasion by FGFR4 encompasses co-targetable vulnerabilities with mitochondrial apoptosis pathway and HER2/EGFR, AKT, and mTORC1 signaling axes

**Fig. 7:**
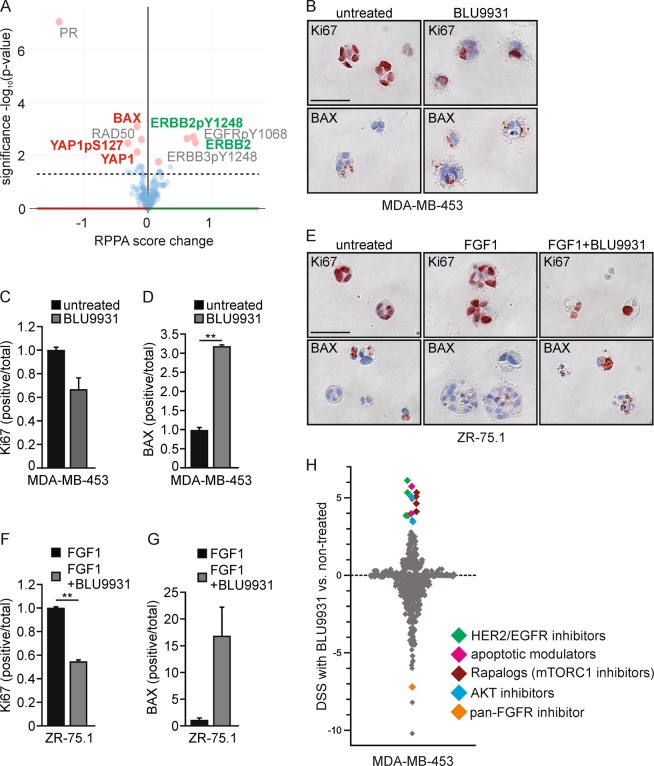
FGFR4 confers resistance to apoptotic modulators in comprehensive drug screen. **a** (Phospho)protein changes in TCGA RPPA data [4] associated with FGFR4 upregulation in breast cancer, visualized using cBioPortal (RPPA score change in breast cancer tumors with and without alterations in FGFR4; (mean FGFR4 altered – mean FGFR4 unaltered) [46, 47]. The most significantly up- and downregulated proteins are highlighted (pink dots); ERBB2, alternative name of HER2; PR, progesterone receptor. **b–g** Fibrin embedded single-cell suspensions of **b–d** MDA-MB-453 and **e–g** ZR-75.1 cells were treated with 100 nm BLU9931 and/or 30 ng/ml FGF1 over a 13–14-day culture, fixed, embedded into paraffin for sectioning, and subjected to immunohistochemistry for Ki67 and BAX expression. Positively stained vs. total number of cells per colony were counted (*N* = 30, mean ± SD, **P < 0.01). Scale bar 50 µm in **b** and **e**. **b** For comprehensive drug sensitivity testing (*N* = 1), MDA-MB-453 cells were treated with 527 compounds in five-point dose either alone or in combination with specific FGFR4 inhibitor BLU9931. Dotplot showing the difference in DSS (drug sensitivity score) for cells in treatment combination with BLU9931 (100 nm) versus single agent treatments. Negative values are compounds inducing larger decreases in viability as single agents; positive scores indicate compounds yielding larger decreases in viability in the presence of BLU9931. Colors demarcate compounds with similar class

To consider the overall impact of FGFR4 in human breast cancer, we systematically analyzed (phospho)protein alterations in FGFR4-overexpressing human tumors using TCGA reverse phase protein array (RPPA) data. Significantly, the pro-apoptotic protein BAX, DNA repair protein RAD50, and YAP (pS127 and total) were downregulated, along with the strongest decrease in progesterone receptor, in FGFR4-overexpressing tumors (Fig. 7a). Moreover, FGFR4 overexpression correlated with increased total and pY1248-HER2/ERBB2, pY1068-EGFR, and pY1248-ERBB3, in conjunction with adverse patient outcome (Fig. 7a, S7B; TCGA [4]).

To relate these alterations in human tumors to the FGFR4-dependent apoptosis evasion, we further analyzed how FGFR4 regulates the cell phenotype in 3D matrix-embedded cultures. The actively growing Ki67-positive FGFR4^+^ MDA-MB-453 cells responded to the specific FGFR4 inhibitor BLU9931 treatment (100 nm) with a tendency to reduced proliferation as assessed by Ki67-positivity (Fig. 7b, c; *P* = 0.07). More strikingly, pro-apoptotic BAX protein positivity coupled to apoptotic membrane blebbing increased significantly (Fig. 7b, d; *P* = 0.001). Untreated ZR-75.1 cells instead grew poorly, but showed prominent Ki67-positive growth upon FGF1 treatment, which was inhibited by BLU9931 (Fig. 7e, f; *P* = 0.01), whereas here the concomitant increase in BAX did not reach significance (Fig. 7e, g).

Considering these indications, further linking FGFR4 to apoptosis evasion in human tumors and 3D cultures, we next systematically interrogated the association of FGFR4 activity with drug sensitivity and resistance, targeting clinically actionable vulnerabilities of cancer cells. To this end, we screened a comprehensive oncology library comprised of 527 approved and investigational drugs in combination with BLU9931 in MDA-MB-453 cells. Consistent with known signaling pathway crosstalk [57–59], these cells were relatively sensitive to several (pan)-PI3K/mTOR and AKT inhibitors, and to a few histone deacetylase (HDAC) inhibitors, as assessed by DSS [36] (Table S2). Moreover, MDA-MB-453 showed distinctive sensitivity towards BET/bromodomain inhibitors, which as with most drugs, was not further affected by BLU9931 at the used concentration (100 nm; Table S2).

Markedly, combination treatment with BLU9931 in comprehensive drug testing sensitized the FGFR4 inhibitor-treated cells to the modulators of mitochondrial apoptosis pathway, BCL-X_L_ inhibitor (A-1155463) and a SMAC mimetic/IAP inhibitor (Birinapant) (Fig. 7h, Table S3). HER2/EGFR, AKT, and mTORC1 inhibitors (rapalogs) likewise showed better efficacy in BLU9931 treated cells, and importantly, compounds with same mechanism of action showed comparable shift in DSSs (Fig. 7h, Table S3). Moreover, MDA-MB-453 cells were sensitive to pan-FGFR inhibitor (LY-2874455), whereas the cells treated with BLU9931 did not respond to additional FGFR targeting over a broad concentration range (Fig. 7h, Table S3).

## Discussion

Apoptosis evasion is one of the classical hallmarks of cancer. Here, we identified the apoptosis-promoting MST1/2 as unique FGFR4 substrates by an unbiased in vitro screen. We provide evidence suggesting that by Y433-MST1 phosphorylation, FGFR4 inhibits MST1/2 activation. In endogenous FGFR4^+^/HER2^+^ breast cancer cell model, this inhibition was essential to counteract apoptosis induction. By this mechanism, FGFR4 can increase cell survival in breast cancers, where fast proliferation is driven by HER/ERBB and FGFR4 signaling [4, 5]. In addition, supporting this conclusion, FGFR4 overexpression was associated with poor HER2^+^ breast cancer patient survival.

MST1 and MST2 are the core kinases of mammalian Hippo tumor suppressor pathway, which complex and context-dependent regulatory mechanisms in health and disease still remain unclear. What is known is that MST1/2 are activated via homo- or heterodimerization followed by trans-phosphorylation at T183/180 [19, 60]. Upon apoptosis, caspase-3-mediated cleavage removes the C-terminal MST1/2 regulatory and nuclear export signals, triggering nuclear translocation of the active N-terminal kinase [20, 21]. Although MST1/2 activity has been implicated in histone phosphorylation and chromatin condensation, functional contribution of endogenous MST1/2 to apoptosis, downstream of caspase-3, remains unclear [19, 21, 61, 62]. Here, we show that in FGFR4^+^/HER2^+^ breast cancer cells, particularly under tumor-mimicking 3D conditions, FGFR4 depletion-induced MST1/2 autophosphorylation coupled with MST1 cleavage and nuclear localization without additional stimuli. This resulted in significant apoptosis induction, which was rescued back to low levels by double knockdown of FGFR4 with MST1 or MST2. In cells with endogenous FGFR4, however, MST1 or MST2 silencing did not reduce apoptosis, suggesting that the cells have developed dependency of the herein identified FGFR4-mediated suppression of apoptosis. Moreover, pharmacological FGFR4 targeting specifically sensitized the cells, in addition to HER2/EGFR and PI3K/AKT/mTOR inhibitors, to the modulators of mitochondrial apoptosis pathway, suggesting interesting possibilities also for combinatorial treatments to effectively halt the FGFR4^+^/HER2^+^ cancers [63].

Previously, other kinases have been indicated in positive and negative MST1/2 regulation [31, 64–67]. In neuronal cells, MST1 phosphorylation by non-receptor tyrosine kinase c-Abl at Y433 (conserved site in mammals, absent in MST2) leads to MST1 stabilization and activation thus increasing cell death [68], an opposite outcome from the herein identified FGFR4-mediated inhibition of MST1/2 and apoptosis. Moreover, c-Abl-mediated phosphorylation of Y81 on MST2 (species-conserved site, absent in MST1) enhances MST2 activation [69]. Our current results indicate that, although involving specific MST1/N cleavage and suppression upon MST1 phosphorylation (pY433), the apoptosis induction by FGFR4 depletion required combined activity of MST1 and MST2, whereas these individual kinases were regulated even in an opposite manner. This can be related to the mechanisms whereby AKT signaling differentially regulates MST1 and MST2 [54, 70], and MST1/2 integrate signals from Ras signaling (Raf-1, RASSF1A) [54, 60, 71, 72]. As recombinant MST1 activity was not inhibited by FGFR4 in vitro, we suggest that FGFR4 likely alters MST1/2 activities via MST1-pY433-regulated interactions. Further context-dependent aspects of MST2 phosphorylation, (hetero)dimerization, signaling scaffolds, and activity or cleavage regulation will remain interesting future study subjects. Moreover, the other herein identified unique MST1-pS410 phosphosite detected only in the absence of FGFR4 activity and pY433, raises the possibility of mutually regulated phosphorylation of these residues. The two MST1 phosphosites detected independently of pY433 have been characterized; S320 as a phosphorylation site for anti-apoptotic CK2 [73], and T177 as a MST1 activity-dependent site [74]. Therefore, the context-dependent net effects of all MST1 phosphosites on its activity regulation, including contributions by kinases other than FGFR4, will be of interest. Nevertheless, current results show that active FGFR4 tyrosine phosphorylates and inactivates the pro-apoptotic MST1/2 serine/threonine kinase in breast cancer cells, thus revealing a novel mechanism of RTK-mediated apoptosis evasion and oncogenic FGFR4 function.

Apart from pro-apoptotic nuclear functions, cytoplasmic MST1/2 act on canonical Hippo signaling, which negatively regulates YAP by serine phosphorylation and cytoplasmic retention [19, 75]. In cancer, however, non-canonical regulation of YAP expression, localization and activity frequently prevails along with the loss of balancing feed-back mechanisms of normal cellular homeostasis [18, 75, 76]. We tested if FGFR4-mediated pro-apoptotic MST1/2 inhibition also regulated YAP. Although YAP (pS127 and total) was decreased in FGFR4-overexpressing breast cancers (TCGA RPPA), the FGFR4-dependent and -independent modulation of MST1/2 had variable effects on YAP S127 phosphorylation or localization. This is consistent with accumulating evidence acknowledging cell polarity, junctional complexes, and cytoskeletal signals as important YAP regulators even independent of MST1/2 [17, 66]. A further interesting aspect is that *FGFR1, -2*, and *-4* are transcriptional targets of YAP, and a feed-forward loop has been described between YAP and FGFR signaling in cholangiocarcinoma and ovarian cancer [77, 78].

In conclusion, the identified oncogenic FGFR4 activity explains mechanistically how RTKs such as FGFR4 can confer aggressiveness to certain cancer cells via apoptosis resistance. As FGFR4 is suitable for targeting, these results raise tempting questions, whether FGFR4 inhibition in HER2^+^ breast cancer would release the intrinsic MST1/2-mediated apoptotic machinery. And if so, would it offer increased efficacy in combination with targeting HER2, AKT/mTOR, and/or mitochondrial apoptosis pathways in this cancer subset, or in other FGFR4-overexpressing cancers? Nonetheless, the identification of MST1/2 as direct substrates for FGFR4, and FGFR4-dependent inhibition of MST1/2-dependent apoptosis, highlights interesting avenues for FGFR4 targeting in anticancer treatments.

## Supplementary information

Supplemental Figures and Legends

Table S1

Table S2

Table S3
