# Supplementary material for: FGFR4 phosphorylates MST1 to confer breast cancer cells resistance to MST1/2-dependent apoptosis
Source: Cell Death Differ. 2019 Mar 22;26(12):2577–93. doi: 10.1038/s41418-019-0321-x (PMC7224384; doi:10.1038/s41418-019-0321-x)
Supplement: Supplementary file 1 — Supplemental Figures and Legends [file 41418_2019_321_MOESM1_ESM.pdf]

Supplemental Figures and legends

Figure S1

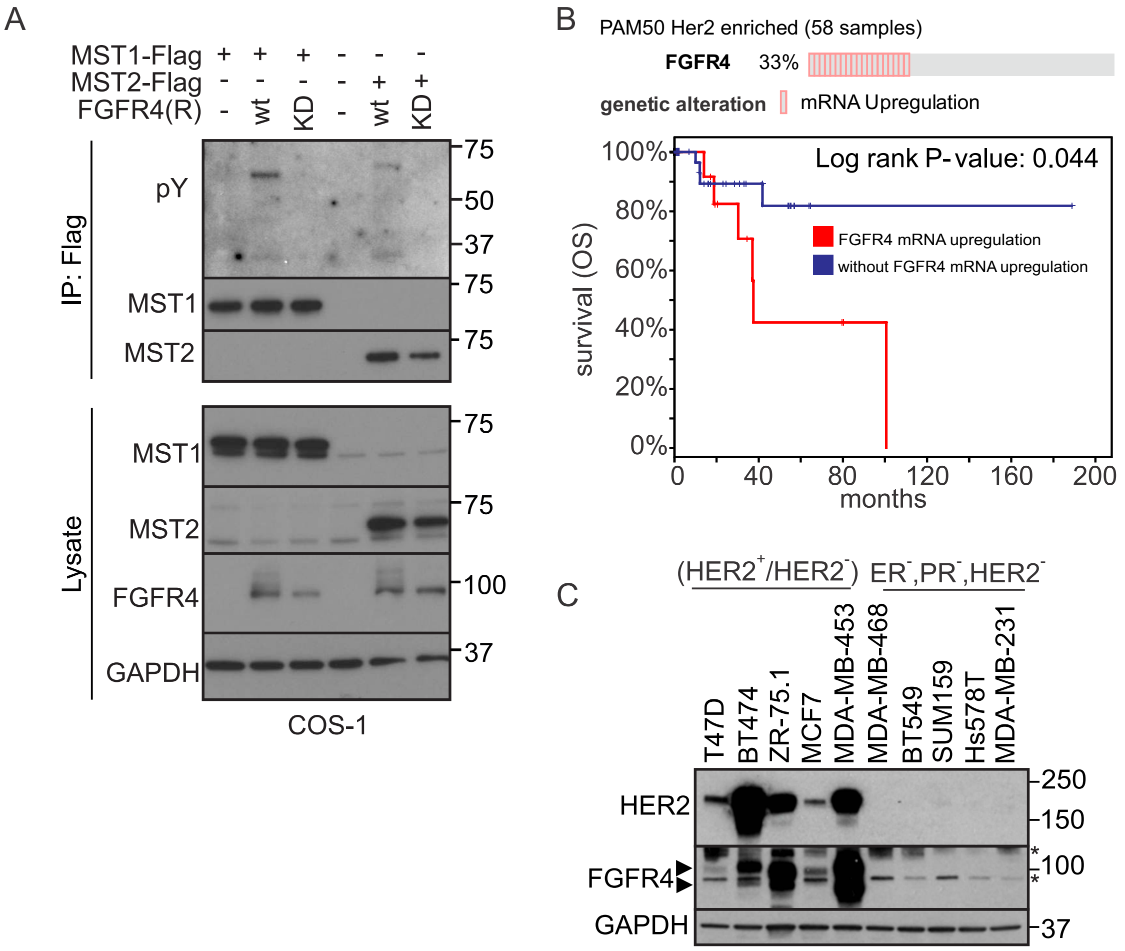

Figure S1. Tyrosine phosphorylation of MST1/2 in COS-1 cells co-transfected with FGFR4. FGFR4 survival association in breast cancer TCGA data and expression in cell lines. A) Flag-tagged MST1/2 were immunoprecipitated from COS-1 cells after transfection of MST1 and MST2 alone or in combination with FGFR4 R388 (R) kinase (wt) or kinase-dead (KD) variants, and detected by immunoblotting**.** For phosphorylation site identification, the urea-eluted MST1 immunoprecipitates from COS-1 cells co-transfected with FGFR4 (R)-wt or FGFR4 (R)-KD were trypsin digested and subjected to phosphopeptide enrichment prior to LC-MS/MS analysis. **B)** Kaplan-Meier survival curve of patients with HER2 positive breast cancer (TCGA, PAM50 classification: HER2 enriched, cBioPortal for Cancer Genomics) visualizes the correlation between FGFR4 upregulation and poor overall survival (OS). **C)** Immunoblotting of HER2 and FGFR4 in a set of luminal and triple-negative breast (ER^-, PR^-, $HER2^-$) cancer cell lines. Long exposures of HER2 and FGFR4 corresponding to immunoblot in Fig. 2A. Arrow heads denote FGFR4, asterisks mark unspecific bands.

Figure S2

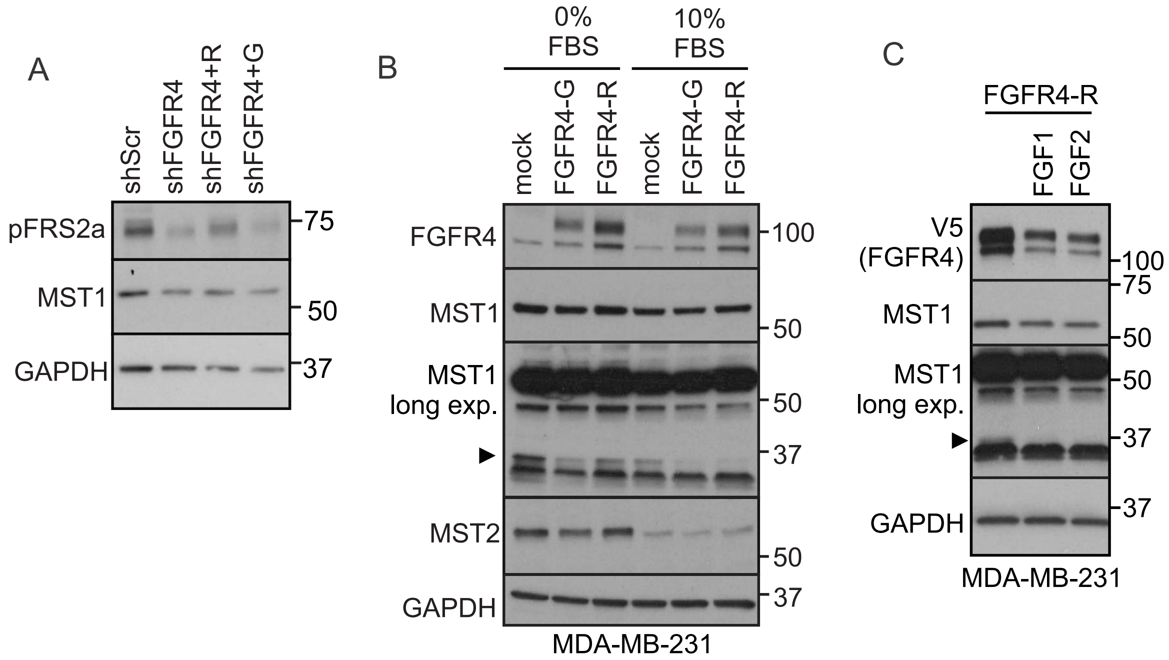

Figure S2. FGFR4 (R) overexpression restores phospho-FRS2α levels in MDA-MB-453 cells silenced for FGFR4. Reduced MST1 cleavage in FGFR4 overexpressing MDA-MB-231 cells. A) Immunoblots of phospho-FRS2α and MST1 of MDA-MB-453 rescue experiment shown in Fig. 3C. **B, C)** MDA-MB-231 cells transfected and selected for stable overexpression of FGFR4 (R) or (G) were cultured **(B)** without serum or with 10% FBS, or **(C)** stimulated with 10 ng/ml of FGF1 or FGF2 for 16 hours in serum-free medium, and subjected to immunoblotting. Arrow head points to a cleaved MST1 (~37 kDa).

Figure S3

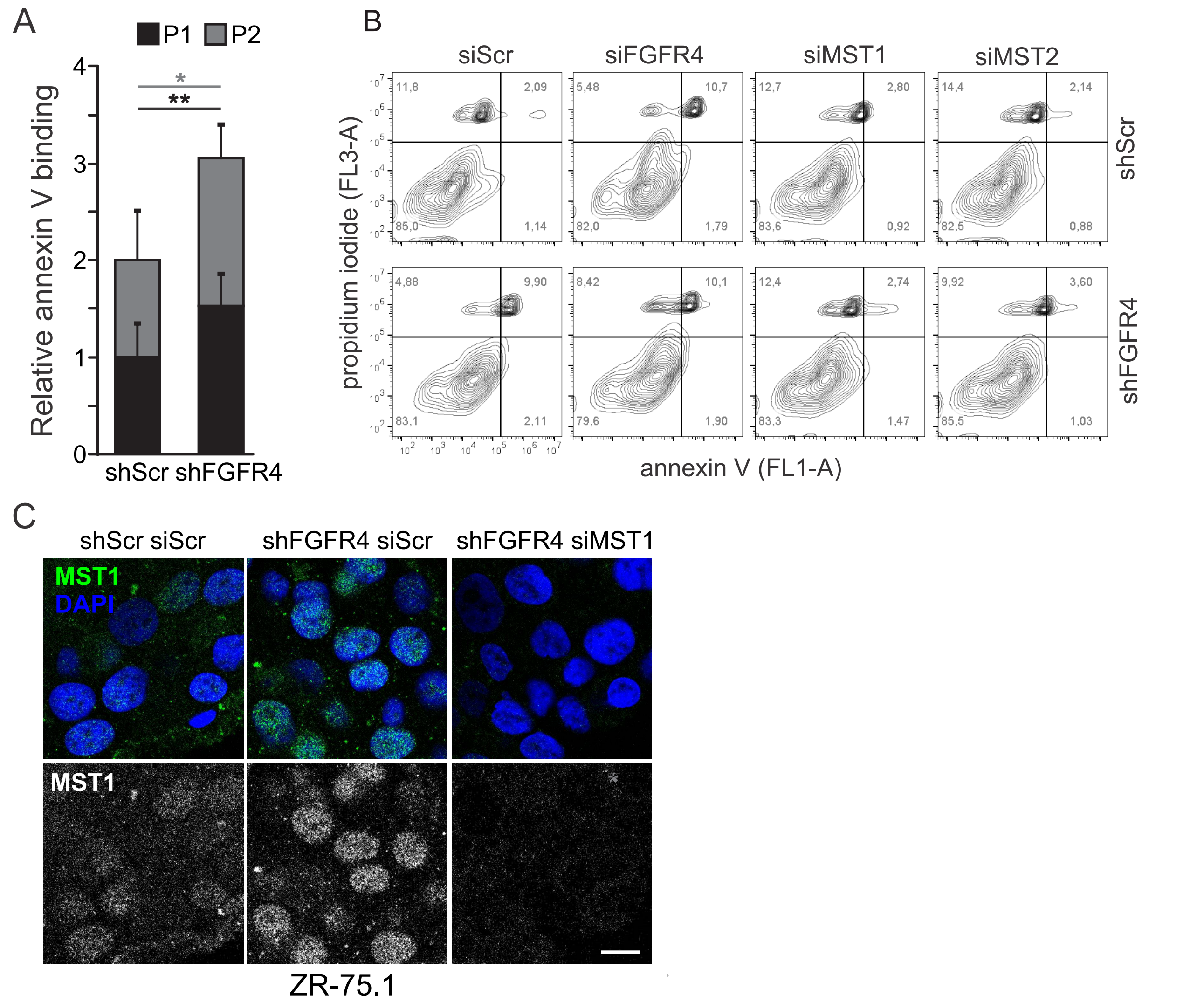

Figure S3. FGFR4 silencing increases apoptosis in breast cancer cells. MST1 localizes to nucleus upon FGFR4 silencing in ZR-75.1 cells. A) Flow cytometry analysis and quantification of relative annexin V binding in MDA-MB-453 cells in populations P1 and P2; * $P<0.05$; ** $P<0.01$. **B)** Quadrant gating for double-positive (annexin V (FL1-A) + PI (FL3-A)) MDA-MB-453 cells; percent of cells (of total 100 000) in the in the upper right quadrant was quantified to Fig. 4B for analysis of apoptosis in MDA-MB-453 cells transfected with indicated sh/siRNAs. **C)** ZR-75.1 cell spheres transduced with shScr or shFGFR4 shRNAs and transfected with indicated siRNAs were analyzed for MST1 localization by immunofluorescence.

Figure S4

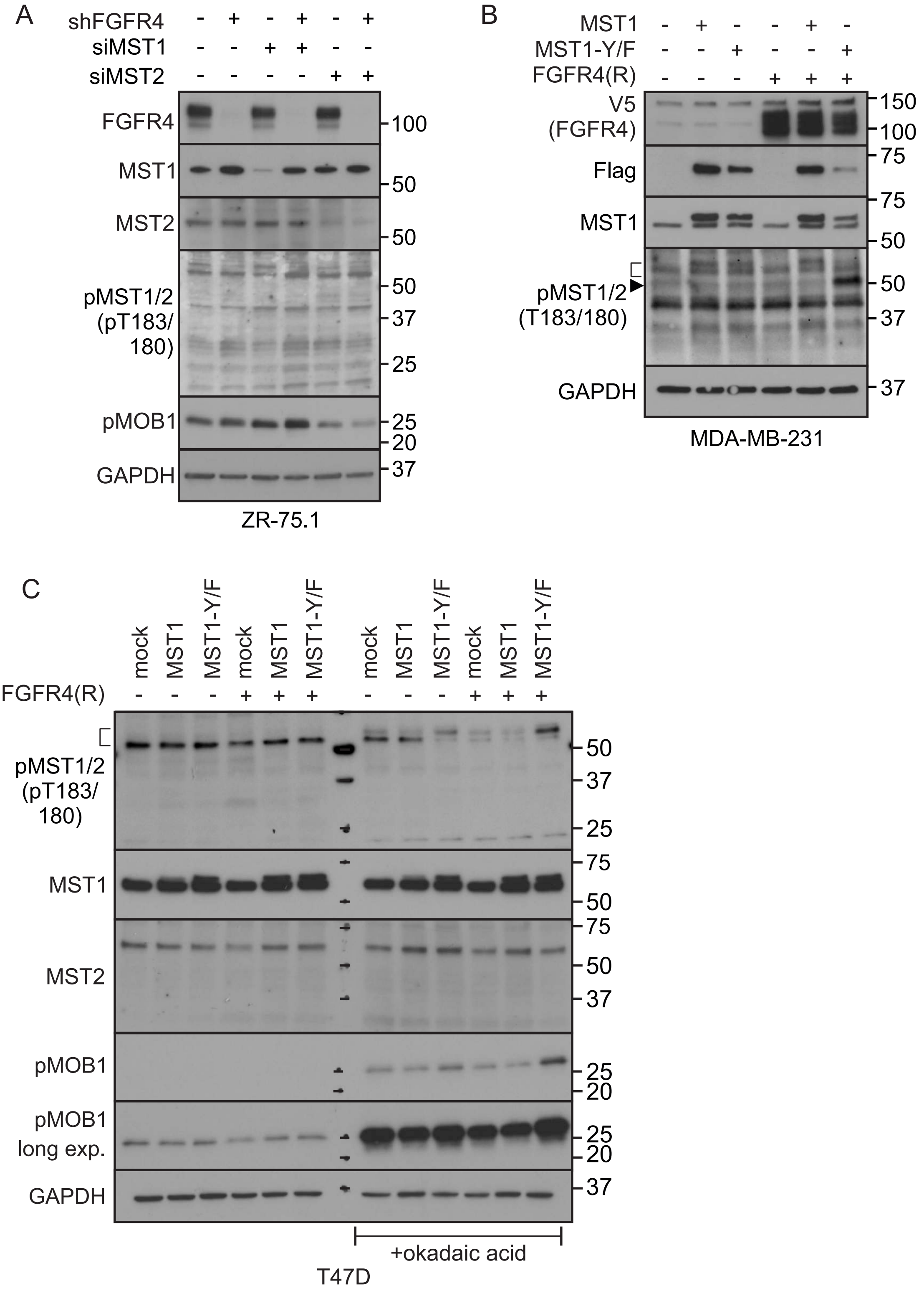

A
shFGFR4 - + - + - +
siMST1 - - + + - -
siMST2 - - - - + +
FGFR4
MST1
MST2
pMST1/2 (pT183/ 180)
pMOB1
GAPDH
100
50
50
50
37
25
25
20
37
ZR-75.1
B
MST1 - + - - + -
MST1-Y/F - - + - - +
FGFR4(R) - - - + + +
V5 (FGFR4)
Flag
MST1
pMST1/2 (T183/180)
GAPDH
150
100
75
75
50
50
37
37
MDA-MB-231
C
mock
MST1
MST1-Y/F
mock
MST1
MST1-Y/F
mock
MST1
MST1-Y/F
mock
MST1
MST1-Y/F
FGFR4(R) - - - + + + - - - + + +
pMST1/2 (pT183/ 180)
MST1
MST2
pMOB1
pMOB1 long exp.
GAPDH
50
37
25
75
50
75
50
37
25
20
25
20
37
+okadaic acid
T47D

Figure S4. MST2 and FGFR4 double depletion ablates pMOB1 activation and YAP regulation in ZR-75.1 spheres. Mutation of the site for FGFR4-mediated tyrosine phosphorylation restores MST1 activation in FGFR4 expressing cells. A) ZR-75.1 cell spheres transduced with shScr or shFGFR4 shRNAs, transfected with indicated siRNAs, and subjected to immunoblotting. Differential MST1 levels after identical knockdown in shScr vs. shFGFR4 cells are likely due to FGFR4-dependent suppression. **B)** MDA-MB-231 cells (co-)transfected with Flag-tagged wild-type or phosphosite mutant MST1-Y433F (Y/F) and FGFR4 (R) were subjected to immunoblotting as indicated. **B)** T47D cells (co-)transfected with wild-type or phosphosite mutant MST1-Y433F alone or with FGFR4 (R) were treated with 1 μM okadaic acid for one hour before cell lysis or left untreated, and subjected to immunoblotting. Note the prominent induction of pMOB1 upon okadaic acid treatment. Right side of the panel shown in Fig. 6B. **(B-C)** Brackets and arrow head indicate the activated pMST1/2 fragments.

Figure S5

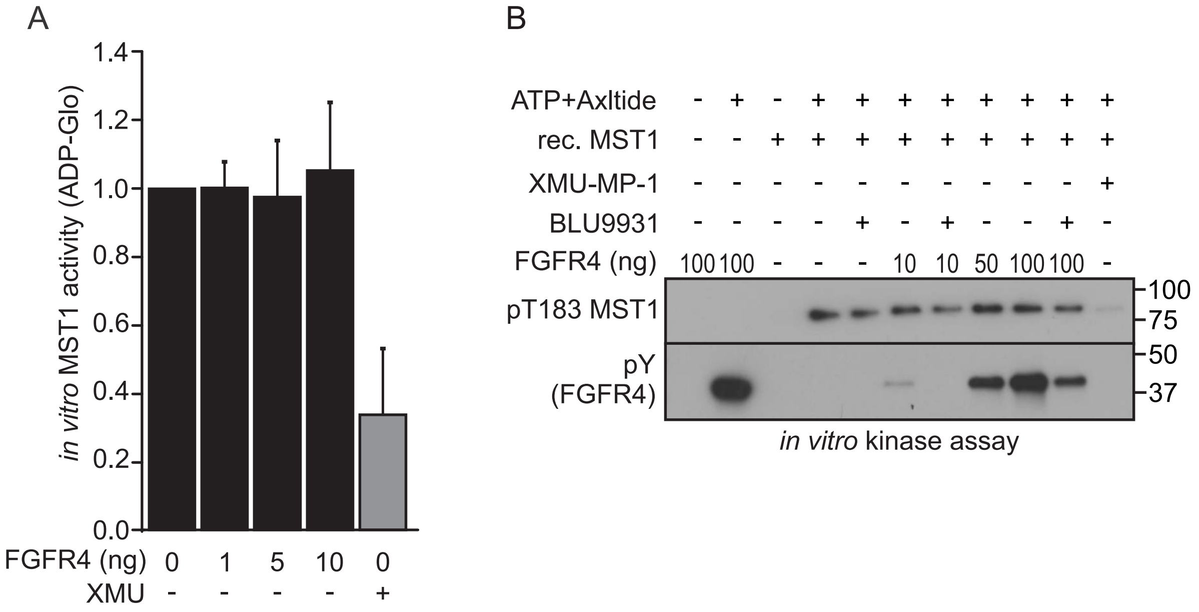

Figure S5. MST1 ***in vitro*** **kinase assays with active FGFR4 kinase. (A)** Relative MST1 activity measured with ADP-Glo kinase activity detection system in kinase reactions with increasing amounts of active FGFR4 kinase domain, or 1 μM MST1/2 inhibitor XMU-MP-1 (XMU). Mean ±SD of minimum two independent experiments is shown for each condition. **(B)** *In vitro* kinase assay detected by immunoblotting as indicated, 40 ng of MST1 kinase (83 kDa) and 0 - 100 ng recombinant kinase domain of FGFR4 (42.6 kDa) were (co-)incubated, with or without 1 μM XMU-MP-1 or 100 nM BLU9931 (FGFR4 inhibitor). Representative immunoblots out of two independent repeats are shown.

Figure S6

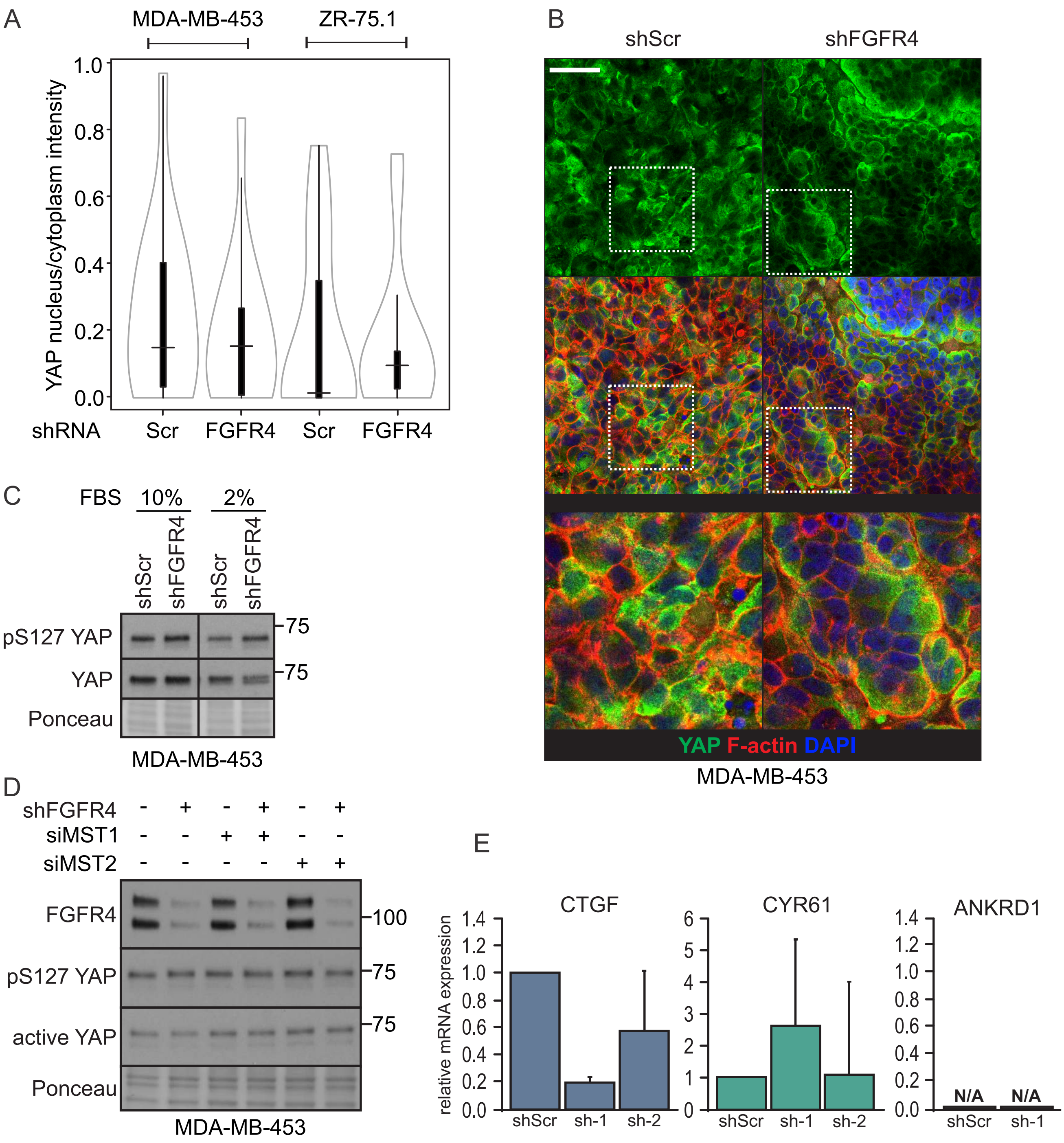
A
MDA-MB-453
ZR-75.1
YAP nucleus/cytoplasm intensity
1.0
0.8
0.6
0.4
0.2
0.0
shRNA
Scr
FGFR4
Scr
FGFR4
B
shScr
shFGFR4
YAP F-actin DAPI
MDA-MB-453
C
FBS
10%
2%
shScr
shFGFR4
shScr
shFGFR4
pS127 YAP
YAP
Ponceau
75
75
MDA-MB-453
D
shFGFR4 - + - + - +
siMST1 - - + + - -
siMST2 - - - - + +
FGFR4
pS127 YAP
active YAP
Ponceau
100
75
75
MDA-MB-453
E
CTGF
CYR61
ANKRD1
relative mRNA expression
shScr
sh-1
sh-2
N/A
N/A

Figure S6. FGFR4 has minor effects on YAP activation and function as transcriptional regulator in HER2$^+$ breast cancer cells. MDA-MB-453 cells transfected with indicated shRNAs/siRNAs were cultured on non-adherent plates as spheres of 24 000 cells. **(A)** Quantification of YAP nucleus/cytoplasma intensity ratio in MDA-MB-453 and ZR-75.1 spheres formed from cells transduced with shScr or shFGFR4 shRNAs. N=3 spheres from each condition, quantified with Anima (ref. 44) using four image fields (40X magnification) per sample. **(B)** Immunofluorescence for YAP and filamentous actin (phalloidin) of the MDA-MB-453 control and shFGFR4 cell spheres. High magnification of the marked (dashed white square) area shown on the lowest panel row; scale bar is 50 µm. **(C, D)** Immunoblotting of pS127, active and total YAP from MDA-MB-453 spheres transduced with indicated shRNAs/siRNAs cultured in medium with indicated serum percentage, and subjected to immunoblotting as indicated. **(E)** Quantitative real-time PCR for relative mRNA expression of canonical YAP target genes CTGF, CYR61 and ANKRD1 analyzed from shScr and shFGFR4 (sh-1 and sh-2 shRNAs) MDA-MB-453 spheres cultured in low serum (1% FBS) medium for 48 hours before RNA extraction. Mean ± SD of relative mRNA expression normalized to TATA-binding protein from two independent RNA extractions; except for ANKRD1, only one RNA sample was analyzed, and mRNA was non-detectable (N/A).

Figure S7

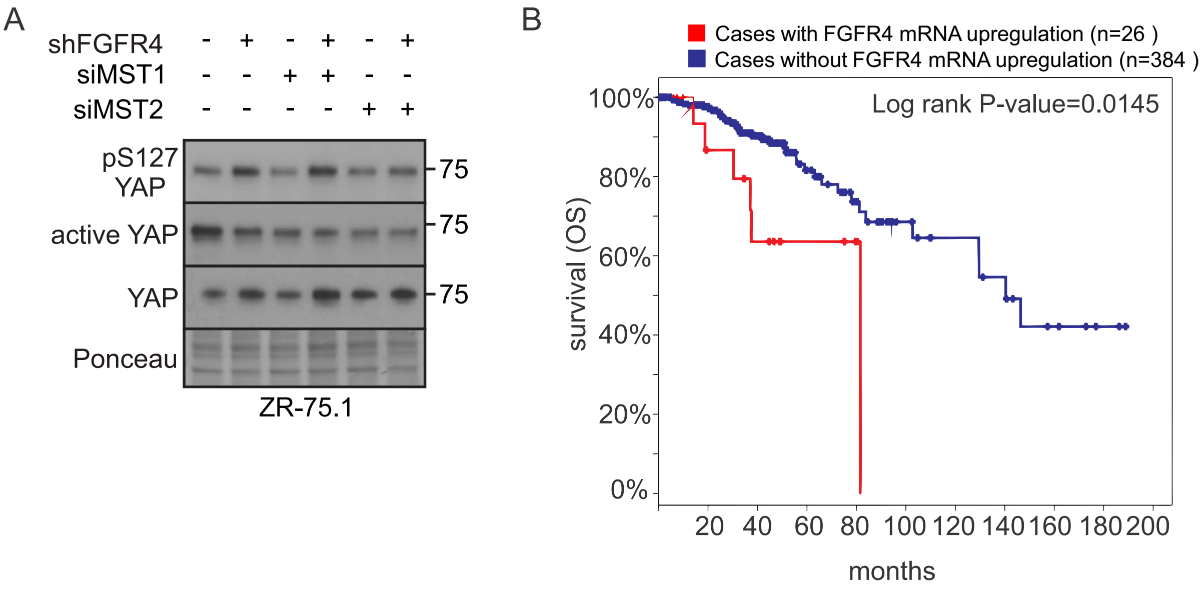

Figure S7. YAP regulation in ZR-75.1 cell spheres. FGFR4 survival association in TCGA breast cancer cohort. A) ZR-75.1 cells transfected with indicated shRNA/siRNAs were cultured as spheres in medium with 1% FBS for 48 hours, and subjected to immunoblotting. **(B)** Kaplan-Meier survival curve of patients included in the RPPA of the TCGA Nature 2012 breast cancer cohort (n=410 tumors [4], cBioPortal for Cancer Genomics [46, 47]) visualizes the correlation between FGFR4 alterations and poor overall survival (OS) in breast cancer.

Figure S8

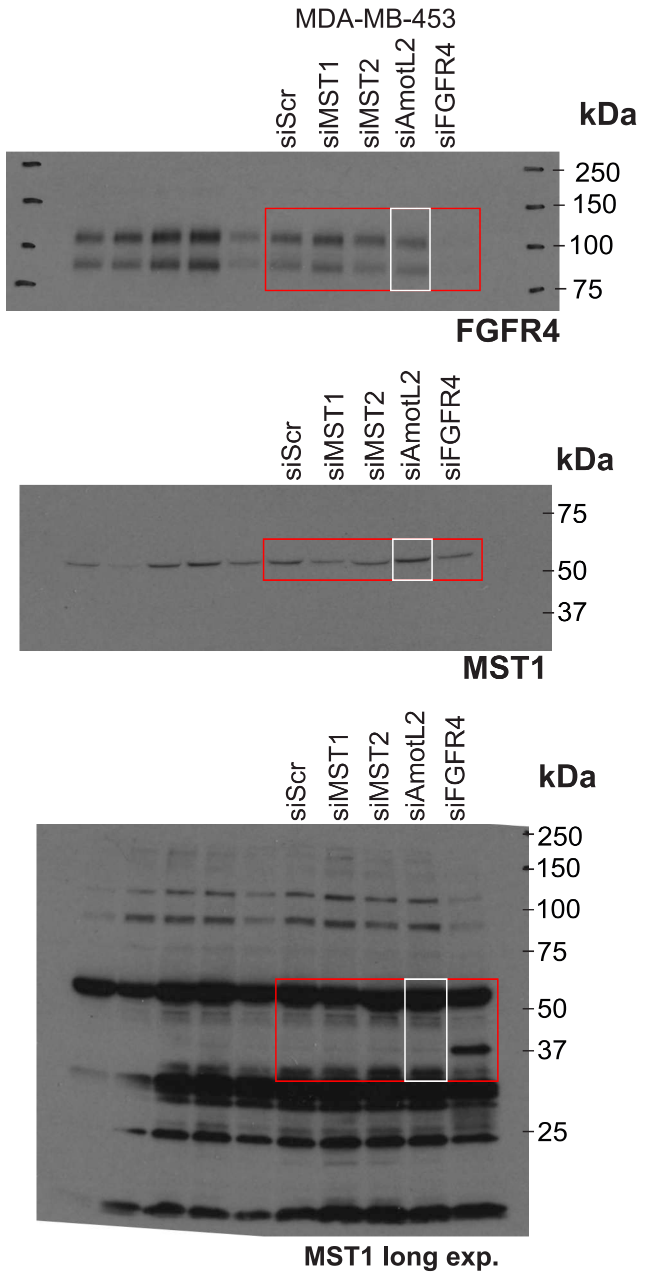

Figure S8. Uncropped immunoblots of Figure 3B, upper part. Areas inside white boxes not shown in Fig. 3B.
