# Supplementary material for: FGFR4 phosphorylates MST1 to confer breast cancer cells resistance to MST1/2-dependent apoptosis
Source: Cell Death Differ. 2019 Mar 22;26(12):2577–93. doi: 10.1038/s41418-019-0321-x (PMC7224384; doi:10.1038/s41418-019-0321-x)
Supplement: Supplementary file 2 — Table S1 [file 41418_2019_321_MOESM2_ESM.pdf]

Table S1. FGFR4 kinase substrate array hits. Related to Figure 1.

UniProt ID	gene	Database ID	Signal Rank	Z-Score	Description	Protein Amount	Signal	Background	Signal Used	Z-Factor	CI P-Value	CV	Significance Call	Array ID
Q13188	STK3	BC010640.1	1	10.95646	serine/threonine kinase 3 (STE20 homolog, yeast) (STK3); KRS1; MST2	17545.2	65143	44644	49317.7	0.70818	0.0009912	0.03652	Hit	B38R09C17
P41743	PRKCI	NM_002740.1	2	10.58019	protein kinase C, iota (PRKCI)	35214.7	62546	32788	47677.9	0.81261	0.0010609	0.0207	Hit	B17R10C08
Q9BZL6	PRKD2	NM_016457.3	3	10.42198	protein kinase D2 (PRKD2), transcript variant 1	42697.1	64481	42900	46988.5	0.73502	0.0010925	0.065	Hit	B18R07C06
P48730	CSNK1D	PV3665	4	10.42083	casein kinase 1, delta (CSNK1D), transcript variant 1	51746.7	65166	43083	46983.5	0.77308	0.0010927	0.06722	Hit	B05R16C01
Q13188	STK3	BC010640.1	5	10.38664	serine/threonine kinase 3 (STE20 homolog, yeast) (STK3)	19790.2	61449	29096	46834.5	0.70818	0.0010997	0.03652	Hit	B38R09C18
Q13043	STK4	NM_006282.1	6	10.26847	Serine/threonine-protein kinase 4 (STK4); KRS2; MST1; YSK3	20310.3	59358	32674	46319.5	0.79117	0.0011245	0.04635	Hit	B18R10C01
P41743	PRKCI	NM_002740.1	7	10.26452	protein kinase C, iota (PRKCI)	36117.7	65470	45344	46302.3	0.81261	0.0011253	0.0207	Hit	B17R10C07
P00533	EGFR(ErbB1),	PV4879	8	10.20165	epidermal growth factor receptor (erythroblastic leukemia viral (v-erb-b) oncogene homolog, avian) (EGFR); see catalog	15100.1	63614	42454	46028.3	0.90529	0.0011388	0.02507	Hit	B09R16C05
P43405	SYK	BC011399.1	9	10.17188	spleen tyrosine kinase (SYK)	30891.9	63993	42663	45898.6	0.64256	0.0011453	0.00867	Hit	B06R10C06
O14757	CHEK1	NM_001274.2	10	10.0886	CHK1 checkpoint homolog (S. pombe) (CHEK1)	18864.2	59924	35580	45535.7	0.84394	0.0011637	0.01355	Hit	B11R10C08
P48730	CSNK1D	NM_139062.1	11	10.0656	casein kinase 1, delta (CSNK1D), transcript variant 2	13372.4	64668	50363	45435.4	0.8227	0.0011689	0.0295	Hit	B16R09C07
P43405	SYK	BC011399.1	12	10.04359	spleen tyrosine kinase (SYK)	29970.7	65511	56385	45339.5	0.64256	0.0011739	0.00867	Hit	B06R10C05
Q05513	PRKCZ	BC008058.1	13	9.91482	protein kinase C, zeta (PRKCZ)	13663.2	65187	50523	44778.3	0.86089	0.0012037	0.02874	Hit	B02R16C13
Q13188	STK3	PV4805	14	9.90603	serine/threonine kinase 3 (STE20 homolog, yeast) (STK3)	15343.5	65463	51332	44740	0.6822	0.0012058	0.00264	Hit	B07R15C17
O14757	CHEK1	NM_001274.2	15	9.89026	CHK1 checkpoint homolog (S. pombe) (CHEK1)	15376.7	64608	42934	44671.3	0.84394	0.0012095	0.01355	Hit	B11R10C07
Q13043	STK4	PV3854	16	9.87564	Serine/threonine-protein kinase 4	19108.1	65535	54896	44607.6	0.85343	0.001213	0.04016	Hit	B39R15C17
Q13188	STK3	PV4805	17	9.86781	serine/threonine kinase 3 (STE20 homolog, yeast) (STK3)	6011.57	64261	38571	44573.5	0.6822	0.0012149	0.00264	Hit	B07R15C18
P00533	EGFR	PV4879	18	9.83374	epidermal growth factor receptor (erythroblastic leukemia viral (v-erb-b) oncogene homolog, avian) (EGFR); see catalog	14581.6	58699	26933	44425	0.90529	0.001223	0.02507	Hit	B09R16C06
Q9HCP0	CSNK1G1	NM_022048.1	19	9.78185	casein kinase 1, gamma 1 (CSNK1G1)	24564.8	65327	44496	44198.9	0.74624	0.0012357	0.03574	Hit	B46R09C11
P48730	CSNK1D	NM_139062.1	20	9.63959	casein kinase 1, delta (CSNK1D), transcript variant 2	14301.3	64908	51417	43578.9	0.8227	0.0012713	0.0295	Hit	B16R09C08
O96013	PAK4	NM_005884.2	21	9.6078	p21(CDKN1A)-activated kinase 4 (PAK4), transcript variant 1	51826.7	63246	47340	43440.4	0.53375	0.0012795	0.088	Hit	B40R03C09
Q13043	STK4	NM_006282.1	22	9.59391	Serine/threonine-protein kinase 4	19697.5	57974	27694	43379.8	0.79117	0.0012831	0.04635	Hit	B18R10C02
P78368	CSNK1G2	BC020972.1	23	9.53777	Casein kinase I isoform gamma-2	30347.4	59367	29464	43135.2	0.78455	0.0012978	0.00458	Hit	B22R09C18
Q05513	PRKCZ	BC008058.1	24	9.50548	protein kinase C, zeta (PRKCZ)	14295.5	61364	38445	42994.5	0.86089	0.0013063	0.02874	Hit	B02R16C14
P49761	CLK3	NM_003992.1	25	9.47829	Dual specificity protein kinase CLK3	33120.6	61675	50962	42876	0.62415	0.0013136	0.01573	Hit	B07R09C22
Q9BZL6	PRKD2	NM_016457.3	26	9.47443	protein kinase D2 (PRKD2), transcript variant 1	33840.8	65535	57883	42859.2	0.73502	0.0013147	0.065	Hit	B18R07C05
P78368	CSNK1G2	BC020972.1	27	9.47385	Casein kinase I isoform gamma-2	27242.4	61649	42054	42856.7	0.78455	0.0013148	0.00458	Hit	B22R09C17
P48730	CSNK1D	PV3665	28	9.44242	casein kinase 1, delta (CSNK1D), transcript variant 1	50728.2	62528	39564	42719.7	0.77308	0.0013233	0.06722	Hit	B05R16C02
Q13043	STK4	PV3854	29	9.31028	Serine/threonine-protein kinase 4	18969.6	65093	43557	42143.8	0.85343	0.00136	0.04016	Hit	B39R15C18
Q9HCP0	CSNK1G1	NM_022048.1	30	9.28187	casein kinase 1, gamma 1 (CSNK1G1)	23105	59180	26457	42020	0.74624	0.0013681	0.03574	Hit	B46R09C12
P49761	CLK3	NM_003992.1	31	9.26187	Dual specificity protein kinase CLK3	25784.5	65500	65474	41932.8	0.62415	0.0013738	0.01573	Hit	B07R09C21
Q05655	PRKCD	NM_006254.3	32	9.25774	protein kinase C, delta (PRKCD), transcript variant 1	15398.1	53963	24585	41914.9	0.52803	0.001375	0.03278	Hit	B06R17C21
P48729	CSNK1A1	PV3850	33	9.2186	casein kinase 1, alpha 1 (CSNK1A1), transcript variant 1	38914.9	55077	31361	41744.3	0.92425	0.0013863	0.02071	Hit	B45R15C17
O75914	PAK3	NM_002578.1	34	9.19606	p21 (CDKN1A)-activated kinase 3 (PAK3)	15337.6	64283	54105	41646.1	0.80377	0.0013929	0.04129	Hit	B20R10C03
Q6IQ55	TTBK2	BC041876.1	35	9.15598	tau tubulin kinase 2 (TTBK2)	2193.6	62653	52689	41471.4	0.70819	0.0014047	0.06363	Hit	B16R08C09
Q9HCP0	CSNK1G1	PV3825	36	8.96583	casein kinase 1, gamma 1 (CSNK1G1)	20692.1	51954	25830	40642.7	0.81989	0.001463	0.05044	Hit	B05R15C21
P48729	CSNK1A1	PV3850	37	8.94207	casein kinase 1, alpha 1 (CSNK1A1), transcript variant 1	38003.4	56009	26302	40539.2	0.92425	0.0014706	0.02071	Hit	B45R15C18
O15530	PDPK1	NM_002613.3	38	8.94092	3-phosphoinositide dependent protein kinase-1 (PDPK1), transcript variant 1	42740.1	54947	31359	40534.2	0.83114	0.0014709	0.01303	Hit	B11R04C21
O55047	TLK2	NM_006852.1	39	8.88613	Serine/threonine-protein kinase tousled-like 2	30478.1	60366	48038	40295.4	0.81374	0.0014885	0.01623	Hit	B28R09C21
P54762	EPHB1	NM_004441.2	40	8.84811	Ephrin type-B receptor 1	27738.1	55016	32144	40129.7	0.85872	0.0015009	0.02373	Hit	B43R10C01
Q9P0L2	MARK1	PV4395	41	8.84543	Serine/threonine-protein kinase MARK1	10113.3	59373	43692	40118.1	0.81231	0.0015018	0.0377	Hit	B24R16C03
Q05655	PRKCD	NM_006254.3	42	8.82195	protein kinase C, delta (PRKCD), transcript variant 1	24246.7	50186	14549	40015.7	0.52803	0.0015096	0.03278	Hit	B06R17C22
P42684	ABL2	BC065912.1	43	8.7878	Tyrosine-protein kinase ABL2	5565.47	50136	18037	39866.9	0.71248	0.0015209	0.08596	Hit	B47R19C01
O15530	PDPK1	NM_002613.3	44	8.77113	3-phosphoinositide dependent protein kinase-1 (PDPK1), transcript variant 1	73834.9	51458	19514	39794.3	0.83114	0.0015265	0.01303	Hit	B11R04C22
P48729	CSNK1A1	NM_001892.4	45	8.75793	casein kinase 1, alpha 1 (CSNK1A1), transcript variant 2	10772.8	55298	30770	39736.7	0.9148	0.001531	0.02348	Hit	B13R18C21
Q9UK32	RPS6KA6	NM_014496.1	46	8.70256	ribosomal protein S6 kinase, 90kDa, polypeptide 6 (RPS6KA6)	10930.9	51582	23951	39495.4	0.87811	0.0015499	0.03031	Hit	B15R19C09
O55047	TLK2	NM_006852.1	47	8.67625	Serine/threonine-protein kinase tousled-like 2	28374.3	55917	33455	39380.8	0.81374	0.001559	0.01623	Hit	B28R09C22
O75914	PAK3	NM_002578.1	48	8.65384	p21 (CDKN1A)-activated kinase 3 (PAK3)	14303.3	60167	42362	39283.1	0.80377	0.0015668	0.04129	Hit	B20R10C04
P49841	GSK3B	BC012760.2	49	8.63666	Glycogen synthase kinase-3 beta	33142.1	50580	26116	39208.2	0.80394	0.0015728	0.05536	Hit	B05R18C17
Q99683	MAP3K5	NM_005923.3	51	8.55161	mitogen-activated protein kinase kinase kinase 5 (MAP3K5)	4824.36	50793	19125	38837.6	0.78689	0.0016032	0.06011	Hit	B35R05C13
P54762	EPHB1	NM_004441.2	52	8.54421	Ephrin type-B receptor 1	30641.4	62396	45546	38805.4	0.85872	0.0016059	0.02373	Hit	B43R10C02
P48729	CSNK1A1	NM_001892.4	53	8.4601	casein kinase 1, alpha 1 (CSNK1A1), transcript variant 2	8951.99	51810	20664	38438.8	0.9148	0.0016369	0.02348	Hit	B13R18C22
O96013	PAK4	NM_005884.2	54	8.43994	p21(CDKN1A)-activated kinase 4 (PAK4), transcript variant 1	127574	56657	33203	38351	0.53375	0.0016445	0.088	Hit	B40R03C10
Q14164	IKBKE	PV4875	55	8.40763	Inhibitor of nuclear factor kappa-B kinase subunit epsilon	10132.7	49367	16986	38210.2	0.5431	0.0016567	0.02716	Hit	B07R16C04
Q9P0L2	MARK1	PV4395	56	8.36731	Serine/threonine-protein kinase MARK1	11519.2	55909	32301	38034.4	0.81231	0.0016722	0.0377	Hit	B24R16C04
P53779	MAPK10	PV4563	57	8.36665	mitogen-activated protein kinase 10 (MAPK10), transcript variant 1	7918.98	50283	22695	38031.6	0.76917	0.0016724	0.06688	Hit	B27R15C13
P49674	CSNK1E	NM_001894.2	58	8.3634	casein kinase 1, epsilon (CSNK1E), transcript variant 2	12230.7	49044	19312	38017.4	0.93067	0.0016737	0.0139	Hit	B23R10C09
Q6IQ55	TTBK2	BC041876.1	59	8.33653	tau tubulin kinase 2 (TTBK2)	2355.58	56301	36911	37900.3	0.70819	0.0016841	0.06363	Hit	B16R08C10
Q9HCP0	CSNK1G1	PV3825	60	8.32345	casein kinase 1, gamma 1 (CSNK1G1)	16959.3	48079	20180	37843.3	0.81989	0.0016892	0.05044	Hit	B05R15C22
Q9UK32	RPS6KA6	NM_014496.1	61	8.32218	ribosomal protein S6 kinase, 90kDa, polypeptide 6 (RPS6KA6)	10427.4	48311	15575	37837.8	0.87811	0.0016897	0.03031	Hit	B15R19C10
P00533	EGFR(ErbB1),I	PV4128	62	8.24637	epidermal growth factor receptor (erythroblastic leukemia viral (v-erb-b) oncogene homolog, avian) (EGFR); see catalog	14484.8	51281	27945	37507.4	0.76286	0.0017199	0.07366	Hit	B13R15C21
P49674	CSNK1E	NM_001894.2	63	8.19359	casein kinase 1, epsilon (CSNK1E), transcript variant 2	11272.4	47397	15342	37277.4	0.93067	0.0017413	0.0139	Hit	B23R10C10

Q05655	PRKCD	P2287	64	8.18036	protein kinase C, delta (PRKCD), transcript variant 1	0	49232	22240	37219.7	0.68084	0.0017467	0.09549	Hit	B05R15C15
Q13464	ROCK1	PV3691	65	8.07747	Rho-associated, coiled-coil containing protein kinase 1 (ROCK1)	5061.49	51526	26329	36771.4	0.96117	0.0017899	0.00348	Hit	B23R15C21
Q14164	IKBKE	PV4875	66	8.07723	Inhibitor of nuclear factor kappa-B kinase subunit epsilon	10671.4	50375	27252	36770.3	0.5431	0.00179	0.02716	Hit	B07R16C03
P00519	ABL1	PV3865	67	8.06838	v-abl Abelson murine leukemia viral oncogene homolog 1 (ABL1), transcript variant a; see catalog number for detailed i	0	56212	39348	36731.7	0.61148	0.0017938	0.05227	Hit	B40R15C13
Q13464	ROCK1	PV3691	68	8.03609	Rho-associated, coiled-coil containing protein kinase 1 (ROCK1)	6012.88	47885	22131	36591	0.96117	0.0018078	0.00348	Hit	B23R15C22
P78368	CSNK1G2	NM_001319.5	69	8.02381	casein kinase 1, gamma 2 (CSNK1G2)	17776.7	48788	20245	36537.5	0.78578	0.0018131	0.05674	Hit	B33R02C21
Q9UHD2	TBK1	NM_013254.2	70	7.98477	TANK-binding kinase 1 (TBK1)	8695.44	46422	16654	36367.4	0.59918	0.0018302	0.10562	Hit	B43R03C01
P24723	PRKCH	P2634	71	7.96385	protein kinase C, eta	0	48364	17418	36276.2	0.80538	0.0018395	0.03861	Hit	B43R16C02
P49841	GSK3B	BC012760.2	72	7.95877	Glycogen synthase kinase-3 beta	42431.7	46537	16197	36254.1	0.80394	0.0018418	0.05536	Hit	B05R18C18
O75716	STK16	NM_003691.1	73	7.93417	Serine/threonine-protein kinase 16	33352.8	47785	20869	36146.9	0.79301	0.0018528	0.03934	Hit	B18R10C07
Q9H2X6	HIPK2	PV5275	74	7.87323	homeodomain interacting protein kinase 2	14418.4	49812	27014	35881.3	0.78847	0.0018806	0.04245	Hit	B20R16C05
Q99683	MAP3K5	NM_005923.3	75	7.82491	mitogen-activated protein kinase kinase kinase 5 (MAP3K5)	4404.76	46581	14396	35670.7	0.78689	0.001903	0.06011	Hit	B35R05C14
Q05513	PRKCZ	P2268	76	7.78088	protein kinase C, zeta (PRKCZ), transcript variant 1	0	48253	25147	35478.8	0.90826	0.0019238	0.02506	Hit	B13R15C17
Q9Y6E0	STK24	BC035578.1	77	7.74014	Serine/threonine-protein kinase 24; STE20; MST3; STK3	33419.3	46443	15146	35301.3	0.68798	0.0019434	0.01988	Hit	B38R17C22
P42684	ABL2	BC065912.1	78	7.73943	Tyrosine-protein kinase ABL2	5150.27	45670	9864	35298.2	0.71248	0.0019437	0.08596	Hit	B47R19C02
Q8TD19	NEK9	PV4653	79	7.69638	NIMA (never in mitosis gene a)- related kinase 9	2966.43	47612	25236	35110.6	0.85356	0.0019647	0.0385	Hit	B03R16C03
P00533	EGFR	PV3872	80	7.65525	epidermal growth factor receptor (erythroblastic leukemia viral (v-erb-b) oncogene homolog, avian) (EGFR); see catalog	4499.64	46945	16218	34931.4	0.82786	0.0019851	0.04538	Hit	B35R15C22
P43405	SYK	NM_003177.3	81	7.62768	spleen tyrosine kinase (SYK)	35603	54912	38078	34811.2	0.76447	0.0019989	0.05482	Hit	B44R05C19
Q8N752	CSNK1A1L	BC028723.1	83	7.58106	casein kinase 1, alpha 1-like (CSNK1A1L)	11114.4	46062	23067	34608.1	0.61412	0.0020227	0.07198	Hit	B28R17C01
P53779	MAPK10	PV4563	84	7.57854	mitogen-activated protein kinase 10 (MAPK10), transcript variant 1	8413.35	46436	15157	34597.1	0.76917	0.002024	0.06688	Hit	B27R15C14
Q04759	PRKCQ	P2996	85	7.53876	protein kinase C, theta (PRKCQ)	0	47042	22988	34423.7	0.94006	0.0020446	0.00886	Hit	B19R15C17
P08581	MET	PV3143	86	7.53687	met proto-oncogene (hepatocyte growth factor receptor) (MET); see catalog number for detailed information on wild-typ	0	43226	20160	34415.5	0.70529	0.0020456	0.0923	Hit	B13R16C01
P24723	PRKCH	P2634	87	7.52139	protein kinase C, eta	0	45592	21452	34348	0.80538	0.0020537	0.03861	Hit	B43R16C01
Q9Y6E0	STK24	BC035578.1	88	7.5156	Serine/threonine-protein kinase 24	31777.2	46139	19117	34322.8	0.68798	0.0020567	0.01988	Hit	B38R17C21
Q96RR4	CAMKK2	BC026060.2	89	7.51082	calcium/calmodulin-dependent protein kinase kinase 2, beta (CAMKK2)	30324	51295	33937	34302	0.77148	0.0020592	0.04386	Hit	B36R08C13
Q05513	PRKCZ	P2268	90	7.49733	protein kinase C, zeta (PRKCZ), transcript variant 1	0	45185	19162	34243.2	0.90826	0.0020664	0.02506	Hit	B13R15C18
O75716	STK16	NM_003691.1	91	7.48522	Serine/threonine-protein kinase 16	38626.1	44070	13574	34190.4	0.79301	0.0020728	0.03934	Hit	B18R10C08
P00519	ABL1	PV3865	92	7.46756	v-abl Abelson murine leukemia viral oncogene homolog 1 (ABL1), transcript variant a; see catalog number for detailed i	0	53715	33310	34113.5	0.61148	0.0020822	0.05227	Hit	B40R15C14
Q04759	PRKCQ	P2996	93	7.44039	protein kinase C, theta (PRKCQ)	0	44559	14456	33995	0.94006	0.0020969	0.00886	Hit	B19R15C18
P21709	EPHA1	PV3841	94	7.40641	Ephrin receptor A1 (EPHA1)	11797.4	52608	32761	33847	0.80668	0.0021154	0.02599	Hit	B48R15C18
P00533	EGFR(ErbB1),I	PV4128	95	7.39413	epidermal growth factor receptor (erythroblastic leukemia viral (v-erb-b) oncogene homolog, avian) (EGFR); see catalog	11561.3	45819	18585	33793.4	0.76286	0.0021222	0.07366	Hit	B13R15C22
Q9H2X6	HIPK2	PV5275	96	7.39331	homeodomain interacting protein kinase 2	10009.7	45933	19085	33789.9	0.78847	0.0021227	0.04245	Hit	B20R16C06
P78368	CSNK1G2	NM_001319.5	97	7.37701	casein kinase 1, gamma 2 (CSNK1G2)	22645.3	44400	10344	33718.8	0.78578	0.0021317	0.05674	Hit	B33R02C22
Q92630	DYRK2	BC006375.2	98	7.35022	dual-specificity tyrosine-(Y)-phosphorylation regulated kinase 2 (DYRK2)	9330.6	46348	24004	33602.1	0.75481	0.0021466	0.06096	Hit	B14R05C11
Q8TD19	NEK9	PV4653	100	7.26937	NIMA (never in mitosis gene a)- related kinase 9	3848.56	44216	15981	33249.7	0.85356	0.0021928	0.0385	Hit	B03R16C04
P15735	PHKG2	NM_000294.1	101	7.22452	phosphorylase kinase, gamma 2 (testis)	10771.6	42751	16955	33054.3	0.73702	0.002219	0.06913	Hit	B25R02C19
P17252	PRKCA	NM_002737.1	102	7.20049	protein kinase C, alpha (PRKCA); see catalog number for detailed information on wild-type or point mutant status	39855.3	43911	18933	32949.6	0.88526	0.0022333	0.03266	Hit	B21R02C15
Q9WTQ1	PRKD1	PV3791	103	7.17433	Serine/threonine-protein kinase D1	3013.27	45265	21734	32835.6	0.52591	0.0022489	0.01349	Hit	B07R15C13
P00533	EGFR	PV3872	104	7.15683	epidermal growth factor receptor (erythroblastic leukemia viral (v-erb-b) oncogene homolog, avian) (EGFR); see catalog	4253.68	47793	26434	32759.3	0.82786	0.0022595	0.04538	Hit	B35R15C21
O00506	STK25	NM_006374.2	105	7.13237	serine/threonine kinase 25 (STE20 homolog, yeast) (STK25); SOK1; YSK1	34972.3	44724	20848	32652.7	0.55795	0.0022744	0.05271	Hit	B22R14C13
P21709	EPHA1	PV3841	106	7.12604	Ephrin receptor A1 (EPHA1)	10728	54011	36585	32625.1	0.80668	0.0022783	0.02599	Hit	B48R15C17
Q9Y6E0	STK24	NM_003576.2	107	7.10665	Serine/threonine-protein kinase 24	13473.9	42389	11133	32540.6	0.86808	0.0022902	0.02319	Hit	B14R09C22
Q05655	PRKCD	P2287	108	7.09991	protein kinase C, delta (PRKCD), transcript variant 1	0	45044	19654	32511.3	0.68084	0.0022944	0.09549	Hit	B05R15C16
O15530	PDPK1	P3001	109	7.08855	3-phosphoinositide dependent protein kinase-1 (PDPK1), transcript variant 1	0	43258	21870	32461.8	0.62473	0.0023015	0.10284	Hit	B14R15C15
Q96RR4	CAMKK2	BC026060.2	110	7.03728	calcium/calmodulin-dependent protein kinase kinase 2, beta (CAMKK2)	34487.6	47986	28475	32238.3	0.77148	0.0023338	0.04386	Hit	B36R08C14
Q9WTQ1	PRKD1	PV3791	111	7.03197	Serine/threonine-protein kinase D1	3528.49	46320	20466	32215.2	0.52591	0.0023372	0.01349	Hit	B07R15C14
P43405	SYK	NM_003177.3	112	7.03149	spleen tyrosine kinase (SYK)	36916.5	51160	31520	32213.1	0.76447	0.0023375	0.05482	Hit	B44R05C20
P00519	ABL1	NM_005157.2	113	7.02685	v-abl Abelson murine leukemia viral oncogene homolog 1 (ABL1), transcript variant a; see catalog number for detailed i	34195.9	47338	32997	32192.9	0.74777	0.0023404	0.05463	Hit	B01R09C19
Q9BUB5	MKNK1	NM_003684.2	114	6.99422	MAP kinase interacting serine/threonine kinase 1 (MKNK1), transcript variant 1	10142.5	44023	19915	32050.7	0.86581	0.0023614	0.03199	Hit	B15R02C11
P54762	EPHB1	PV3786	115	6.99202	Ephrin receptor B1 (EPHB1)	12734.5	42115	18839	32041.1	0.89139	0.0023629	0.03047	Hit	B21R15C17
Q9NWZ3	IRAK4	PV3362	116	6.97719	interleukin-1 receptor-associated kinase 4 (IRAK4)	0	42582	26349	31976.5	0.76063	0.0023725	0.07004	Hit	B12R15C22
P08922	ROS1	PV3814	117	6.96987	v-ros UR2 sarcoma virus oncogene homolog 1 (avian) (ROS1)	2447.55	46021	29650	31944.5	0.90233	0.0023773	0.00237	Hit	B24R15C13
P08922	ROS1	PV3814	118	6.94533	v-ros UR2 sarcoma virus oncogene homolog 1 (avian) (ROS1)	2625.99	44523	22111	31837.6	0.90233	0.0023934	0.00237	Hit	B24R15C14
P29376	LTK	PV4651	119	6.94142	leukocyte tyrosine kinase (LTK), transcript variant 1	18878.7	43519	19280	31820.6	0.84644	0.002396	0.03256	Hit	B25R15C17
Q9Y6E0	STK24	NM_003576.2	121	6.8657	Serine/threonine-protein kinase 24	15183.5	43007	21702	31490.6	0.86808	0.002447	0.02319	Hit	B14R09C21
P17252	PRKCA	NM_002737.1	122	6.85913	protein kinase C, alpha (PRKCA); see catalog number for detailed information on wild-type or point mutant status	24235	41603	9944	31462	0.88526	0.0024514	0.03266	Hit	B21R02C16
Q9UHD2	TBK1	NM_013254.2	123	6.82492	TANK-binding kinase 1 (TBK1)	4408.01	41323	10037	31312.9	0.59918	0.0024751	0.10562	Hit	B43R03C02
Q8N752	CSNK1A1L	BC028723.1	124	6.81184	casein kinase 1, alpha 1-like (CSNK1A1L)	10450.8	42526	15784	31255.9	0.61412	0.0024842	0.07198	Hit	B28R17C02
O75914	PAK3	PV3789	125	6.72429	p21 (CDKN1A)-activated kinase 3 (PAK3)	0	39632	13781	30874.3	0.89296	0.0025465	0.02316	Hit	B19R15C22
Q92630	DYRK2	BC006375.2	126	6.71294	dual-specificity tyrosine-(Y)-phosphorylation regulated kinase 2 (DYRK2)	8448.48	42630	14116	30824.9	0.75481	0.0025548	0.06096	Hit	B14R05C12
P54762	EPHB1	PV3786	128	6.68189	Ephrin receptor B1 (EPHB1)	15640.2	40356	12083	30689.6	0.89139	0.0025776	0.03047	Hit	B21R15C18
Q9BUB5	MKNK1	NM_003684.2	129	6.66883	MAP kinase interacting serine/threonine kinase 1 (MKNK1), transcript variant 1	27513.7	41739	14942	30632.7	0.86581	0.0025872	0.03199	Hit	B15R02C12
P78362	SRPK2	NM_182691.1	130	6.66343	SFRS protein kinase 2 (SRPK2), transcript variant 2	16776	39852	15927	30609.1	0.87047	0.0025913	0.01838	Hit	B29R20C19
P49841	GSK3B	NM_002093.2	131	6.65852	glycogen synthase kinase 3 beta (GSK3B)	32736.1	41183	13268	30587.7	0.91048	0.0025949	0.01647	Hit	B35R03C03
O00506	STK25	PV3657	132	6.65712	serine/threonine kinase 25 (STE20 homolog, yeast) (STK25)	8654.45	40068	16587	30581.6	0.81228	0.002596	0.05432	Hit	B34R15C15
P21802	FGFR2	NM_022972.1	133	6.63737	Fibroblast growth factor receptor 2	6676.42	49907	37282	30495.5	0.79919	0.0026108	0.00939	Hit	B11R10C06

P29376	LTK	PV4651	134	6.61279	leukocyte tyrosine kinase (LTK), transcript variant 1	22828.5	41550	11626	30388.4	0.84644	0.0026294	0.03256	Hit	B25R15C18
O00506	STK25	NM_006374.2	135	6.59387	serine/threonine kinase 25 (STE20 homolog, yeast) (STK25)	43050.3	41090	12056	30306	0.55795	0.0026439	0.05271	Hit	B22R14C14
P08581	MET	PV3143	136	6.56918	met proto-oncogene (hepatocyte growth factor receptor) (MET); see catalog number for detailed information on wild-typ	0	39279	10859	30198.4	0.70529	0.0026629	0.0923	Hit	B13R16C02
P54760	EPHB4	PV3251	137	6.54627	ephrin receptor B4 (EPHB4)	0	39591	14245	30098.6	0.84661	0.0026808	0.03858	Hit	B27R16C01
P21802	FGFR2	NM_022972.1	138	6.54504	Fibroblast growth factor receptor 2	5698.67	41830	22955	30093.2	0.79919	0.0026817	0.00939	Hit	B11R10C05
P17612	PRKACA	NM_002730.1	139	6.54347	protein kinase, cAMP-dependent, catalytic, alpha (PRKACA), transcript variant 1	10989.5	39477	12917	30086.3	0.57273	0.002683	0.0681	Hit	B46R03C21
P15735	PHKG2	NM_000294.1	140	6.51755	phosphorylase kinase, gamma 2 (testis)	10065.1	38994	10511	29973.4	0.73702	0.0027034	0.06913	Hit	B25R02C20
P49841	GSK3B	NM_002093.2	141	6.49688	glycogen synthase kinase 3 beta (GSK3B)	31487.1	38587	8469	29883.3	0.91048	0.0027199	0.01647	Hit	B35R03C04
O75914	PAK3	PV3789	142	6.49594	p21 (CDKN1A)-activated kinase 3 (PAK3)	0	40947	19850	29879.2	0.89296	0.0027207	0.02316	Hit	B19R15C21
P78362	SRPK2	NM_182691.1	143	6.48316	SFRS protein kinase 2 (SRPK2), transcript variant 2	15706.5	38107	10405	29823.5	0.87047	0.0027309	0.01838	Hit	B29R20C20
P00519	ABL1	NM_005157.2	144	6.47732	v-abl Abelson murine leukemia viral oncogene homolog 1 (ABL1), transcript variant a; see catalog number for detailed i	36397.3	44479	26226	29798.1	0.74777	0.0027356	0.05463	Hit	B01R09C20
Q15759	MAPK11	PV3679	145	6.4045	mitogen-activated protein kinase 11 (MAPK11), transcript variant 2	0	37776	14029	29480.7	0.7315	0.0027954	0.07593	Hit	B19R16C01
P36888	FLT3	PV3967	146	6.36944	fms-related tyrosine kinase 3 (FLT3); see catalog number for detailed information on wild-type or point mutant status	0	40255	14982	29328	0.88693	0.0028249	0.02366	Hit	B35R15C18
Q9BZL6	PRKD2	PV3758	147	6.36142	protein kinase D2 (PRKD2), transcript variant 1	7730.89	43844	28274	29293	0.73594	0.0028317	0.02407	Hit	B28R15C15
P41743	PRKCI	PV3186	148	6.33831	protein kinase C, iota (PRKCI)	0	38742	9597	29192.3	0.63741	0.0028515	0.01855	Hit	B22R15C18
O75716	STK16	BC002618.1	149	6.32753	serine/threonine kinase 16 (STK16)	35013.7	41973	23541	29145.3	0.43538	0.0028608	0.08683	Hit	B40R10C01
Q9P289	RP6-213H19.1	NM_016542.2	150	6.32433	serine/threonine protein kinase MST4 (MST4), transcript variant 1; MASK	55567.9	38178	19202	29131.4	0.70246	0.0028635	0.07167	Hit	B29R05C17
Q9Y6M4	CSNK1G3	PV3838	151	6.30755	casein kinase 1, gamma 3 (CSNK1G3), transcript variant 2	16703.6	37406	10116	29058.3	0.95411	0.0028781	0.00884	Hit	B45R15C22
Q9NWZ3	IRAK4	PV3362	152	6.28471	interleukin-1 receptor-associated kinase 4 (IRAK4)	0	42284	27560	28958.7	0.76063	0.0028981	0.07004	Hit	B12R15C21
P00519	ABL1	P3049	153	6.24042	v-abl Abelson murine leukemia viral oncogene homolog 1 (ABL1), transcript variant a; see catalog number for detailed i	0	36411	16854	28765.7	0.81675	0.0029375	0.05432	Hit	B45R16C01
Q9Y6M4	CSNK1G3	PV3838	154	6.22472	casein kinase 1, gamma 3 (CSNK1G3), transcript variant 2	21533.6	39755	18504	28697.3	0.95411	0.0029517	0.00884	Hit	B45R15C21
P54760	EPHB4	PV3251	155	6.1794	ephrin receptor B4 (EPHB4)	0	37861	10172	28499.8	0.84661	0.0029932	0.03858	Hit	B27R16C02
P41743	PRKCI	PV3186	156	6.16487	protein kinase C, iota (PRKCI)	0	40396	16394	28436.5	0.63741	0.0030066	0.01855	Hit	B22R15C17
P36888	FLT3	PV3967	157	6.14795	fms-related tyrosine kinase 3 (FLT3); see catalog number for detailed information on wild-type or point mutant status	0	40658	19695	28362.7	0.88693	0.0030225	0.02366	Hit	B35R15C17
O00506	STK25	PV3657	158	6.13797	serine/threonine kinase 25 (STE20 homolog, yeast) (STK25)	7118.4	39825	15895	28319.2	0.81228	0.0030318	0.05432	Hit	B34R15C16
Q9BZL6	PRKD2	PV3758	159	6.13641	protein kinase D2 (PRKD2), transcript variant 1	8235.75	42112	21592	28312.4	0.73594	0.0030333	0.02407	Hit	B28R15C16
O15530	PDPK1	P3001	160	6.07859	3-phosphoinositide dependent protein kinase-1 (PDPK1), transcript variant 1	0	40652	19602	28060.4	0.62473	0.0030886	0.10284	Hit	B14R15C16
P15531	NME1	NM_198175.1	161	6.07319	non-metastatic cells 1, protein (NM23A) expressed in (NME1), transcript variant 1	85960.2	38129	11873	28036.9	0.60064	0.0030938	0.02555	Hit	B22R08C20
Q00535	CDK5	PV4676	162	6.06759	cyclin-dependent kinase 5 (CDK5) and p25: CDK5 and p25 sequences are seperated by -- (in protein list file).	8562.82	37104	9002	28012.5	0.87352	0.0030993	0.02846	Hit	B39R16C06
O94806	PRKD3	PV3692	163	6.0446	Serine/threonine-protein kinase D3	8585.09	38745	17681	27912.3	0.8974	0.0031218	0.02079	Hit	B27R15C21
P49137	MAPKAPK2	PV3317	164	6.0311	MAP kinase-activated protein kinase 2	851.22	38624	15143	27853.5	0.5589	0.0031351	0.03727	Hit	B22R16C03
P54763	EPHB2	PV3625	165	6.02515	Ephrin type-B receptor 2	12611.5	36172	14183	27827.6	0.5682	0.003141	0.12727	Hit	B30R15C21
P49761	CLK3	BC002555.1	166	6.00562	CDC-like kinase 3 (CLK3)	15346	38296	20899	27742.5	0.8946	0.0031605	0.02852	Hit	B21R14C05
O96013	PAK4	BC002921.2	167	5.97452	p21(CDKN1A)-activated kinase 4 (PAK4)	126657	36297	13269	27606.9	0.8509	0.003192	0.04083	Hit	B37R19C19
P22694	PRKACB	NM_002731.2	169	5.91567	protein kinase, cAMP-dependent, catalytic, beta (PRKACB), transcript variant 2	30765	48879	35925	27350.5	0.81687	0.0032527	0.02115	Hit	B36R21C07
P17612	PRKACA	NM_002730.1	170	5.90913	protein kinase, cAMP-dependent, catalytic, alpha (PRKACA), transcript variant 1	8901.25	35833	8116	27322	0.57273	0.0032596	0.0681	Hit	B46R03C22
P08581	MET	PV3968	171	5.85969	met proto-oncogene (hepatocyte growth factor receptor) (MET); see catalog number for detailed information on wild-typ	0	45596	30616	27106.5	0.45899	0.0033122	0.01732	Hit	B32R15C13
O94806	PRKD3	PV3692	172	5.85903	Serine/threonine-protein kinase D3	11917	37031	12922	27103.6	0.8974	0.0033129	0.02079	Hit	B27R15C22
P15531	NME1	NM_198175.1	173	5.84485	non-metastatic cells 1, protein (NM23A) expressed in (NME1), transcript variant 1	93064	40454	21407	27041.9	0.60064	0.0033282	0.02555	Hit	B22R08C19
Q13627	DYRK1A	PV3785	174	5.82884	Dual specificity tyrosine-phosphorylation-regulated kinase 1A	20889.8	35942	16945	26972.1	0.93169	0.0033456	0.01394	Hit	B37R16C01
Q00535	CDK5	PV4676	175	5.81402	cyclin-dependent kinase 5 (CDK5) and p25: CDK5 and p25 sequences are seperated by -- (in protein list file).	9527.37	37867	15501	26907.5	0.87352	0.0033619	0.02846	Hit	B39R16C05
P49761	CLK3	BC002555.1	176	5.75393	CDC-like kinase 3 (CLK3)	16023.6	35710	8563	26645.6	0.8946	0.003429	0.02852	Hit	B21R14C06
P00519	ABL1	P3049	177	5.75213	v-abl Abelson murine leukemia viral oncogene homolog 1 (ABL1), transcript variant a; see catalog number for detailed i	0	34615	12117	26637.8	0.81675	0.003431	0.05432	Hit	B45R16C02
P22694	PRKACB	NM_002731.2	178	5.73068	protein kinase, cAMP-dependent, catalytic, beta (PRKACB), transcript variant 2	31089	47524	30870	26544.3	0.81687	0.0034555	0.02115	Hit	B36R21C08
Q15759	MAPK11	PV3679	179	5.71506	mitogen-activated protein kinase 11 (MAPK11), transcript variant 2	0	34892	8883	26476.2	0.7315	0.0034735	0.07593	Hit	B19R16C02
P08581	MET	PV3968	180	5.70915	met proto-oncogene (hepatocyte growth factor receptor) (MET); see catalog number for detailed information on wild-typ	0	46919	32225	26450.5	0.45899	0.0034803	0.01732	Hit	B32R15C14
P15735	PHKG2	PV4555	181	5.70882	phosphorylase kinase, gamma 2 (testis)	9004.68	46800	25019	26449	0.66959	0.0034807	0.09465	Hit	B39R15C19
Q13627	DYRK1A	PV3785	182	5.70804	Dual specificity tyrosine-phosphorylation-regulated kinase 1A	17214.8	35800	12829	26445.6	0.93169	0.0034816	0.01394	Hit	B37R16C02
Q05397	PTK2	PV3832	183	5.70447	Focal adhesion kinase 1	7486.34	35390	12702	26430.1	0.55301	0.0034858	0.10575	Hit	B18R15C17
P49137	MAPKAPK2	PV3317	184	5.70284	MAP kinase-activated protein kinase 2	923.56	36349	8731	26423	0.5589	0.0034877	0.03727	Hit	B22R16C04
Q9P289	RP6-213H19.1	NM_016542.2	185	5.67947	serine/threonine protein kinase MST4 (MST4), transcript variant 1	32556.6	34537	10540	26321.1	0.70246	0.003515	0.07167	Hit	B29R05C18
Q99683	MAP3K5	PV3809	186	5.65751	mitogen-activated protein kinase kinase kinase 5 (MAP3K5)	1680.73	34745	14831	26225.4	0.7299	0.003541	0.07817	Hit	B10R15C17
Q8CIP4	MARK4	PV3851	187	5.6508	MAP/microtubule affinity-regulating kinase 4	11096.8	38721	27224	26196.2	0.87076	0.003549	0.00592	Hit	B20R15C17
O96013	PAK4	BC002921.2	188	5.61895	p21(CDKN1A)-activated kinase 4 (PAK4)	320.06	33506	7777	26057.4	0.8509	0.0035873	0.04083	Hit	B37R19C20
P78368	CSNK1G2	PV3499	189	5.60987	casein kinase 1, gamma 2 (CSNK1G2)	0	39533	28247	26017.8	0.60616	0.0035983	0.07656	Hit	B16R15C17
Q8CIP4	MARK4	PV3851	190	5.6007	MAP/microtubule affinity-regulating kinase 4	11926.2	39836	28774	25977.9	0.87076	0.0036095	0.00592	Hit	B20R15C18
P78362	SRPK2	NM_182692.1	192	5.56529	Serine/threonine-protein kinase SRPK2	17652.2	39135	28950	25823.5	0.54133	0.0036533	0.12906	Hit	B04R18C07
O75716	STK16	BC002618.1	193	5.55378	serine/threonine kinase 16 (STK16)	35080.8	40484	18483	25773.4	0.43538	0.0036677	0.08683	Hit	B40R10C02
Q9UHD2	TBK1	PV3504	194	5.52612	TANK-binding kinase 1 (TBK1)	0	34551	14040	25652.8	0.57786	0.0037026	0.1303	Hit	B37R15C17
Q13177	PAK2	PV4565	195	5.51591	p21 (CDKN1A)-activated kinase 2 (PAK2)	8575.33	35941	11552	25608.3	0.60806	0.0037156	0.05833	Hit	B11R15C14
Q9H0K1	SNF1LK2	PV4792	196	5.50293	SNF1-like kinase 2 (SNF1LK2)	10766.8	35768	15718	25551.8	0.60065	0.0037323	0.10827	Hit	B25R16C04
Q14289	PTK2B	BC036651.2	198	5.48117	PTK2B protein tyrosine kinase 2 beta (PTK2B)	19729.9	32724	12073	25457	0.61629	0.0037605	0.11986	Hit	B45R02C11
O95819	MAP4K4	PV3687	199	5.46423	Mitogen-activated protein kinase kinase kinase kinase 4	9715.46	40219	25180	25383.1	0.84993	0.0037826	0.01821	Hit	B44R15C18
P06493	CDC2	PV3292	200	5.4586	cell division cycle 2, G1 to S and G2 to M (CDC2), transcript variant 1	0	34954	14139	25358.6	0.88883	0.00379	0.02229	Hit	B27R15C17
Q00535	CDK5	PV3000	201	5.44975	cyclin-dependent kinase 5 (CDK5) and p25: CDK5 and p25 sequences are seperated by -- (in protein list file).	0	44082	33117	25320	0.79406	0.0038017	0.01753	Hit	B48R15C16
P29317	EPHA2	PV3688	202	5.40683	Ephrin type-A receptor 2	6351.58	33919	12927	25133	0.90613	0.0038591	0.01911	Hit	B26R15C22

P17252	PRKCA	P2227	206	5.31971	protein kinase C, alpha (PRKCA); see catalog number for detailed information on wild-type or point mutant status	0	36681	21750	24753.3	0.71581	0.0039798	0.05635	Hit	B01R15C21
O95819	MAP4K4	PV3687	207	5.31612	Mitogen-activated protein kinase kinase kinase kinase 4	12773.2	40671	28220	24737.7	0.84993	0.0039849	0.01821	Hit	B44R15C17
Q16512	PKN1	PV3790	208	5.31108	Serine/threonine-protein kinase N1	5281.03	37855	26100	24715.7	0.76646	0.0039921	0.03717	Hit	B20R15C19
Q00535	CDK5	PV3000	209	5.3075	cyclin-dependent kinase 5 (CDK5) and p25: CDK5 and p25 sequences are seperated by -- (in protein list file).	0	39532	27751	24700.1	0.79406	0.0039972	0.01753	Hit	B48R15C15
P06493	CDC2	PV3292	210	5.27802	cell division cycle 2, G1 to S and G2 to M (CDC2), transcript variant 1	0	33960	11474	24571.6	0.88883	0.0040396	0.02229	Hit	B27R15C18
P29317	EPHA2	PV3688	212	5.25301	Ephrin type-A receptor 2	5948.88	34547	14579	24462.7	0.90613	0.0040761	0.01911	Hit	B26R15C21
A8K040	CSNK1G3	BC047567.1	214	5.23761	casein kinase 1, gamma 3 (CSNK1G3)	6545.67	37939	20934	24395.5	0.56574	0.0040988	0.09761	Hit	B36R04C07
Q05397	PTK2	BC035404.2	215	5.18017	PTK2 protein tyrosine kinase 2 (PTK2)	24933.7	34676	20279	24145.2	0.82265	0.0041853	0.04407	Hit	B03R02C11
P51451	BLK	NM_001715.2	216	5.14998	B lymphoid tyrosine kinase (BLK)	13905.2	33041	18328	24013.7	0.85789	0.0042318	0.03468	Hit	B12R19C14
O15264	MAPK13	PV3656	217	5.14995	mitogen-activated protein kinase 13 (MAPK13)	0	31715	11568	24013.6	0.74675	0.0042318	0.06662	Hit	B35R16C01
P37173	TGFBR2	NM_003242.2	219	5.10036	transforming growth factor, beta receptor II (70/80kDa) (TGFBR2), transcript variant 2	37499.4	32500	12160	23797.4	0.47259	0.00431	0.16173	Hit	B26R10C09
Q9Y6E0	STK24	PV3650	220	5.08957	serine/threonine kinase 24 (STE20 homolog, yeast) (STK24), transcript variant 2	6893.05	37728	27491	23750.4	0.74705	0.0043273	0.02727	Hit	B16R15C16
Q13177	PAK2	PV4565	221	5.05039	p21 (CDKN1A)-activated kinase 2 (PAK2)	10217	33879	12839	23579.7	0.60806	0.0043909	0.05833	Hit	B11R15C13
P51617	IRAK1	PV4403	222	5.04937	Interleukin-1 receptor-associated kinase 1	6469.74	32820	12782	23575.2	0.86542	0.0043926	0.02856	Hit	B39R16C01
P16234	PDGFRA,	PV4203	223	5.04035	platelet-derived growth factor receptor, alpha polypeptide (PDGFRA); see catalog number for detailed information on w	4164.72	32335	14279	23535.9	0.75243	0.0044075	0.06565	Hit	B19R15C13
Q06418	TYRO3	PV3828	224	5.03894	TYRO3 protein tyrosine kinase (TYRO3)	5907.89	32461	7198	23529.8	0.90137	0.0044098	0.0224	Hit	B34R15C18
Q99683	MAP3K5	PV3809	225	5.0271	mitogen-activated protein kinase kinase kinase 5 (MAP3K5)	1548.11	31380	8451	23478.2	0.7299	0.0044294	0.07817	Hit	B10R15C18
P07949	RET	PV3819	227	5.02162	ret proto-oncogene (RET), transcript variant 4	10595.6	31223	12359	23454.3	0.90542	0.0044386	0.0068	Hit	B25R15C22
Q16512	PKN1	PV3790	228	5.02058	Serine/threonine-protein kinase N1	4244.73	34263	17522	23449.8	0.76646	0.0044403	0.03717	Hit	B20R15C20
P78368	CSNK1G2	PV3499	231	4.99665	casein kinase 1, gamma 2 (CSNK1G2)	0	35653	19681	23345.5	0.60616	0.0044806	0.07656	Hit	B16R15C18
Q86V86	PIM3	NM_001001852.2	232	4.98536	pim-3 oncogene (PIM3)	25868.5	34009	21418	23296.3	0.78469	0.0044997	0.0291	Hit	B24R08C13
O00506	STK25	BC007852.1	233	4.97183	Serine/threonine-protein kinase 25	59486.4	30741	11483	23237.3	0.52659	0.0045229	0.06166	Hit	B46R10C01
P54763	EPHB2	PV3625	234	4.97076	Ephrin type-B receptor 2	13069.7	30966	10354	23232.7	0.5682	0.0045247	0.12727	Hit	B30R15C22
P07949	RET	PV3819	235	4.97007	ret proto-oncogene (RET), transcript variant 4	8895.59	32021	15987	23229.7	0.90542	0.0045259	0.0068	Hit	B25R15C21
P15735	PHKG2	PV4555	236	4.9474	phosphorylase kinase, gamma 2 (testis)	3781.05	37533	21832	23130.9	0.66959	0.0045651	0.09465	Hit	B39R15C20
Q15303	ERBB4	NM_005235.1	237	4.9448	Receptor tyrosine-protein kinase erbB-4	81063.4	29781	10359	23119.5	0.62541	0.0045697	0.10366	Hit	B41R10C01
P29218	IMPA1	NM_005536.2	238	4.92471	inositol(myo)-1(or 4)-monophosphatase 1 (IMPA1)	22975.7	32088	16063	23032	0.74059	0.0046049	0.05491	Hit	B08R17C07
O43781	DYRK3	PV3837	239	4.91534	dual-specificity tyrosine-(Y)-phosphorylation regulated kinase 3 (DYRK3), transcript variant 2	30692.1	30933	12845	22991.1	0.72026	0.0046215	0.07251	Hit	B41R15C17
P05129	PRKCG	P2228	241	4.90917	protein kinase C, gamma (PRKCG)	0	34569	20576	22964.2	0.74487	0.0046324	0.04406	Hit	B01R15C15
P51451	BLK	NM_001715.2	242	4.88621	B lymphoid tyrosine kinase (BLK)	13294.3	32727	20916	22864.2	0.85789	0.0046736	0.03468	Hit	B12R19C13
P17252	PRKCA	P2227	243	4.88441	protein kinase C, alpha (PRKCA); see catalog number for detailed information on wild-type or point mutant status	0	33746	14542	22856.3	0.71581	0.0046768	0.05635	Hit	B01R15C22
Q9Y6E0	STK24	PV3650	244	4.88334	serine/threonine kinase 24 (STE20 homolog, yeast) (STK24), transcript variant 2	6530.05	36330	25270	22851.7	0.74705	0.0046788	0.02727	Hit	B16R15C15
Q06418	TYRO3	PV3828	245	4.87058	TYRO3 protein tyrosine kinase (TYRO3)	4692.55	34747	14186	22796.1	0.90137	0.0047019	0.0224	Hit	B34R15C17
P78362	SRPK2	BC068547.1	246	4.86293	Serine/threonine-protein kinase SRPK2	5005.85	32142	15870	22762.7	0.66339	0.0047159	0.09575	Hit	B03R18C13
Q05397	PTK2	PV3832	247	4.86055	Focal adhesion kinase 1	7295.08	31546	8680	22752.4	0.55301	0.0047202	0.10575	Hit	B18R15C18
Q05397	PTK2	BC035404.2	248	4.84529	PTK2 protein tyrosine kinase 2 (PTK2)	21496.9	31962	12242	22685.9	0.82265	0.0047483	0.04407	Hit	B03R02C12
P51617	IRAK1	PV4403	249	4.83518	Interleukin-1 receptor-associated kinase 1	5476.25	30952	9259	22641.8	0.86542	0.004767	0.02856	Hit	B39R16C02
Q9H2K8	TAOK3	PV3652	250	4.81152	TAO kinase 3 (TAOK3)	7515.61	31574	13797	22538.7	0.74584	0.0048113	0.06873	Hit	B23R15C13
P51813	BMX	NM_001721.2	252	4.79789	BMX non-receptor tyrosine kinase (BMX), transcript variant 2	29012.5	33065	20884	22479.3	0.8899	0.0048371	0.00575	Hit	B08R14C11
P53355	DAPK1	PV3969	253	4.79777	Death-associated protein kinase 1	18121.5	31459	13486	22478.8	0.73257	0.0048373	0.00102	Hit	B17R15C17
P53355	DAPK1	PV3969	254	4.79035	Death-associated protein kinase 1	21056.2	31370	12045	22446.5	0.73257	0.0048514	0.00102	Hit	B17R15C18
Q86V86	PIM3	NM_001001852.2	255	4.7698	pim-3 oncogene (PIM3)	25298.6	32212	14426	22356.9	0.78469	0.0048909	0.0291	Hit	B24R08C14
P51813	BMX	NM_001721.2	256	4.75613	BMX non-receptor tyrosine kinase (BMX), transcript variant 2	37521.5	33906	22175	22297.3	0.8899	0.0049174	0.00575	Hit	B08R14C12
P51451	BLK	PV3683	257	4.71528	B lymphoid tyrosine kinase (BLK)	0	29487	10757	22119.3	0.65152	0.0049979	0.10206	Hit	B31R16C01
P54756	EPHA5	PV3840	258	4.69487	Ephrin receptor A5 (EPHA5), transcript variant 1	8210.38	35522	19887	22030.3	0.93967	0.0050388	0.00311	Hit	B39R15C21
P54756	EPHA5	PV3840	260	4.67271	Ephrin receptor A5 (EPHA5), transcript variant 1	2261.88	32137	15337	21933.8	0.93967	0.0050839	0.00311	Hit	B39R15C22
Q9H0K1	SNF1LK2	PV4792	261	4.66899	SNF1-like kinase 2 (SNF1LK2)	12736.9	32389	18922	21917.6	0.60065	0.0050915	0.10827	Hit	B25R16C03
O15264	MAPK13	PV3656	262	4.65415	mitogen-activated protein kinase 13 (MAPK13)	0	29787	8103	21852.9	0.74675	0.0051221	0.06662	Hit	B35R16C02
P48730	CSNK1D	NM_001893.3	263	4.64936	casein kinase 1, delta (CSNK1D), transcript variant 1	11569.1	29306	9874	21832	0.75985	0.005132	0.06594	Hit	B10R09C21
P05129	PRKCG	P2228	264	4.59072	protein kinase C, gamma (PRKCG)	0	32957	14723	21576.5	0.74487	0.0052559	0.04406	Hit	B01R15C16
P78362	SRPK2	NM_182692.1	265	4.57422	Serine/threonine-protein kinase SRPK2	17078.5	33765	17388	21504.6	0.54133	0.0052916	0.12906	Hit	B04R18C08
Q14289	PTK2B	BC036651.2	266	4.56833	PTK2B protein tyrosine kinase 2 beta (PTK2B)	15989.9	28567	7433	21478.9	0.61629	0.0053044	0.11986	Hit	B45R02C12
P16234	PDGFRA,	PV4203	267	4.5612	platelet-derived growth factor receptor, alpha polypeptide (PDGFRA); see catalog number for detailed information on w	4605.78	32231	15369	21447.8	0.75243	0.00532	0.06565	Hit	B19R15C14
Q9UHD2	TBK1	PV3504	269	4.5329	TANK-binding kinase 1 (TBK1)	0	30460	10833	21324.5	0.57786	0.0053826	0.1303	Hit	B37R15C18
P29218	IMPA1	NM_005536.2	270	4.52964	inositol(myo)-1(or 4)-monophosphatase 1 (IMPA1)	34024.2	29846	11074	21310.3	0.74059	0.0053898	0.05491	Hit	B08R17C08
O00506	STK25	BC007852.1	271	4.52629	Serine/threonine-protein kinase 25	54077.7	29551	8475	21295.7	0.52659	0.0053973	0.06166	Hit	B46R10C02
A8K040	CSNK1G3	BC047567.1	272	4.51475	casein kinase 1, gamma 3 (CSNK1G3)	7802.5	34530	15498	21245.4	0.56574	0.0054233	0.09761	Hit	B36R04C08
P51956	NEK3	PV3821	276	4.47677	NIMA (never in mitosis gene a)-related kinase 3 (NEK3), transcript variant 1	39713.1	29702	11826	21079.9	0.85674	0.0055099	0.02077	Hit	B04R15C22
O14508	SOCS2	NM_003877.3	277	4.47482	suppressor of cytokine signaling 2 (SOCS2)	28589.3	28642	13186	21071.4	0.81643	0.0055144	0.05174	Hit	B13R02C15
O43781	DYRK3	PV3837	279	4.40075	dual-specificity tyrosine-(Y)-phosphorylation regulated kinase 3 (DYRK3), transcript variant 2	31241.9	28121	8716	20748.6	0.72026	0.0056898	0.07251	Hit	B41R15C18
Q8N568	DCAMKL2	PV4297	280	4.39753	doublecortin and CaM kinase-like 2 (DCAMKL2)	6389.54	32261	14627	20734.6	0.41839	0.0056976	0.17555	Hit	B23R15C16
O00238	BMPR1B	NM_001203.1	282	4.35717	bone morphogenetic protein receptor, type IB (BMPR1B)	5316.16	29208	7682	20558.7	0.84891	0.0057968	0.03576	Hit	B10R04C12
P00533	EGFR,T790M	PV4803	283	4.34295	epidermal growth factor receptor (erythroblastic leukemia viral (v-erb-b) oncogene homolog, avian) (EGFR); see catalog	19259.7	27180	11414	20496.7	0.61647	0.0058324	0.11787	Hit	B45R16C05
P43403	ZAP70	NM_207519.1	284	4.33752	zeta-chain (TCR) associated protein kinase 70kDa (ZAP70), transcript variant 2	48628	28808	11315	20473	0.6638	0.0058461	0.02421	Hit	B11R08C13
P51956	NEK3	PV3821	285	4.33672	NIMA (never in mitosis gene a)-related kinase 3 (NEK3), transcript variant 1	39773.6	32865	20941	20469.6	0.85674	0.0058481	0.02077	Hit	B04R15C21
Q9H2K8	TAOK3	PV3652	287	4.33208	TAO kinase 3 (TAOK3)	3460.33	31195	13609	20449.4	0.74584	0.0058599	0.06873	Hit	B23R15C14

P43403	ZAP70	BC039039.1	288	4.31899	zeta-chain (TCR) associated protein kinase 70kDa (ZAP70)	70017.6	30331	15977	20392.3	0.56553	0.0058932	0.10916	Hit	B14R09C11
O60285	NUAK1	NM_014840.2	289	4.30081	NUAK family, SNF1-like kinase, 1 (NUAK1)	16474.6	28235	12302	20313.1	0.76268	0.0059399	0.05278	Hit	B33R09C15
P05771	PRKCB1	NM_002738.5	291	4.2988	protein kinase C, beta 1 (PRKCB1), transcript variant 2	10660.7	37667	29720	20304.3	0.7205	0.0059451	0.04669	Hit	B01R05C17
P29320	EPHA3	PV3359	292	4.28962	Ephrin receptor A3 (EPHA3), transcript variant 1	0	31236	14592	20264.3	0.92502	0.0059689	0.00779	Hit	B23R15C17
P00519	ABL1	PV3864	293	4.2756	v-abl Abelson murine leukemia viral oncogene homolog 1 (ABL1), transcript variant a; see catalog number for detailed i	0	28583	14242	20203.2	0.69321	0.0060056	0.06279	Hit	B29R16C01
P29320	EPHA3	PV3359	296	4.23866	Ephrin receptor A3 (EPHA3), transcript variant 1	0	30037	14044	20042.3	0.92502	0.0061038	0.00779	Hit	B23R15C18
Q15303	ERBB4	NM_005235.1	297	4.2202	Receptor tyrosine-protein kinase erbB-4	87897.3	26912	6528	19961.8	0.62541	0.0061538	0.10366	Hit	B41R10C02
P48730	CSNK1D	NM_001893.3	298	4.20299	casein kinase 1, delta (CSNK1D), transcript variant 1	7863	26573	5735	19886.8	0.75985	0.006201	0.06594	Hit	B10R09C22
P78362	SRPK2	BC068547.1	299	4.20047	Serine/threonine-protein kinase SRPK2	6862.79	28385	8990	19875.8	0.66339	0.006208	0.09575	Hit	B03R18C14
P43403	ZAP70	NM_207519.1	300	4.17935	zeta-chain (TCR) associated protein kinase 70kDa (ZAP70), transcript variant 2	49560.9	27170	7843	19783.8	0.6638	0.0062667	0.02421	Hit	B11R08C14
P49841	GSK3B	BC000251.1	301	4.16159	Glycogen synthase kinase-3 beta	20132.7	28629	16250	19706.4	0.63129	0.0063167	0.08499	Hit	B08R10C09
P15531	NME1	NM_000269.1	302	4.15071	non-metastatic cells 1, protein (NM23A) expressed in (NME1), transcript variant 2	48360.3	27492	7193	19658.9	0.80089	0.0063477	0.0441	Hit	B30R14C12
O14508	SOCS2	NM_003877.3	305	4.13349	suppressor of cytokine signaling 2 (SOCS2)	23470	27419	9475	19583.9	0.81643	0.0063971	0.05174	Hit	B13R02C16
O00238	BMPR1B	NM_001203.1	306	4.12445	bone morphogenetic protein receptor, type IB (BMPR1B)	6750.59	29190	12601	19544.5	0.84891	0.0064233	0.03576	Hit	B10R04C11
P11309	PIM1	NM_002648.1	308	4.1083	pim-1 oncogene (PIM1)	27726.1	31216	17078	19474.1	0.69867	0.0064705	0.04353	Hit	B36R10C09
Q92630	DYRK2	NM_003583.2	309	4.07309	dual-specificity tyrosine-(Y)-phosphorylation regulated kinase 2 (DYRK2), transcript variant 1	2101.87	30612	19788	19320.7	0.72001	0.0065753	0.05564	Hit	B08R14C13
P24941	CDK2	PV3267	310	4.04296	cyclin-dependent kinase 2 (CDK2), transcript variant 1	0	26512	6940	19189.4	0.53937	0.0066669	0.03901	Hit	B46R15C18
P49674	CSNK1E	PV3500	311	4.03868	casein kinase 1, epsilon (CSNK1E), transcript variant 2	0	25983	7557	19170.7	0.74622	0.0066801	0.06147	Hit	B30R16C02
P51451	BLK	PV3683	312	4.03199	B lymphoid tyrosine kinase (BLK)	0	26878	8485	19141.6	0.65152	0.0067007	0.10206	Hit	B31R16C02
P53778	MAPK12	PV3654	313	4.01948	mitogen-activated protein kinase 12 (MAPK12)	0	32807	20807	19087.1	0.7409	0.0067397	0.06556	Hit	B19R15C16
Q02156	PRKCE	P2282	314	4.00883	protein kinase C, epsilon (PRKCE)	0	28781	16722	19040.7	0.88367	0.006773	0.00231	Hit	B08R15C17
P05771	PRKCB1	NM_002738.5	316	4.00098	protein kinase C, beta 1 (PRKCB1), transcript variant 2	8830.48	35810	26295	19006.4	0.7205	0.0067978	0.04669	Hit	B01R05C18
Q02156	PRKCE	P2282	318	3.99459	protein kinase C, epsilon (PRKCE)	0	27746	11735	18978.6	0.88367	0.0068181	0.00231	Hit	B08R15C18
P37173	TGFBR2	NM_003242.2	320	3.97953	transforming growth factor, beta receptor II (70/80kDa) (TGFBR2), transcript variant 2	30532.8	27237	8190	18913	0.47259	0.0068662	0.16173	Hit	B26R10C10
O60285	NUAK1	NM_014840.2	321	3.96538	NUAK family, SNF1-like kinase, 1 (NUAK1)	14497.2	28964	11952	18851.3	0.76268	0.0069119	0.05278	Hit	B33R09C16
Q9Z0Z0	PRKG1	PV4340	323	3.88863	cGMP-dependent protein kinase 1, beta isozyme	8984.81	27628	12665	18516.8	0.70802	0.0071678	0.05959	Hit	B14R16C05
P00519	ABL1	PV3864	324	3.88141	v-abl Abelson murine leukemia viral oncogene homolog 1 (ABL1), transcript variant a; see catalog number for detailed i	0	27516	10865	18485.4	0.69321	0.0071926	0.06279	Hit	B29R16C02
P15735	PHKG2	BC002541.1	325	3.88088	Phosphorylase b kinase gamma catalytic chain, testis/liver isoform	3419.2	32134	21923	18483.1	0.74806	0.0071944	0.01397	Hit	B48R09C20
P15531	NME1	NM_000269.1	326	3.87785	non-metastatic cells 1, protein (NM23A) expressed in (NME1), transcript variant 2	38878.7	27022	10361	18469.8	0.80089	0.0072049	0.0441	Hit	B30R14C11
P11309	PIM1	NM_002648.1	329	3.84143	pim-1 oncogene (PIM1)	28119.1	29645	15390	18311.2	0.69867	0.0073323	0.04353	Hit	B36R10C10
P24941	CDK2	PV3267	331	3.80653	cyclin-dependent kinase 2 (CDK2), transcript variant 1	0	26209	10089	18159.1	0.53937	0.0074576	0.03901	Hit	B46R15C17
P15735	PHKG2	BC002541.1	332	3.79789	Phosphorylase b kinase gamma catalytic chain, testis/liver isoform	4748.24	30926	20466	18121.4	0.74806	0.0074891	0.01397	Hit	B48R09C19
P22455	FGFR4	P3054	335	3.78313	fibroblast growth factor receptor 4 (FGFR4), transcript variant 1	0	25615	10104	18057.1	0.72806	0.0075434	0.06908	Hit	B47R16C01
O94921	PFTK1	NM_012395.1	336	3.77253	Serine/threonine-protein kinase PFTAIRE-1	14978.5	25622	10923	18010.9	0.81738	0.0075827	0.04408	Hit	B10R10C03
Q92630	DYRK2	NM_003583.2	337	3.73747	dual-specificity tyrosine-(Y)-phosphorylation regulated kinase 2 (DYRK2), transcript variant 1	2049.18	27155	14123	17858.1	0.72001	0.0077152	0.05564	Hit	B08R14C14
Q9H0K1	SNF1LK2	NM_015191.1	338	3.72016	SNF1-like kinase 2 (SNF1LK2)	10390.6	26845	14150	17782.7	0.72586	0.0077818	0.06112	Hit	B33R07C03
O75716	STK16	BC053998.1	339	3.70258	serine/threonine kinase 16 (STK16)	23401.6	24797	11186	17706.1	0.43424	0.0078504	0.06766	Hit	B17R19C13
P49674	CSNK1E	PV3500	342	3.67216	casein kinase 1, epsilon (CSNK1E), transcript variant 2	0	24741	10022	17573.5	0.74622	0.0079712	0.06147	Hit	B30R16C01
P49841	GSK3B	BC000251.1	343	3.64887	Glycogen synthase kinase-3 beta	15267.4	25754	10652	17472	0.63129	0.0080657	0.08499	Hit	B08R10C10
P43403	ZAP70	BC039039.1	344	3.64835	zeta-chain (TCR) associated protein kinase 70kDa (ZAP70)	70688.9	26877	10093	17469.7	0.56553	0.0080678	0.10916	Hit	B14R09C12
P53778	MAPK12	PV3654	346	3.6314	mitogen-activated protein kinase 12 (MAPK12)	0	29873	17952	17395.9	0.7409	0.0081376	0.06556	Hit	B19R15C15
P43405	SYK	PV3857	347	3.62522	spleen tyrosine kinase (SYK)	6498.67	24499	10336	17368.9	0.87678	0.0081633	0.01637	Hit	B30R15C17
P00533	EGFR,T790M	PV4803	348	3.61927	epidermal growth factor receptor (erythroblastic leukemia viral (v-erb-b) oncogene homolog, avian) (EGFR); see catalog	16134.6	23884	6306	17343	0.61647	0.0081881	0.11787	Hit	B45R16C06
P49760	CLK2	PV4201	350	3.60322	CDC-like kinase 2 (CLK2), transcript variant 1	26625.7	25120	13073	17273	0.50402	0.0082557	0.14633	Hit	B09R16C01
O00238	BMPR1B	NM_001203.1	351	3.59746	bone morphogenetic protein receptor, type IB (BMPR1B)	50135	25608	10658	17248	0.63972	0.0082801	0.06297	Hit	B43R10C09
Q8IU85	CAMK1D	PV3663	353	3.57287	calcium/calmodulin-dependent protein kinase ID (CAMK1D), transcript variant 1	0	35520	24209	17140.8	0.51623	0.0083857	0.00011	Hit	B40R15C15
Q8IU85	CAMK1D	PV3663	354	3.57224	calcium/calmodulin-dependent protein kinase ID (CAMK1D), transcript variant 1	0	32354	20451	17138	0.51623	0.0083884	0.00011	Hit	B40R15C16
P22392	NME2	NM_002512.1	355	3.56937	non-metastatic cells 2, protein (NM23B) expressed in (NME2), transcript variant 1	36681.9	28498	21034	17125.5	0.80002	0.0084009	0.00928	Hit	B20R15C03
Q9Z0Z0	PRKG1	PV4340	356	3.54503	cGMP-dependent protein kinase 1, beta isozyme	7808.35	25484	8558	17019.5	0.70802	0.0085077	0.05959	Hit	B14R16C06
P43405	SYK	PV3857	357	3.53399	spleen tyrosine kinase (SYK)	6496.26	24029	8548	16971.4	0.87678	0.0085568	0.01637	Hit	B30R15C18
O94921	PFTK1	NM_012395.1	359	3.52267	Serine/threonine-protein kinase PFTAIRE-1	13967.6	23726	7725	16922	0.81738	0.0086076	0.04408	Hit	B10R10C04
P22392	NME2	NM_002512.1	360	3.51814	non-metastatic cells 2, protein (NM23B) expressed in (NME2), transcript variant 1	37270.3	29131	23558	16902.3	0.80002	0.0086281	0.00928	Hit	B20R15C04
O15547	P2RXL1	NM_005446.2	364	3.47979	purinergic receptor P2X-like 1, orphan receptor (P2RXL1)	15919.2	35063	59002	16735.2	0.5615	0.0088043	0.12134	Hit	B05R09C01
O75116	ROCK2	PV3759	365	3.47902	Rho-associated protein kinase 2	7248.24	23776	10236	16731.8	0.93132	0.0088079	0.00214	Hit	B23R16C01
O75116	ROCK2	PV3759	366	3.46744	Rho-associated protein kinase 2	7742	26293	10504	16681.4	0.93132	0.0088622	0.00214	Hit	B23R16C02
P07332	FES	PV3354	367	3.43716	feline sarcoma oncogene (FES)	0	30190	20779	16549.4	0.79096	0.0090065	0.00452	Hit	B36R15C18
P07332	FES	PV3354	369	3.41298	feline sarcoma oncogene (FES)	0	30718	21991	16444	0.79096	0.0091244	0.00452	Hit	B36R15C17
Q9Y664	KPTN	BC009249.1	370	3.41152	kaptin (actin binding protein) (KPTN)	6809.74	33067	52315	16437.7	0.49938	0.0091315	0.14076	Hit	B39R14C17
P22455	FGFR4	P3054	371	3.39717	fibroblast growth factor receptor 4 (FGFR4), transcript variant 1	0	24451	8420	16375.1	0.72806	0.0092027	0.06908	Hit	B47R16C02
Q9H0K1	SNF1LK2	NM_015191.1	373	3.38206	SNF1-like kinase 2 (SNF1LK2)	8801.54	24555	7964	16309.3	0.72586	0.0092784	0.06112	Hit	B33R07C04
P33981	TTK	PV3792	374	3.38014	TTK protein kinase (TTK)	27230.7	28089	16846	16300.9	0.9102	0.0092881	0.01833	Hit	B45R15C20
Q8N568	DCAMKL2	PV4297	376	3.34672	doublecortin and CaM kinase-like 2 (DCAMKL2)	6863.78	28181	14327	16155.3	0.41839	0.0094594	0.17555	Hit	B23R15C15
O75716	STK16	BC053998.1	377	3.33154	serine/threonine kinase 16 (STK16)	24322.8	22855	8012	16089.1	0.43424	0.0095387	0.06766	Hit	B17R19C14
P54760	EPHB4	NM_004444.2	378	3.32952	ephrin receptor B4 (EPHB4)	19305.5	25880	11083	16080.3	0.81123	0.0095494	0.03021	Hit	B33R09C17
Q86TT8	MARK3	PV4819	380	3.29824	MAP/microtubule affinity-regulating kinase 3 (MARK3)	2939.47	25416	9398	15944	0.7231	0.0097163	0.06972	Hit	B23R16C03
P33981	TTK	PV3792	381	3.28443	TTK protein kinase (TTK)	18777.4	31209	16398	15883.8	0.9102	0.0097914	0.01833	Hit	B45R15C19

O00238	BMPR1B	NM_001203.1	383	3.26004	bone morphogenetic protein receptor, type IB (BMPR1B)	60197.7	23121	7828	15777.5	0.63972	0.0099262	0.06297	Hit	B43R10C10
P53779	MAPK10	NM_002753.2	386	3.2106	mitogen-activated protein kinase 10 (MAPK10), transcript variant 1	17014	24477	13857	15562.1	0.74805	0.0102081	0.03253	Hit	B02R17C03
P54760	EPHB4	NM_004444.2	388	3.17517	ephrin receptor B4 (EPHB4)	14311.5	22163	6848	15407.6	0.81123	0.0104176	0.03021	Hit	B33R09C18
Q96H79	ZC3HAV1L	NM_080660.2	390	3.16922	zinc finger CCCH-type, antiviral 1-like (ZC3HAV1L)	38752.9	24860	22305	15381.7	0.58696	0.0104534	0.08293	Hit	B02R06C01
O75582	RPS6KA5	NM_004755.2	391	3.15967	ribosomal protein S6 kinase, 90kDa, polypeptide 5 (RPS6KA5), transcript variant 1	23466	22365	10659	15340.1	0.55758	0.0105113	0.13382	Hit	B13R09C21
P21802	FGFR2	PV3368	392	3.15311	fibroblast growth factor receptor 2 (bacteria-expressed kinase, keratinocyte growth factor receptor, craniofacial dysostos	0	25458	13376	15311.5	0.82828	0.0105513	0.00505	Hit	B44R15C22
O14976	GAK	BC008668.1	395	3.12834	cyclin G associated kinase (GAK)	117663	21413	8223	15203.6	0.52518	0.0107045	0.12532	Hit	B41R03C03
P21802	FGFR2	PV3368	396	3.12812	fibroblast growth factor receptor 2 (bacteria-expressed kinase, keratinocyte growth factor receptor, craniofacial dysostos	0	27411	17267	15202.6	0.82828	0.0107059	0.00505	Hit	B44R15C21
Q8IY84	MGC42105	BC036422.1	397	3.1259	hypothetical protein MGC42105 (MGC42105)	3579.23	21877	9540	15192.9	0.71483	0.0107198	0.07461	Hit	B10R17C13
Q99759	MAP3K3	PV3876	399	3.06694	Mitogen-activated protein kinase kinase kinase 3	10388.2	27288	18008	14936	0.59159	0.011099	0.10648	Hit	B35R15C16
Q04912	MST1R	PV4314	401	3.05028	Macrophage-stimulating protein receptor	4519.91	21929	7164	14863.4	0.62725	0.0112098	0.07652	Hit	B14R15C18
P53779	MAPK10	NM_002753.2	402	3.04999	mitogen-activated protein kinase 10 (MAPK10), transcript variant 1	15903.6	23365	9419	14862.1	0.74805	0.0112118	0.03253	Hit	B02R17C04
Q9P1W9	PIM2	NM_006875.1	406	2.97445	Serine/threonine-protein kinase Pim-2	36202.2	28291	19047	14533	0.6927	0.0117358	0.01321	Hit	B48R09C21
Q86TT8	MARK3	PV4819	408	2.95447	MAP/microtubule affinity-regulating kinase 3 (MARK3)	2941.89	21331	6855	14445.9	0.7231	0.0118805	0.06972	Hit	B23R16C04
Q16539	MAPK14	PV3304	410	2.94331	mitogen-activated protein kinase 14 (MAPK14), transcript variant 2	11615.6	27215	22495	14397.3	0.72691	0.0119625	0.0506	Hit	B04R15C20
O75582	RPS6KA5	NM_004755.1	412	2.92241	Ribosomal protein S6 kinase alpha-5	6875.49	32111	21709	14306.2	0.42993	0.0121184	0.08778	Hit	B16R10C07
Q9P1W9	PIM2	NM_006875.1	413	2.91272	Serine/threonine-protein kinase Pim-2	36190.5	25057	14685	14263.9	0.6927	0.0121917	0.01321	Hit	B48R09C22
O14757	CHEK1	P3040	414	2.91222	CHK1 checkpoint homolog (S. pombe) (CHEK1)	0	20894	10318	14261.8	0.51311	0.0121955	0.12283	Hit	B33R15C15
Q9P1W9	PIM2	BC018111.1	415	2.91097	pim-2 oncogene (PIM2)	33444.6	21897	8489	14256.3	0.67461	0.0122051	0.08002	Hit	B39R04C13
P29322	EPHA8	PV3844	417	2.89971	Ephrin receptor A8 (EPHA8), transcript variant 2	14826.3	28932	21105	14207.2	0.80402	0.0122913	0.00899	Hit	B44R15C15
P51957	NEK4	PV4315	418	2.87789	NIMA (never in mitosis gene a)-related kinase 4 (NEK4)	4731.13	24341	18517	14112.1	0.63532	0.0124609	0.07943	Hit	B04R15C18
Q9UK32	RPS6KA6	PV4557	419	2.87477	ribosomal protein S6 kinase, 90kDa, polypeptide 6 (RPS6KA6)	6973.07	21284	10682	14098.6	0.89275	0.0124853	0.01541	Hit	B31R15C19
O15547	P2RXL1	NM_005446.2	420	2.87291	purinergic receptor P2X-like 1, orphan receptor (P2RXL1)	15175.6	34527	57457	14090.4	0.5615	0.0125001	0.12134	Hit	B05R09C02
Q96FZ2	C3orf37	BC009993.2	421	2.8696	chromosome 3 open reading frame 37 (C3orf37)	15083.9	29510	26864	14076	0.63725	0.0125262	0.05336	Hit	B01R04C15
P49760	CLK2	PV4201	423	2.8599	CDC-like kinase 2 (CLK2), transcript variant 1	22100.3	25124	13785	14033.8	0.50402	0.0126033	0.14633	Hit	B09R16C02
P29322	EPHA8	PV3844	424	2.85853	Ephrin receptor A8 (EPHA8), transcript variant 2	12862.3	31102	23585	14027.8	0.80402	0.0126143	0.00899	Hit	B44R15C16
P68400	CSNK2A1	BC011668.1	426	2.85095	Casein kinase II subunit alpha	51056.2	23875	16511	13994.7	0.56004	0.0126752	0.0719	Hit	B24R09C19
Q9UK32	RPS6KA6	PV4557	429	2.80503	ribosomal protein S6 kinase, 90kDa, polypeptide 6 (RPS6KA6)	7328.26	21306	9664	13794.6	0.89275	0.0130534	0.01541	Hit	B31R15C20
Q9UQB9	AURKC	NM_003160.1	430	2.78589	Serine/threonine-protein kinase 13	40843.1	20238	8109	13711.2	0.80836	0.013216	0.03222	Hit	B30R09C17
Q96H79	ZC3HAV1L	NM_080660.2	431	2.77819	zinc finger CCCH-type, antiviral 1-like (ZC3HAV1L)	56247.9	24470	20497	13677.7	0.58696	0.0132823	0.08293	Hit	B02R06C02
Q8IY84	MGC42105	BC036422.1	432	2.7765	hypothetical protein MGC42105 (MGC42105)	2572.94	19994	6895	13670.3	0.71483	0.013297	0.07461	Hit	B10R17C14
P68400	CSNK2A1	NM_177559.2	433	2.77531	casein kinase 2, alpha 1 polypeptide (CSNK2A1), transcript variant 1	42853.2	21545	11462	13665.1	0.5463	0.0133072	0.09085	Hit	B29R07C11
Q9UL54	TAOK2	PV3760	434	2.76834	TAO kinase 2 (TAOK2), transcript variant 1	11450	21917	9099	13634.8	0.66099	0.0133678	0.04192	Hit	B43R15C16
Q9HC98	NEK6	PV3353	435	2.76331	NIMA (never in mitosis gene a)-related kinase 6 (NEK6)	124.9	20797	12459	13612.8	0.92524	0.0134119	0.01069	Hit	B13R15C15
Q96JI9	FAM120B	NM_032448.1	436	2.74209	family with sequence similarity 120B (FAM120B)	2429.74	29776	27070	13520.4	0.68047	0.0135999	0.03707	Hit	B01R05C16
Q9Y664	KPTN	BC009249.1	438	2.72866	kaptin (actin binding protein) (KPTN)	8401.25	30987	38710	13461.8	0.49938	0.013721	0.14076	Hit	B39R14C18
Q9HC98	NEK6	PV3353	440	2.71646	NIMA (never in mitosis gene a)-related kinase 6 (NEK6)	163.97	26277	20615	13408.6	0.92524	0.0138323	0.01069	Hit	B13R15C16
Q16539	MAPK14	PV3304	441	2.71506	mitogen-activated protein kinase 14 (MAPK14), transcript variant 2	26327.1	24368	20550	13402.6	0.72691	0.0138452	0.0506	Hit	B04R15C19
Q04912	MST1R	PV4314	444	2.70016	Macrophage-stimulating protein receptor	4543.04	25342	12353	13337.6	0.62725	0.0139833	0.07652	Hit	B14R15C17
Q13557	CAMK2D	NM_001221.2	445	2.69178	calcium/calmodulin-dependent protein kinase (CaM kinase) II delta (CAMK2D), transcript variant 3	18758.8	22076	14346	13301.1	0.58508	0.0140618	0.03408	Hit	B16R19C17
Q9UQB9	AURKC	NM_003160.1	451	2.64571	Serine/threonine-protein kinase 13	40554.3	20621	8256	13100.3	0.80836	0.0145058	0.03222	Hit	B30R09C18
Q96FZ2	C3orf37	BC009993.2	452	2.63471	chromosome 3 open reading frame 37 (C3orf37)	10817.7	29690	27107	13052.4	0.63725	0.0146149	0.05336	Hit	B01R04C16
P33981	TTK	NM_003318.3	455	2.5925	TTK protein kinase (TTK)	9346.52	20379	12100	12868.4	0.42046	0.0150453	0.17707	Hit	B21R21C03
Q9UL54	TAOK2	PV3760	456	2.58818	TAO kinase 2 (TAOK2), transcript variant 1	2888.84	21029	10111	12849.6	0.66099	0.0150903	0.04192	Hit	B43R15C15
Q99759	MAP3K3	PV3876	457	2.58696	Mitogen-activated protein kinase kinase kinase 3	7299.25	23248	14224	12844.3	0.59159	0.0151031	0.10648	Hit	B35R15C15
Q96JI9	FAM120B	NM_032448.1	458	2.58359	family with sequence similarity 120B (FAM120B)	17086.3	26888	23704	12829.6	0.68047	0.0151385	0.03707	Hit	B01R05C15
Q9P1W9	PIM2	BC018111.1	461	2.5606	pim-2 oncogene (PIM2)	51600.3	20129	7583	12729.4	0.67461	0.0153832	0.08002	Hit	B39R04C14
O14976	GAK	BC008668.1	462	2.56037	cyclin G associated kinase (GAK)	122501	18506	5481	12728.4	0.52518	0.0153857	0.12532	Hit	B41R03C04
O75582	RPS6KA5	NM_004755.2	464	2.5511	ribosomal protein S6 kinase, 90kDa, polypeptide 5 (RPS6KA5), transcript variant 1	25794.3	19585	9781	12688	0.55758	0.0154861	0.13382	Hit	B13R09C22
Q13557	CAMK2D	NM_001221.2	465	2.54815	calcium/calmodulin-dependent protein kinase (CaM kinase) II delta (CAMK2D), transcript variant 3	18112.8	20857	10604	12675.2	0.58508	0.0155182	0.03408	Hit	B16R19C18
P68400	CSNK2A1	BC011668.1	466	2.54022	Casein kinase II subunit alpha	48531.5	21767	10898	12640.6	0.56004	0.0156051	0.0719	Hit	B24R09C20
O75582	RPS6KA5	NM_004755.1	467	2.53872	Ribosomal protein S6 kinase alpha-5	6235.36	30333	19913	12634.1	0.42993	0.0156216	0.08778	Hit	B16R10C08
P51957	NEK4	PV4315	468	2.53348	NIMA (never in mitosis gene a)-related kinase 4 (NEK4)	5642.63	22285	15902	12611.2	0.63532	0.0156795	0.07943	Hit	B04R15C17
P27038	ACVR2A	NM_001616.2	472	2.51173	Activin receptor type-2A	28439.8	20122	11545	12516.5	0.48464	0.0159235	0.14113	Hit	B03R10C09
P50613	CDK7	PV3868	473	2.47638	cyclin-dependent kinase 7	0	25179	20523	12362.4	0.57314	0.0163322	0.03029	Hit	B16R15C14
P53779	MAPK10	NM_002753.2	476	2.41482	mitogen-activated protein kinase 10 (MAPK10), transcript variant 1	16705.7	23773	16404	12094.1	0.57339	0.0170826	0.06288	Hit	B01R10C05
P16591	FER	PV3806	477	2.40458	Proto-oncogene tyrosine-protein kinase FER	13735.2	18398	8854	12049.5	0.70299	0.0172124	0.03418	Hit	B29R15C21
O60285	NUAK1	PV4127	478	2.39945	NUAK family, SNF1-like kinase, 1 (NUAK1)	0	19216	11027	12027.1	0.73569	0.017278	0.02194	Hit	B02R15C21
P68400	CSNK2A1	NM_177559.2	480	2.39676	casein kinase 2, alpha 1 polypeptide (CSNK2A1), transcript variant 1	42375.2	19244	7317	12015.4	0.5463	0.0173125	0.09085	Hit	B29R07C12
O14757	CHEK1	P3040	482	2.38916	CHK1 checkpoint homolog (S. pombe) (CHEK1)	0	19399	8630	11982.3	0.51311	0.0174106	0.12283	Hit	B33R15C16
P50613	CDK7	PV3868	487	2.35742	cyclin-dependent kinase 7	0	20778	16268	11844	0.57314	0.0178297	0.03029	Hit	B16R15C13
P42685	FRK	NM_002031.2	489	2.33693	fyn-related kinase (FRK)	11362.2	17906	8946	11754.7	0.81485	0.0181084	0.04082	Hit	B37R19C01
O60285	NUAK1	PV4127	491	2.31513	NUAK family, SNF1-like kinase, 1 (NUAK1)	0	18396	7388	11659.7	0.73569	0.018412	0.02194	Hit	B02R15C22
P16591	FER	PV3806	493	2.27408	Proto-oncogene tyrosine-protein kinase FER	16648.2	18102	9323	11480.8	0.70299	0.019005	0.03418	Hit	B29R15C22
O14647	CHD2	BC007347.2	506	2.22757	chromodomain helicase DNA binding protein 2 (CHD2)	27996.1	17952	10010	11278.1	0.47212	0.0197122	0.14683	Hit	B09R04C04
O14920	IKBKB	PV3836	513	2.18559	inhibitor of kappa light polypeptide gene enhancer in B-cells, kinase beta (IKBKB)	16373.3	17833	9691	11095.2	0.88862	0.0203849	0.01554	Hit	B37R15C15

P42685	FRK	NM_002031.2	512	2.18559	fyn-related kinase (FRK)	9920.42	17407	6618	11095.2	0.81485	0.0203849	0.04082	Hit	B37R19C02
P53779	MAPK10	NM_002753.2	514	2.17853	mitogen-activated protein kinase 10 (MAPK10), transcript variant 1	15390.3	22937	15245	11064.4	0.57339	0.0205013	0.06288	Hit	B01R10C06
Q9UKB3	DNAJC12	BC017018.1	518	2.13698	DnaJ (Hsp40) homolog, subfamily C, member 12 (DNAJC12)	36749.5	16548	6843	10883.4	0.45497	0.0212077	0.15674	Hit	B34R17C07
O14920	IKBKB	PV3836	520	2.13026	inhibitor of kappa light polypeptide gene enhancer in B-cells, kinase beta (IKBKB)	18967.9	19930	12627	10854	0.88862	0.0213255	0.01554	Hit	B37R15C16
Q9Y3B8	REXO2	BC003502.1	525	2.0995	REX2, RNA exonuclease 2 homolog (S. cerevisiae) (REXO2)	50335.7	17821	10649	10720	0.50005	0.0218767	0.09585	Hit	B08R03C09
Q13627	DYRK1A	NM_001396.2	526	2.09698	Dual specificity tyrosine-phosphorylation-regulated kinase 1A	28861.8	15977	5626	10709	0.86699	0.0219228	0.02182	Hit	B37R12C12
Q86XP3	DDX42	BC015505.1	527	2.09108	DEAD (Asp-Glu-Ala-Asp) box polypeptide 42 (DDX42)	95895.8	25221	22501	10683.3	0.40256	0.0220314	0.10614	Hit	B01R05C19
P05771	PRKCB1	P2281	529	2.08269	protein kinase C, beta 1 (PRKCB1), transcript variant 1	0	18031	10588	10646.8	0.5732	0.022187	0.10692	Hit	B03R15C13
O75676	RPS6KA4	NM_003942.1	530	2.06874	Ribosomal protein S6 kinase alpha-4	8961.75	17082	8666	10586	0.83931	0.0224496	0.02497	Hit	B12R10C05
P54753	EPHB3	NM_004443.2	531	2.06337	Ephrin type-B receptor 3	23261.1	16142	6550	10562.5	0.78925	0.022552	0.03344	Hit	B19R10C01
Q13627	DYRK1A	NM_001396.2	538	2.02231	Dual specificity tyrosine-phosphorylation-regulated kinase 1A	30701.7	16089	7604	10383.6	0.86699	0.0233581	0.02182	Hit	B37R12C11
Q8IV61	RASGRP3	NM_170672.1	541	2.00526	RAS guanyl releasing protein 3 (calcium and DAG-regulated) (RASGRP3)	53771.4	21594	18424	10309.3	0.60874	0.0237055	0.03899	Hit	B01R09C02
Q8N6F1	CLDN19	BC030524.1	542	1.99167	claudin 19 (CLDN19)	49898.5	21698	17086	10250.1	0.66024	0.0239881	0.02251	Hit	B01R05C08
P27038	ACVR2A	NM_001616.2	544	1.99051	Activin receptor type-2A	50663.5	17292	7514	10245	0.48464	0.0240124	0.14113	Hit	B03R10C10
O75676	RPS6KA4	NM_003942.1	545	1.98445	Ribosomal protein S6 kinase alpha-4	8181.11	16323	6547	10218.6	0.83931	0.0241402	0.02497	Hit	B12R10C06
P06213	INSR	PV3781	546	1.98165	Insulin receptor	11444.4	16629	7230	10206.4	0.79679	0.0241996	0.03699	Hit	B42R15C17
Q8IV53	DENND1C	BC033437.1	548	1.97398	DENN/MADD domain containing 1C (DENND1C)	15798.2	23362	19163	10173	0.68555	0.0243636	0.01401	Hit	B01R05C06
P54753	EPHB3	NM_004443.2	555	1.95139	Ephrin type-B receptor 3	22004.3	15842	5418	10074.5	0.78925	0.0248559	0.03344	Hit	B19R10C02
P33981	TTK	NM_003318.3	558	1.93534	TTK protein kinase (TTK)	4017.36	16934	6893	10004.6	0.42046	0.0252148	0.17707	Hit	B21R21C04
Q9H2M3	BHMT2	NM_017614.3	559	1.9334	betaine-homocysteine methyltransferase 2 (BHMT2)	18717.8	24359	23504	9996.14	0.63024	0.0252588	0.02964	Hit	B01R03C15
Q8IV53	DENND1C	BC033437.1	561	1.92818	DENN/MADD domain containing 1C (DENND1C)	15616.7	22296	19456	9973.39	0.68555	0.0253775	0.01401	Hit	B01R05C05
Q9UQB8	BAIAP2	NM_017451.1	566	1.92004	BAI1-associated protein 2 (BAIAP2), transcript variant 2	108158	23475	20917	9937.95	0.58437	0.0255641	0.04333	Hit	B01R07C19
Q8N6F1	CLDN19	BC030524.1	567	1.91796	claudin 19 (CLDN19)	50749.4	22138	18912	9928.87	0.66024	0.0256123	0.02251	Hit	B01R05C07
P04150	GR-LBD	PV4690	568	1.91183	Glucocorticoid receptor	1779.6	18235	14162	9902.17	0.59173	0.0257547	0.11613	Hit	B21R16C03
P11309	PIM1	NM_002648.1	576	1.88037	pim-1 oncogene (PIM1)	18029.9	23755	23082	9765.03	0.63166	0.026505	0.03975	Hit	B44R14C16
Q8IV61	RASGRP3	NM_170672.1	579	1.87832	RAS guanyl releasing protein 3 (calcium and DAG-regulated) (RASGRP3)	50935.6	19988	17915	9756.12	0.60874	0.0265549	0.03899	Hit	B01R09C01
Q86Z02	HIPK1	PV4561	581	1.86512	homeodomain interacting protein kinase 1	7885.22	18509	11286	9698.61	0.77726	0.0268803	0.03131	Hit	B35R15C14
P06213	INSR	PV3781	582	1.86227	Insulin receptor	6361.22	15941	6144	9686.18	0.79679	0.0269514	0.03699	Hit	B42R15C18
P34947	GRK5	PV3824	584	1.84729	G protein-coupled receptor kinase 5 (GRK5)	14960.2	18856	14623	9620.91	0.69723	0.0273295	0.08096	Hit	B21R16C02
Q9H2M3	BHMT2	NM_017614.3	586	1.83922	betaine-homocysteine methyltransferase 2 (BHMT2)	28179.2	24864	23058	9585.75	0.63024	0.0275365	0.02964	Hit	B01R03C16
Q16621	NFE2	NM_006163.1	599	1.7877	nuclear factor (erythroid-derived 2), 45kDa (NFE2)	35792.3	20195	15717	9361.23	0.54135	0.0289153	0.0511	Hit	B01R04C02
Q9Y3B8	REXO2	BC003502.1	600	1.7872	REX2, RNA exonuclease 2 homolog (S. cerevisiae) (REXO2)	29715.1	16272	8515	9359.05	0.50005	0.0289292	0.09585	Hit	B08R03C10
Q9UQB8	BAIAP2	NM_017451.1	601	1.78447	BAI1-associated protein 2 (BAIAP2), transcript variant 2	110742	22469	18233	9347.13	0.58437	0.0290054	0.04333	Hit	B01R07C20
A8KAB6	C18orf25	NM_001008239.1	602	1.78033	chromosome 18 open reading frame 25 (C18orf25), transcript variant 2	13856.4	15134	6471	9329.1	0.53364	0.0291211	0.12119	Hit	B31R09C13
Q9HC98	NEK6	BC004174.1	603	1.77908	Serine/threonine-protein kinase Nek6	28052.3	16192	9142	9323.66	0.65986	0.0291561	0.03601	Hit	B08R17C01
P49841	GSK3B	PV3365	604	1.77616	glycogen synthase kinase 3 beta (GSK3B)	0	15937	7362	9310.91	0.80841	0.0292385	0.03708	Hit	B34R15C21
Q86Z02	HIPK1	PV4561	610	1.7687	homeodomain interacting protein kinase 1	6163.49	16767	9463	9278.42	0.77726	0.0294501	0.03131	Hit	B35R15C13
Q86Y07	VRK2	BC036434.1	611	1.76734	Serine/threonine-protein kinase VRK2	34846.9	14836	8081	9272.47	0.47	0.0294891	0.08718	Hit	B29R18C13
P43490	PBEF1	NM_005746.1	612	1.76189	pre-B-cell colony enhancing factor 1 (PBEF1)	49180.3	21931	18195	9248.74	0.68776	0.0296454	0.00485	Hit	B01R07C16
P11309	PIM1	NM_002648.1	615	1.75784	pim-1 oncogene (PIM1)	18575.3	22467	20784	9231.07	0.63166	0.0297626	0.03975	Hit	B44R14C15
Q86XP3	DDX42	BC015505.1	618	1.7488	DEAD (Asp-Glu-Ala-Asp) box polypeptide 42 (DDX42)	9059.33	21898	18764	9191.69	0.40256	0.0300263	0.10614	Hit	B01R05C20
P43490	PBEF1	NM_005746.1	619	1.74738	pre-B-cell colony enhancing factor 1 (PBEF1)	53609.8	21162	18164	9185.51	0.68776	0.030068	0.00485	Hit	B01R07C15
O14647	CHD2	BC007347.2	620	1.74073	chromodomain helicase DNA binding protein 2 (CHD2)	28039.5	15450	8978	9156.53	0.47212	0.0302647	0.14683	Hit	B09R04C03
P05771	PRKCB1	P2281	621	1.73923	protein kinase C, beta 1 (PRKCB1), transcript variant 1	0	16946	9820	9150	0.5732	0.0303094	0.10692	Hit	B03R15C14
Q96ES0	KIAA1468	NM_020854.2	622	1.73396	KIAA1468 (KIAA1468)	23294.3	18502	15812	9127.03	0.61378	0.030467	0.02638	Hit	B01R06C01
P17948	FLT1	PV3666	627	1.71597	fms-related tyrosine kinase 1 (vascular endothelial growth factor/vascular permeability factor receptor) (FLT1)	4014.44	15428	7804	9048.62	0.64033	0.0310145	0.07657	Hit	B47R15C19
Q9BUV0	C1orf63	NM_020317.2	628	1.70866	chromosome 1 open reading frame 63 (C1orf63)	65842.8	15868	9210	9016.77	0.55177	0.031241	0.11382	Hit	B09R04C05
O43639	NCK2	BC000103.1	632	1.6916	NCK adaptor protein 2 (NCK2)	33719.8	15228	7443	8942.41	0.64391	0.0317799	0.0842	Hit	B31R15C09
Q9HC98	NEK6	BC004174.1	634	1.67283	Serine/threonine-protein kinase Nek6	27831.8	15955	8373	8860.61	0.65986	0.0323889	0.03601	Hit	B08R17C02
P49841	GSK3B	PV3365	636	1.66699	glycogen synthase kinase 3 beta (GSK3B)	0	15236	7869	8835.17	0.80841	0.0325819	0.03708	Hit	B34R15C22
Q96ES0	KIAA1468	NM_020854.2	640	1.65726	KIAA1468 (KIAA1468)	21278.3	19037	15170	8792.74	0.61378	0.0329076	0.02638	Hit	B01R06C02
Q9UKB3	DNAJC12	BC017018.1	642	1.63863	DnaJ (Hsp40) homolog, subfamily C, member 12 (DNAJC12)	37617.6	14170	5041	8711.56	0.45497	0.0335445	0.15674	Hit	B34R17C08
Q16621	NFE2	NM_006163.1	643	1.63788	nuclear factor (erythroid-derived 2), 45kDa (NFE2)	36717.4	18290	15725	8708.33	0.54135	0.0335703	0.0511	Hit	B01R04C01
P34947	GRK5	PV3824	649	1.60821	G protein-coupled receptor kinase 5 (GRK5)	18275.9	16542	13463	8579.01	0.69723	0.034625	0.08096	Hit	B21R16C01
Q9BRL6	SFRS2B	BC057783.1	656	1.58117	splicing factor, arginine/serine-rich 2B (SFRS2B)	37181.1	14341	7492	8461.15	0.41958	0.0356301	0.16129	Hit	B34R09C11
P04150	GR-LBD	PV4690	660	1.56698	Glucocorticoid receptor	1844.71	16229	10757	8399.32	0.59173	0.036175	0.11613	Hit	B21R16C04
Q92772	CDKL2	NM_003948.2	666	1.54958	Cyclin-dependent kinase-like 2	6861.83	14736	7408	8323.52	0.66469	0.0368603	0.02574	Hit	B08R10C04
P61923	COPZ1	NM_016057.1	668	1.52781	coatomer protein complex, subunit zeta 1 (COPZ1)	19484.8	21458	19022	8228.61	0.61955	0.0377461	0.01426	Hit	B01R03C20
Q96B23	C18orf25	NM_145055.1	670	1.52662	Uncharacterized protein C18orf25	13309.9	14570	9138	8223.43	0.65206	0.0377954	0.03236	Hit	B14R06C19
P53350	PLK1	PV3501	672	1.52261	polo-like kinase 1 (Drosophila) (PLK1)	0	13611	6642	8205.98	0.72626	0.037962	0.05315	Hit	B26R15C16
Q86Y07	VRK2	BC036434.1	674	1.52023	Serine/threonine-protein kinase VRK2	36882.1	13909	5787	8195.62	0.47	0.0380615	0.08718	Hit	B29R18C14
P49840	GSK3A	PV3270	675	1.51978	glycogen synthase kinase 3 alpha (GSK3A)	0	13626	5121	8193.64	0.76838	0.0380806	0.04695	Hit	B37R15C20
P17948	FLT1	PV3666	679	1.50266	fms-related tyrosine kinase 1 (vascular endothelial growth factor/vascular permeability factor receptor) (FLT1)	4683.84	14260	6495	8119.05	0.64033	0.0388089	0.07657	Hit	B47R15C20
Q0VDG4	SCRN3	BC031821.1	681	1.49837	Secernin-3	9646.76	23709	21606	8100.34	0.57647	0.038995	0.02574	Hit	B01R03C17
P61923	COPZ1	NM_016057.1	684	1.4901	coatomer protein complex, subunit zeta 1 (COPZ1)	57615.1	22104	20529	8064.3	0.61955	0.039357	0.01426	Hit	B01R03C19
Q92772	CDKL2	NM_003948.2	688	1.48129	Cyclin-dependent kinase-like 2	7661.98	15208	9734	8025.9	0.66469	0.0397484	0.02574	Hit	B08R10C03

Q12866	MERTK	NM_006343.1	690	1.47855	Proto-oncogene tyrosine-protein kinase MER	62196.8	13349	5902	8013.95	0.48837	0.0398713	0.09159	Hit	B25R09C20
O96028	WHSC1	NM_133336.1	691	1.47452	Wolf-Hirschhorn syndrome candidate 1 (WHSC1), transcript variant 9	6268.54	13718	6275	7996.42	0.54258	0.0400528	0.11275	Hit	B31R08C09
Q06187	BTK	NM_000061.1	696	1.46591	Bruton agammaglobulinemia tyrosine kinase (BTK)	85751.1	19021	13374	7958.88	0.63665	0.0404454	0.00553	Hit	B01R10C02
O43639	NCK2	BC000103.1	699	1.46098	NCK adaptor protein 2 (NCK2)	29342.3	13921	5897	7937.4	0.64391	0.0406728	0.0842	Hit	B31R15C10
P42680	TEC	PV3269	700	1.45963	tec protein tyrosine kinase (TEC)	0	17088	13838	7931.52	0.43658	0.0407353	0.10692	Hit	B25R16C02
Q06187	BTK	NM_000061.1	703	1.45168	Bruton agammaglobulinemia tyrosine kinase (BTK)	103470	17914	13038	7896.88	0.63665	0.0411068	0.00553	Hit	B01R10C01
A8KAB6	C18orf25	NM_001008239.1	707	1.4424	chromosome 18 open reading frame 25 (C18orf25), transcript variant 2	10837.2	13390	5446	7856.42	0.53364	0.0415472	0.12119	Hit	B31R09C14
Q96B23	C18orf25	NM_145055.1	708	1.4422	Uncharacterized protein C18orf25	17462.9	13855	7281	7855.56	0.65206	0.0415566	0.03236	Hit	B14R06C20
P61764	STXBP1	NM_003165.1	709	1.43818	Syntaxin-binding protein 1	76498.8	19458	15869	7838.02	0.56578	0.0417498	0.04151	Hit	B44R17C19
Q0VDG4	SCRN3	BC031821.1	711	1.43193	Secernin-3	44000.8	23065	21539	7810.79	0.57647	0.0420524	0.02574	Hit	B01R03C18
Q9H7X2	C1orf115	NM_024709.2	713	1.42349	chromosome 1 open reading frame 115 (C1orf115)	8000.96	29417	45084	7774.02	0.59171	0.0424662	0.01304	Hit	B43R09C02
P19784	CSNK2A2	NM_001896.2	714	1.42336	casein kinase 2, alpha prime polypeptide (CSNK2A2)	31448.1	13090	7755	7773.47	0.66714	0.0424724	0.07096	Hit	B10R06C05
Q9BUV0	C1orf63	NM_020317.2	721	1.40042	chromosome 1 open reading frame 63 (C1orf63)	63829	13374	6131	7673.49	0.55177	0.0436294	0.11382	Hit	B09R04C06
P49840	GSK3A	PV3270	723	1.39894	glycogen synthase kinase 3 alpha (GSK3A)	0	16386	7514	7667.03	0.76838	0.0437057	0.04695	Hit	B37R15C19
Q9H7X2	C1orf115	NM_024709.2	725	1.3909	chromosome 1 open reading frame 115 (C1orf115)	6071.86	23939	32650	7632.01	0.59171	0.0441232	0.01304	Hit	B43R09C01
P53350	PLK1	PV3501	727	1.38621	polo-like kinase 1 (Drosophila) (PLK1)	0	12885	6643	7611.57	0.72626	0.0443697	0.05315	Hit	B26R15C15
P36888	FLT3	PV3182	732	1.377	fms-related tyrosine kinase 3 (FLT3); see catalog number for detailed information on wild-type or point mutant status	0	14542	9136	7571.43	0.54616	0.0448597	0.10179	Hit	B03R15C17
Q9BRS1	C6orf134	BC006105.1	738	1.37189	chromosome 6 open reading frame 134 (C6orf134)	0	14212	10806	7549.17	0.43372	0.0451351	0.13286	Hit	B05R06C01
Q15375	EPHA7	PV3689	744	1.34899	Ephrin type-A receptor 7	4444.18	14008	10079	7449.34	0.75353	0.0464012	0.04111	Hit	B12R15C15
Q12851	MAP4K2	PV4211	748	1.3426	mitogen-activated protein kinase kinase kinase kinase 2 (MAP4K2)	0	13065	8866	7421.52	0.42743	0.0467635	0.11527	Hit	B33R16C01
P61764	STXBP1	NM_003165.1	754	1.3356	Syntaxin-binding protein 1	103093	18768	14530	7391.01	0.56578	0.0471658	0.04151	Hit	B44R17C20
O95452	GJB6	BC038934.1	757	1.32771	gap junction protein, beta 6 (GJB6)	35667.4	16069	14754	7356.6	0.40308	0.0476259	0.08242	Hit	B02R04C01
Q9BST9	RTKN	NM_033046.1	759	1.32499	rhotekin (RTKN), transcript variant 2	34197.9	22703	23031	7344.77	0.56905	0.0477856	0.08415	Hit	B35R14C21
Q8TBR0	PDE4DIP	BC026270.1	769	1.31078	phosphodiesterase 4D interacting protein (myomegalin) (PDE4DIP)	10513.3	13000	7096	7282.84	0.79907	0.0486348	0.02484	Hit	B26R17C05
Q9ULE6	KIAA1274	NM_014431.1	779	1.29415	Paladin	38152.9	14569	9971	7210.37	0.92477	0.0496577	0.00018	Hit	B21R19C03
Q9ULE6	KIAA1274	NM_014431.1	781	1.29374	Paladin	35835.5	13227	7048	7208.57	0.92477	0.0496835	0.00018	Hit	B21R19C04
Q8N5Y2	MSL3L1	NM_078630.1	782	1.29216	male-specific lethal 3-like 1 (Drosophila) (MSL3L1), transcript variant 2	45095.3	15153	13426	7201.72	0.73873	0.049782	0.01474	Hit	B33R07C02
P54753	EPHB3	PV3658	797	1.2582	Ephrin type-B receptor 3	20084.4	11797	4957	7053.7	0.78804	0.0519823	0.0064	Hit	B41R16C02
Q8N5Y2	MSL3L1	NM_078630.1	798	1.25808	male-specific lethal 3-like 1 (Drosophila) (MSL3L1), transcript variant 2	59963.8	12731	8351	7053.17	0.73873	0.0519904	0.01474	Hit	B33R07C01
Q12866	MERTK	NM_006343.1	800	1.25485	Proto-oncogene tyrosine-protein kinase MER	66496.3	12245	6318	7039.1	0.48837	0.0522072	0.09159	Hit	B25R09C19
Q8TBR0	PDE4DIP	BC026270.1	801	1.25309	phosphodiesterase 4D interacting protein (myomegalin) (PDE4DIP)	9558.94	12580	5619	7031.42	0.79907	0.052326	0.02484	Hit	B26R17C06
P19784	CSNK2A2	NM_001896.2	803	1.2529	casein kinase 2, alpha prime polypeptide (CSNK2A2)	54445.8	11972	4957	7030.61	0.66714	0.0523387	0.07096	Hit	B10R06C06
Q15375	EPHA7	PV3689	805	1.25242	Ephrin type-A receptor 7	5280.93	14063	9560	7028.51	0.75353	0.0523713	0.04111	Hit	B12R15C16
Q9H4G0	EPB41L1	NM_177996.1	807	1.24857	erythrocyte membrane protein band 4.1-like 1 (EPB41L1), transcript variant 2	8134.27	18496	16096	7011.72	0.45323	0.052633	0.04637	Hit	B01R05C11
P54753	EPHB3	PV3658	810	1.24363	Ephrin type-B receptor 3	23641.2	11599	6551	6990.19	0.78804	0.0529713	0.0064	Hit	B41R16C01
Q8IV03	C9orf150	BC036923.1	814	1.23604	chromosome 9 open reading frame 150 (C9orf150)	22818.1	15920	12655	6957.12	0.50711	0.0534976	0.04534	Hit	B02R04C05
P42680	TEC	PV3269	823	1.20376	tec protein tyrosine kinase (TEC)	0	13193	9005	6816.48	0.43658	0.0558255	0.10692	Hit	B25R16C01
O96028	WHSC1	NM_133336.1	824	1.20353	Wolf-Hirschhorn syndrome candidate 1 (WHSC1), transcript variant 9	6857.92	12419	5365	6815.48	0.54258	0.0558426	0.11275	Hit	B31R08C10
Q66K64	LOC90379	NM_138353.1	829	1.19865	DDB1- and CUL4-associated factor 15	10299	18837	12794	6794.19	0.57798	0.0562083	0.09411	Hit	B12R16C21
Q9BRL6	SFRS2B	BC057783.1	838	1.18363	splicing factor, arginine/serine-rich 2B (SFRS2B)	31765.1	12422	5793	6728.75	0.41958	0.0573554	0.16129	Hit	B34R09C12
Q9H6T3	FLJ21908	BC056415.1	848	1.17076	RNA polymerase II-associated protein 3	38692.4	13067	7583	6672.63	0.42213	0.0583673	0.12477	Hit	B35R19C11
O75251	NDUFS7	BC001715.2	849	1.16957	NADH dehydrogenase (ubiquinone) Fe-S protein 7, 20kDa (NADH-coenzyme Q reductase) (NDUFS7)	18674.9	18836	16536	6667.46	0.43517	0.0584619	0.04537	Hit	B01R03C11
Q9H4G0	EPB41L1	NM_177996.1	867	1.14641	erythrocyte membrane protein band 4.1-like 1 (EPB41L1), transcript variant 2	7464.87	17947	15020	6566.54	0.45323	0.0603548	0.04637	Hit	B01R05C12
P36888	FLT3	PV3182	869	1.14369	fms-related tyrosine kinase 3 (FLT3); see catalog number for detailed information on wild-type or point mutant status	0	12813	6558	6554.7	0.54616	0.0605829	0.10179	Hit	B03R15C18
O95452	GJB6	BC038934.1	871	1.14178	gap junction protein, beta 6 (GJB6)	39658.5	16341	14751	6546.35	0.40308	0.0607444	0.08242	Hit	B02R04C02
Q9BUB5	MKNK1	BC002755.1	872	1.1374	MAP kinase-interacting serine/threonine-protein kinase 1	41791.7	14208	7667	6527.27	0.75149	0.0611162	0.01699	Hit	B30R09C19
Q8IV03	C9orf150	BC036923.1	874	1.13686	chromosome 9 open reading frame 150 (C9orf150)	19592.1	14807	12085	6524.9	0.50711	0.0611627	0.04534	Hit	B02R04C06
Q9BST9	RTKN	NM_033046.1	875	1.13568	rhotekin (RTKN), transcript variant 2	36744.7	19893	14328	6519.76	0.56905	0.0612635	0.08415	Hit	B35R14C22
P31749	AKT1	PV3685	894	1.1119	RAC-alpha serine/threonine-protein kinase	1586.65	16577	9233	6416.15	0.63293	0.0633509	0.0288	Hit	B25R16C05
Q9BUB5	MKNK1	BC002755.1	902	1.10184	MAP kinase-interacting serine/threonine-protein kinase 1	42191.7	11993	5410	6372.29	0.75149	0.0642667	0.01699	Hit	B30R09C20
P04626	ERBB2	NM_004448.1	904	1.09903	Receptor tyrosine-protein kinase erbB-2	10845	15845	10702	6360.04	0.54992	0.064526	0.00498	Hit	B01R10C10
P04626	ERBB2	NM_004448.1	912	1.08879	Receptor tyrosine-protein kinase erbB-2	12016	16216	11628	6315.45	0.54992	0.0654835	0.00498	Hit	B01R10C09
Q12851	MAP4K2	PV4211	916	1.08591	mitogen-activated protein kinase kinase kinase kinase 2 (MAP4K2)	0	12086	6989	6302.89	0.42743	0.065757	0.11527	Hit	B33R16C02
P11362	FGFR1	PV3146	917	1.08182	fibroblast growth factor receptor 1 (fms-related tyrosine kinase 2, Pfeiffer syndrome) (FGFR1), transcript variant 7	0	11835	6421	6285.07	0.78275	0.0661481	0.02592	Hit	B31R15C13
Q9H0R8	GABARAPL1	NM_031412.1	919	1.07889	GABA(A) receptor-associated protein like 1 (GABARAPL1)	0	11892	5828	6272.31	0.58202	0.0664302	0.07676	Hit	B47R03C07
O75251	NDUFS7	BC001715.2	923	1.07445	NADH dehydrogenase (ubiquinone) Fe-S protein 7, 20kDa (NADH-coenzyme Q reductase) (NDUFS7)	1715.46	18230	15237	6252.93	0.43517	0.066862	0.04537	Hit	B01R03C12
Q9BRS1	C6orf134	BC006105.1	924	1.07436	chromosome 6 open reading frame 134 (C6orf134)	47757.6	13408	9392	6252.54	0.43372	0.066871	0.13286	Hit	B05R06C02
Q6P1R4	DUS1L	NM_022156.3	929	1.06097	dihydrouridine synthase 1-like (S. cerevisiae) (DUS1L)	62740.1	11816	6060	6194.2	0.51896	0.0681973	0.09427	Hit	B05R10C06
P31749	AKT1	PV3685	932	1.05314	RAC-alpha serine/threonine-protein kinase	1613.97	12530	7252	6160.06	0.63293	0.0689919	0.0288	Hit	B25R16C06
Q62915	CASK	NM_003688.1	945	1.03002	Peripheral plasma membrane protein CASK	33021.1	11846	6278	6059.32	0.80342	0.0714193	0.02471	Hit	B34R10C09
P11362	FGFR1	PV3146	946	1.0299	fibroblast growth factor receptor 1 (fms-related tyrosine kinase 2, Pfeiffer syndrome) (FGFR1), transcript variant 7	0	11420	5672	6058.8	0.78275	0.0714321	0.02592	Hit	B31R15C14
Q9BW71	HIRIP3	NM_003609.2	957	1.01454	HIRA interacting protein 3 (HIRIP3)	40765	14711	10273	5991.84	0.55175	0.0731176	0.01677	Hit	B44R19C22
Q66K64	LOC90379	NM_138353.1	965	1.00409	DDB1- and CUL4-associated factor 15	36793.5	16106	10676	5946.32	0.57798	0.0742976	0.09411	Hit	B12R16C22
P68106	FKBP1B	NM_054033.1	970	0.9993	FK506 binding protein 1B, 12.6 kDa (FKBP1B), transcript variant 2	127740	11892	6790	5925.44	0.58597	0.0748486	0.00705	Hit	B29R15C09
P68106	FKBP1B	NM_054033.1	975	0.98581	FK506 binding protein 1B, 12.6 kDa (FKBP1B), transcript variant 2	127693	11517	5710	5866.66	0.58597	0.0764325	0.00705	Hit	B29R15C10
Q9BW71	HIRIP3	NM_003609.2	978	0.98231	HIRA interacting protein 3 (HIRIP3)	31563.2	15540	12426	5851.4	0.55175	0.0768522	0.01677	Hit	B44R19C21

Q62915	CASK	NM_003688.1	979	0.98226	Peripheral plasma membrane protein CASK	35784.5	11614	6192	5851.18	0.80342	0.0768582	0.02471	Hit	B34R10C10
P84022	SMAD3	NM_005902.1	983	0.97865	SMAD family member 3 (SMAD3)	51987	11057	6565	5835.47	0.64334	0.0772938	0.08463	Hit	B45R20C03
Q96RG2	PASK	PV3972	999	0.95865	PAS domain containing serine/threonine kinase (PASK)	8169.4	11772	6961	5748.29	0.46559	0.0797791	0.00994	Hit	B43R15C18
Q96RG2	PASK	PV3972	1008	0.94024	PAS domain containing serine/threonine kinase (PASK)	7316.55	12917	7623	5668.09	0.46559	0.0821731	0.00994	Hit	B43R15C17
Q8N7W4	C20orf198	NM_139016.2	1014	0.93611	chromosome 20 open reading frame 198 (C20orf198)	127695	16377	15216	5650.09	0.4945	0.0827251	0.0049	Hit	B01R09C09
P06493	CDC2	NM_001786.2	1016	0.93433	cell division cycle 2, G1 to S and G2 to M (CDC2), transcript variant 1	49574.6	12004	8733	5642.33	0.63753	0.0829647	0.04567	Hit	B35R03C01
Q9H0R8	GABARAPL1	NM_031412.1	1019	0.93069	GABA(A) receptor-associated protein like 1 (GABARAPL1)	18524.2	11081	5241	5626.46	0.58202	0.0834585	0.07676	Hit	B47R03C08
Q8N7W4	C20orf198	NM_139016.2	1020	0.92717	chromosome 20 open reading frame 198 (C20orf198)	127605	16160	13292	5611.09	0.4945	0.0839405	0.0049	Hit	B01R09C10
Q9H6T3	FLJ21908	BC056415.1	1023	0.92248	RNA polymerase II-associated protein 3	36559.3	12026	6490	5590.69	0.42213	0.0845871	0.12477	Hit	B35R19C12
Q9Y2Y9	KLF13	BC012782.2	1041	0.90469	Kruppel-like factor 13 (KLF13)	1757.9	16201	12647	5513.14	0.47783	0.0871138	0.06679	Hit	B04R16C20
A8K830	LOC120376	XM_071712.11	1050	0.89499	Uncharacterized protein LOC120376	46472.2	11215	6266	5470.89	0.84175	0.0885384	0.00809	Hit	B34R07C17
Q9UKI2	CDC42EP3	NM_006449.2	1051	0.89484	CDC42 effector protein (Rho GTPase binding) 3 (CDC42EP3)	28922.1	12469	9762	5470.23	0.64921	0.088561	0.04438	Hit	B39R12C19
Q6P1R4	DUS1L	NM_022156.3	1061	0.88332	dihydrouridine synthase 1-like (S. cerevisiae) (DUS1L)	63661.2	11756	8520	5420.02	0.51896	0.0903005	0.09427	Hit	B05R10C05
A8K830	LOC120376	XM_071712.11	1062	0.88071	Uncharacterized protein LOC120376	64798.7	10786	5063	5408.63	0.84175	0.0907025	0.00809	Hit	B34R07C18
NA	RP4-662A9.2	NM_153224.2	1066	0.87485	hypothetical protein MGC34034 (MGC34034)	5495.54	12028	9112	5383.11	0.56144	0.0916125	0.06596	Hit	B35R12C20
O95171	SCEL	BC047536.1	1072	0.86877	sciellin (SCEL)	106189	11214	6465	5356.6	0.40848	0.0925722	0.13565	Hit	B10R02C11
Q92541	RTF1	NM_015138.2	1073	0.86857	Rtf1, Paf1/RNA polymerase II complex component, homolog (S. cerevisiae) (RTF1)	71922.3	19553	18768	5355.72	0.54686	0.0926042	0.00401	Hit	B44R16C19
O43353	RIPK2	PV4213	1079	0.86689	receptor-interacting serine-threonine kinase 2 (RIPK2)	0	11099	6908	5348.41	0.62653	0.0928718	0.05637	Hit	B03R15C21
Q92541	RTF1	NM_015138.2	1082	0.86162	Rtf1, Paf1/RNA polymerase II complex component, homolog (S. cerevisiae) (RTF1)	77720.5	18684	17656	5325.47	0.54686	0.0937186	0.00401	Hit	B44R16C20
P05771	PRKCB1	P2251	1084	0.86058	protein kinase C, beta 1 (PRKCB1), transcript variant 2	0	14128	7830	5320.94	0.70774	0.0938873	0.06181	Hit	B21R15C19
P06493	CDC2	NM_001786.2	1093	0.85333	cell division cycle 2, G1 to S and G2 to M (CDC2), transcript variant 1	48142.1	16593	13690	5289.33	0.63753	0.0950767	0.04567	Hit	B35R03C02
Q9H2K8	TAOK3	NM_016281.2	1097	0.85144	TAO kinase 3 (TAOK3)	5443.01	10079	4866	5281.09	0.64889	0.0953901	0.05681	Hit	B42R09C02
Q13895	BYSL	BC050645.1	1104	0.84445	bystin-like (BYSL)	59469.2	10692	6349	5250.62	0.67043	0.0965642	0.06146	Hit	B34R04C11
P31751	AKT2	NM_001626.2	1105	0.84219	v-akt murine thymoma viral oncogene homolog 2 (AKT2)	62852.7	10738	5757	5240.79	0.40542	0.0969474	0.12085	Hit	B47R12C09
P84022	SMAD3	NM_005902.1	1116	0.82743	SMAD family member 3 (SMAD3)	38753.3	10454	5627	5176.45	0.64334	0.0995134	0.08463	Hit	B45R20C04
Q9UKI2	CDC42EP3	NM_006449.2	1130	0.81845	CDC42 effector protein (Rho GTPase binding) 3 (CDC42EP3)	41941.8	11880	8299	5137.34	0.64921	0.1011231	0.04438	Hit	B39R12C20
Q9Y698	CACNG2	NM_006078.2	1138	0.80941	calcium channel, voltage-dependent, gamma subunit 2 (CACNG2)	20904.3	17046	13127	5097.92	0.41974	0.1027856	0.01083	Hit	B01R06C06
P13693	TPT1	NM_003295.1	1139	0.80863	tumor protein, translationally-controlled 1 (TPT1)	55674	14554	13451	5094.52	0.64656	0.1029309	0.08004	Hit	B21R17C01
Q9NQC1	PHF15	BC004292.1	1152	0.80111	PHD finger protein 15 (PHF15)	68475.8	9762	5593	5061.75	0.5444	0.1043476	0.1128	Hit	B21R12C16
Q96PF2	TSSK2	NM_053006.1	1157	0.79884	testis-specific serine kinase 2 (TSSK2)	10359.1	10358	5676	5051.85	0.59204	0.1047811	0.05634	Hit	B39R12C10
Q04771	ACVR1	PV4877	1164	0.79183	Activin receptor type-1	42275.6	10317	6751	5021.3	0.61336	0.1061364	0.07768	Hit	B37R16C05
Q9Y698	CACNG2	NM_006078.2	1166	0.79162	calcium channel, voltage-dependent, gamma subunit 2 (CACNG2)	23805.6	16519	13436	5020.4	0.41974	0.1061767	0.01083	Hit	B01R06C05
Q8IW41	MAPKAPK5	NM_003668.2	1170	0.79087	mitogen-activated protein kinase-activated protein kinase 5 (MAPKAPK5), transcript variant 1	43622.2	10996	7845	5017.13	0.68666	0.1063237	0.03287	Hit	B03R09C19
Q9Y2Y9	KLF13	BC012782.2	1171	0.79059	Kruppel-like factor 13 (KLF13)	3932.47	14771	12409	5015.89	0.47783	0.1063794	0.06679	Hit	B04R16C19
Q96CN4	EVI5L	NM_145245.1	1182	0.77973	ecotropic viral integration site 5-like (EVI5L)	42495.8	9771	5197	4968.58	0.55397	0.1085384	0.06791	Hit	B05R15C08
Q32P40	PCLO	BC001304.1	1190	0.77388	piccolo (presynaptic cytomatrix protein) (PCLO)	127746	10864	8495	4943.1	0.73474	0.1097282	0.05043	Hit	B21R10C01
O43353	RIPK2	PV4213	1191	0.7728	receptor-interacting serine-threonine kinase 2 (RIPK2)	0	10298	5491	4938.37	0.62653	0.1099512	0.05637	Hit	B03R15C22
NA	RP4-662A9.2	NM_153224.2	1200	0.76476	hypothetical protein MGC34034 (MGC34034)	5584.76	11352	8901	4903.36	0.56144	0.1116239	0.06596	Hit	B35R12C19
P05771	PRKCB1	P2251	1206	0.75833	protein kinase C, beta 1 (PRKCB1), transcript variant 2	0	10504	5226	4875.33	0.70774	0.1129906	0.06181	Hit	B21R15C20
Q9H2K8	TAOK3	NM_016281.2	1207	0.75784	TAO kinase 3 (TAOK3)	5489.85	9640	5185	4873.19	0.64889	0.1130962	0.05681	Hit	B42R09C01
P45984	MAPK9	PV3620	1212	0.75316	mitogen-activated protein kinase 9 (MAPK9), transcript variant JNK2-a2	0	11270	8941	4852.8	0.47631	0.1141075	0.00789	Hit	B02R16C01
Q13895	BYSL	BC050645.1	1223	0.74409	bystin-like (BYSL)	106829	10304	5545	4813.26	0.67043	0.1161077	0.06146	Hit	B34R04C12
P45984	MAPK9	PV3620	1227	0.74081	mitogen-activated protein kinase 9 (MAPK9), transcript variant JNK2-a2	0	11745	9950	4798.98	0.47631	0.1168434	0.00789	Hit	B02R16C02
Q8IW41	MAPKAPK5	NM_003668.2	1231	0.73858	mitogen-activated protein kinase-activated protein kinase 5 (MAPKAPK5), transcript variant 1	43491.4	10810	7839	4789.24	0.68666	0.1173491	0.03287	Hit	B03R09C20
P61328	FGF12	NM_004113.3	1253	0.71617	fibroblast growth factor 12 (FGF12), transcript variant 2	32390.7	10193	7293	4691.6	0.58014	0.1226043	0.09637	Hit	B13R09C03
P08069	IGF1R	PV3250	1258	0.71333	insulin-like growth factor 1 receptor (IGF1R)	0	9250	4578	4679.2	0.49565	0.1232968	0.06501	Hit	B41R15C20
P18615	RDBP	NM_002904.4	1266	0.71038	RD RNA binding protein (RDBP)	127752	10395	6789	4666.38	0.41588	0.1240193	0.02349	Hit	B29R16C15
Q96PF2	TSSK2	NM_053006.1	1267	0.71001	testis-specific serine kinase 2 (TSSK2)	9946.95	10144	5527	4664.76	0.59204	0.124111	0.05634	Hit	B39R12C09
Q32P40	PCLO	BC001304.1	1279	0.69577	piccolo (presynaptic cytomatrix protein) (PCLO)	127748	11613	9217	4602.69	0.73474	0.1277029	0.05043	Hit	B21R10C02
P13693	TPT1	NM_003295.1	1291	0.68339	tumor protein, translationally-controlled 1 (TPT1)	68620.7	14990	11528	4548.76	0.64656	0.1309519	0.08004	Hit	B21R17C02
O15111	CHUK	PV4310	1296	0.67965	conserved helix-loop-helix ubiquitous kinase (CHUK)	0	10612	6723	4532.45	0.72438	0.1319595	0.00942	Hit	B47R15C17
P18615	RDBP	NM_002904.4	1301	0.6754	RD RNA binding protein (RDBP)	127740	10319	6645	4513.92	0.41588	0.1331175	0.02349	Hit	B29R16C16
Q96CN4	EVI5L	NM_145245.1	1302	0.67524	ecotropic viral integration site 5-like (EVI5L)	37079.8	9664	6048	4513.23	0.55397	0.1331609	0.06791	Hit	B05R15C07
Q04771	ACVR1	PV4877	1306	0.67184	Activin receptor type-1	53175.3	9543	4969	4498.41	0.61336	0.1340988	0.07768	Hit	B37R16C06
O15111	CHUK	PV4310	1313	0.66589	conserved helix-loop-helix ubiquitous kinase (CHUK)	0	10970	7117	4472.48	0.72438	0.1357641	0.00942	Hit	B47R15C18
Q9Y675	SNURF	NM_005678.3	1314	0.66557	SNRPN upstream reading frame (SNURF), transcript variant 1	127711	9521	6133	4471.1	0.50611	0.1358533	0.07423	Hit	B05R09C11
O95171	SCEL	BC047536.1	1329	0.6536	sciellin (SCEL)	118579	9932	5096	4418.92	0.40848	0.1393028	0.13565	Hit	B10R02C12
P31751	AKT2	NM_001626.2	1332	0.65284	v-akt murine thymoma viral oncogene homolog 2 (AKT2)	70651.1	9437	5013	4415.6	0.40542	0.1395266	0.12085	Hit	B47R12C10
O43602	DCX	NM_178151.1	1350	0.64304	doublecortex; lissencephaly, X-linked (doublecortin) (DCX), transcript variant 4	47593.7	13675	9782	4372.91	0.51729	0.1424546	0.07001	Hit	B19R16C13
P62857	RPS28	NM_001031.4	1358	0.64092	40S ribosomal protein S28	32861.1	9632	6276	4363.66	0.76232	0.143101	0.01125	Hit	B10R18C03
Q9UHJ3	SFMBT1	NM_001005158.1	1360	0.63964	Scm-like with four MBT domains protein 1	28579.7	9167	5253	4358.1	0.46351	0.1434917	0.10478	Hit	B26R19C15
Q9NQC1	PHF15	BC004292.1	1371	0.6295	PHD finger protein 15 (PHF15)	67652.2	9133	5554	4313.9	0.5444	0.1466554	0.1128	Hit	B21R12C15
P62857	RPS28	NM_001031.4	1376	0.62511	40S ribosomal protein S28	34642.9	9269	5662	4294.77	0.76232	0.1480571	0.01125	Hit	B10R18C04
Q05397	PTK2	NM_005607.1	1380	0.62281	Focal adhesion kinase 1	62351.7	9136	4641	4284.76	0.53561	0.1487993	0.06249	Hit	B19R10C05
Q9UK59	DBR1	NM_016216.2	1385	0.61957	Lariat debranching enzyme	9286.23	9536	5552	4270.61	0.47854	0.1498568	0.09731	Hit	B42R19C09
P08069	IGF1R	PV3250	1386	0.61895	insulin-like growth factor 1 receptor (IGF1R)	0	10986	5903	4267.9	0.49565	0.1500605	0.06501	Hit	B41R15C19

Q9H7L9	SUDS3	BC093990.1	1393	0.61452	Sin3 histone deacetylase corepressor complex component SDS3	0	9355	5169	4248.61	0.5907	0.1515241	0.04599	Hit	B47R18C14
Q16816	PHKG1	PV3853	1394	0.61375	Phosphorylase b kinase gamma catalytic chain, skeletal muscle isoform	2642.87	9524	6140	4245.24	0.51274	0.1517819	0.06252	Hit	B15R15C17
P61328	FGF12	NM_004113.3	1452	0.57881	fibroblast growth factor 12 (FGF12), transcript variant 2	32289.2	9689	6878	4092.99	0.58014	0.1641538	0.09637	Hit	B13R09C04
P51813	BMX	NM_001721.2	1455	0.57718	BMX non-receptor tyrosine kinase (BMX), transcript variant 2	90948.5	9338	7210	4085.87	0.66174	0.164769	0.03832	Hit	B10R10C01
Q14653	IRF3	NM_001571.2	1457	0.57662	interferon regulatory factor 3 (IRF3)	56158.7	9549	6311	4083.47	0.72297	0.1649777	0.04646	Hit	B21R09C04
Q96CW6	SLC7A6OS	BC013778.1	1458	0.57569	solute carrier family 7, member 6 opposite strand (SLC7A6OS)	17282.4	9811	6271	4079.4	0.48241	0.1653315	0.04176	Hit	B33R04C19
Q9UQB9	AURKC	NM_003160.1	1479	0.56506	Serine/threonine-protein kinase 13	7753.71	9328	5845	4033.05	0.6563	0.1694431	0.02103	Hit	B47R12C07
Q96QK1	VPS35	NM_018206.3	1480	0.56415	vacuolar protein sorting 35 homolog (S. cerevisiae) (VPS35)	21413.1	9630	5419	4029.1	0.55742	0.1698006	0.07097	Hit	B31R09C21
Q9Y675	SNURF	NM_005678.3	1486	0.56324	SNRPN upstream reading frame (SNURF), transcript variant 1	122662	8992	5521	4025.14	0.50611	0.1701604	0.07423	Hit	B05R09C12
P19139	CSNK2A1	PV3248	1489	0.56243	casein kinase 2, alpha 1 polypeptide (CSNK2A1), transcript variant 2	0	13626	8388	4021.61	0.66738	0.170482	0.01531	Hit	B05R15C17
Q5T0L3	C1orf111	BC032957.1	1493	0.55945	chromosome 1 open reading frame 111 (C1orf111)	3918.5	8518	5405	4008.64	0.4068	0.1716714	0.06256	Hit	B33R12C19
P24723	PRKCH	NM_006255.1	1496	0.55857	protein kinase C, eta	2693.21	9931	7306	4004.8	0.73259	0.1720249	0.0013	Hit	B03R09C21
P24723	PRKCH	NM_006255.1	1500	0.55688	protein kinase C, eta	3467.99	9428	6853	3997.42	0.73259	0.1727094	0.0013	Hit	B03R09C22
Q9H7L9	SUDS3	BC093990.1	1506	0.55311	Sin3 histone deacetylase corepressor complex component SDS3	32935.2	9215	5945	3981	0.5907	0.1742465	0.04599	Hit	B47R18C13
P50613	CDK7	NM_001799.2	1514	0.5488	cyclin-dependent kinase 7	83378.2	8918	5014	3962.22	0.66334	0.17603	0.00459	Hit	B30R05C20
O43602	DCX	NM_178151.1	1517	0.54837	doublecortex; lissencephaly, X-linked (doublecortin) (DCX), transcript variant 4	31147.8	14153	10415	3960.35	0.51729	0.1762087	0.07001	Hit	B19R16C14
P50613	CDK7	NM_001799.2	1526	0.54292	cyclin-dependent kinase 7	73641.2	9477	6574	3936.6	0.66334	0.1785072	0.00459	Hit	B30R05C19
P19139	CSNK2A1	PV3248	1528	0.54266	casein kinase 2, alpha 1 polypeptide (CSNK2A1), transcript variant 2	0	10266	7338	3935.44	0.66738	0.1786201	0.01531	Hit	B05R15C18
Q05397	PTK2	NM_005607.1	1532	0.5396	Focal adhesion kinase 1	61658.9	8484	3840	3922.13	0.53561	0.179929	0.06249	Hit	B19R10C06
Q9UQB9	AURKC	NM_003160.1	1536	0.53793	Serine/threonine-protein kinase 13	5792.35	9688	5911	3914.86	0.6563	0.1806504	0.02103	Hit	B47R12C08
Q16816	PHKG1	PV3853	1547	0.53126	Phosphorylase b kinase gamma catalytic chain, skeletal muscle isoform	2787.56	9062	5863	3885.78	0.51274	0.1835787	0.06252	Hit	B15R15C18
P51813	BMX	NM_001721.2	1557	0.52771	BMX non-receptor tyrosine kinase (BMX), transcript variant 2	102908	11936	10042	3870.3	0.66174	0.1851664	0.03832	Hit	B10R10C02
Q96CW6	SLC7A6OS	BC013778.1	1567	0.522	solute carrier family 7, member 6 opposite strand (SLC7A6OS)	16788	10091	6551	3845.4	0.48241	0.1877628	0.04176	Hit	B33R04C20
Q14653	IRF3	NM_001571.2	1577	0.51702	interferon regulatory factor 3 (IRF3)	66886.9	10061	8799	3823.72	0.72297	0.1900691	0.04646	Hit	B21R09C03
A8K4Z8	HNRPA1	NM_002136.1	1584	0.5128	heterogeneous nuclear ribonucleoprotein A1 (HNRNPA1), transcript variant 1	43086.6	9100	5822	3805.31	0.55754	0.1920616	0.05461	Hit	B23R03C17
Q6PKG0	LARP1	BC033856.1	1591	0.51	La ribonucleoprotein domain family, member 1 (LARP1)	47025.8	12032	8889	3793.14	0.5357	0.1933952	0.05129	Hit	B19R17C16
O95456	PSMG1	NM_003720.1	1592	0.50977	Proteasome assembly chaperone 1	33672.9	9565	6545	3792.14	0.52621	0.1935055	0.0558	Hit	B47R08C06
Q9UHJ3	SFMBT1	NM_001005158.1	1606	0.50167	Scm-like with four MBT domains protein 1	15977.8	8549	5146	3756.83	0.46351	0.1974589	0.10478	Hit	B26R19C16
Q9UK59	DBR1	NM_016216.2	1624	0.49339	Lariat debranching enzyme	11545.7	8658	5160	3720.72	0.47854	0.2016287	0.09731	Hit	B42R19C10
P35236	PTPN7	NM_080588.1	1625	0.4928	protein tyrosine phosphatase, non-receptor type 7 (PTPN7), transcript variant 2	75256.8	10145	7647	3718.19	0.61161	0.2019262	0.01395	Hit	B30R08C19
Q8IWW6	ARHGAP12	BC094719.1	1626	0.49265	Rho GTPase-activating protein 12	73327.5	8919	6922	3717.5	0.73763	0.202007	0.00714	Hit	B12R21C01
Q8IWW6	ARHGAP12	BC094719.1	1643	0.48408	Rho GTPase-activating protein 12	54566.7	9272	7677	3680.17	0.73763	0.2064707	0.00714	Hit	B12R21C02
Q5T0L3	C1orf111	BC032957.1	1649	0.48152	chromosome 1 open reading frame 111 (C1orf111)	4359.78	8111	4555	3669	0.4068	0.2078352	0.06256	Hit	B33R12C20
P35236	PTPN7	NM_080588.1	1660	0.47614	protein tyrosine phosphatase, non-receptor type 7 (PTPN7), transcript variant 2	74420.4	9155	5870	3645.58	0.61161	0.2107407	0.01395	Hit	B30R08C20
Q96QK1	VPS35	NM_018206.3	1662	0.47579	vacuolar protein sorting 35 homolog (S. cerevisiae) (VPS35)	29463.3	8516	5021	3644.05	0.55742	0.2109322	0.07097	Hit	B31R09C22
P11532	DMD	NM_004013.1	1681	0.46963	dystrophin (muscular dystrophy, Duchenne and Becker types) (DMD), transcript variant Dp140	36619.2	10974	9547	3617.18	0.61763	0.2143452	0.0205	Hit	B05R07C01
Q9ESI7	DCX	NM_178152.1	1693	0.4643	Neuronal migration protein doublecortin	61941.2	9375	6422	3593.95	0.4628	0.2173632	0.10601	Hit	B34R13C21
Q02221	COX6A2	NM_005205.2	1708	0.45693	cytochrome c oxidase subunit VIa polypeptide 2 (COX6A2), nuclear gene encoding mitochondrial protein	20505.5	8553	4702	3561.84	0.65105	0.221642	0.03275	Hit	B31R17C05
P22612	PRKACG	NM_002732.2	1730	0.45077	protein kinase, cAMP-dependent, catalytic, gamma (PRKACG)	2902.03	8047	4780	3535	0.59686	0.2253143	0.01846	Hit	B15R09C17
Q9NZU5	LMCD1	NM_014583.2	1731	0.45038	LIM and cysteine-rich domains 1 (LMCD1)	85989.5	8559	5880	3533.3	0.45383	0.2255511	0.12326	Hit	B13R17C09
P10721	KIT	P3081	1734	0.44988	v-kit Hardy-Zuckerman 4 feline sarcoma viral oncogene homolog (KIT), transcript variant 1; see catalog number for deta	0	8248	4950	3531.15	0.67258	0.2258493	0.02389	Hit	B09R15C18
Q6PKG0	LARP1	BC033856.1	1735	0.44908	La ribonucleoprotein domain family, member 1 (LARP1)	39851.7	11453	8717	3527.66	0.5357	0.2263352	0.05129	Hit	B19R17C15
A8K4Z8	HNRPA1	NM_002136.1	1742	0.44786	heterogeneous nuclear ribonucleoprotein A1 (HNRNPA1), transcript variant 1	39486.4	8752	5263	3522.34	0.55754	0.2270798	0.05461	Hit	B23R03C18
P11532	DMD	NM_004013.1	1748	0.44591	dystrophin (muscular dystrophy, Duchenne and Becker types) (DMD), transcript variant Dp140	30378.6	11203	9243	3513.83	0.61763	0.2282775	0.0205	Hit	B05R07C02
O95456	PSMG1	NM_003720.1	1753	0.44372	Proteasome assembly chaperone 1	26918.4	8862	5824	3504.27	0.52621	0.2296335	0.0558	Hit	B47R08C05
P51817	PRKX	PV3813	1769	0.4393	protein kinase, X-linked (PRKX)	3735.23	8632	6188	3485.01	0.55576	0.2324039	0.02937	Hit	B15R15C15
Q8NC51	SERBP1	BC020555.1	1782	0.43028	SERPINE1 mRNA binding protein 1 (SERBP1)	75275.2	10794	10002	3445.73	0.44146	0.2382132	0.0823	Hit	B23R14C12
P22612	PRKACG	NM_002732.2	1784	0.42986	protein kinase, cAMP-dependent, catalytic, gamma (PRKACG)	3087.43	7780	4584	3443.89	0.59686	0.2384898	0.01846	Hit	B15R09C18
P10721	KIT	P3081	1807	0.42296	v-kit Hardy-Zuckerman 4 feline sarcoma viral oncogene homolog (KIT), transcript variant 1; see catalog number for deta	0	8218	5400	3413.82	0.67258	0.2430923	0.02389	Hit	B09R15C17
Q02221	COX6A2	NM_005205.2	1820	0.41992	cytochrome c oxidase subunit VIa polypeptide 2 (COX6A2), nuclear gene encoding mitochondrial protein	21601.2	8208	4847	3400.59	0.65105	0.2451604	0.03275	Hit	B31R17C06
O00459	PIK3R2	BC014170.1	1822	0.41975	Phosphatidylinositol 3-kinase regulatory subunit beta	8741.26	8277	5583	3399.85	0.61281	0.2452772	0.04069	Hit	B31R12C22
Q8NEG7	FAM116B	NM_001001794.1	1837	0.41545	family with sequence similarity 116, member B (FAM116B)	22751.4	9336	6678	3381.08	0.44876	0.2482576	0.00666	Hit	B25R09C02
Q8N3C7	CLIP4	NM_024692.3	1842	0.41335	CAP-GLY domain containing linker protein family, member 4 (CLIP4)	43001.6	9749	6586	3371.95	0.4147	0.2497266	0.07825	Hit	B47R08C07
Q8NEG7	FAM116B	NM_001001794.1	1858	0.40817	family with sequence similarity 116, member B (FAM116B)	33132.4	8870	6685	3349.36	0.44876	0.2534194	0.00666	Hit	B25R09C01
P51817	PRKX	PV3813	1865	0.40676	protein kinase, X-linked (PRKX)	5073.55	8749	6010	3343.22	0.55576	0.2544375	0.02937	Hit	B15R15C16
Q96QA6	YPEL2	NM_001005404.3	1881	0.40104	Protein yippee-like 2	35770.9	7971	5443	3318.28	0.46539	0.2586359	0.08262	Hit	B10R18C09
Q8TDX7	NEK7	NM_133494.1	1898	0.39296	NIMA (never in mitosis gene a)- related kinase 7 (NEK7)	53126.5	9288	8252	3283.08	0.70569	0.2647405	0.00718	Hit	B12R18C10
Q8TDX7	NEK7	NM_133494.1	1917	0.38535	NIMA (never in mitosis gene a)- related kinase 7 (NEK7)	63509	9034	8153	3249.9	0.70569	0.2706935	0.00718	Hit	B12R18C09
Q13873	BMPR2	NM_001204.3	1923	0.38353	Bone morphogenetic protein receptor type-2	55365.3	8463	7084	3241.99	0.40952	0.2721424	0.03304	Hit	B33R10C02
O14531	DPYSL4	NM_006426.1	1938	0.37792	dihydropyrimidinase-like 4 (DPYSL4)	50103.4	8847	6097	3217.55	0.42803	0.2766943	0.05625	Hit	B35R07C17
O00459	PIK3R2	BC014170.1	1947	0.37612	Phosphatidylinositol 3-kinase regulatory subunit beta	11340.7	8357	5697	3209.68	0.61281	0.2781847	0.04069	Hit	B31R12C21
Q96L93	C20orf23	BC034984.1	1966	0.37023	Kinesin-like protein KIF16B	4063.18	8288	5667	3184.04	0.80842	0.2831234	0.00686	Hit	B21R06C02
Q5VT25	CDC42BPA	NM_003607.1	1970	0.36825	Serine/threonine-protein kinase MRCK alpha	55944.1	8093	5514	3175.38	0.41581	0.284821	0.11167	Hit	B34R09C17
Q96L93	C20orf23	BC034984.1	1982	0.36318	Kinesin-like protein KIF16B	4738.37	8048	6412	3153.31	0.80842	0.2892186	0.00686	Hit	B21R06C01
Q96EY5	FAM125A	NM_138401.2	1993	0.35896	family with sequence similarity 125, member A (FAM125A)	22997.7	8182	5818	3134.92	0.6575	0.2929594	0.02011	Hit	B31R19C17
Q96G95	BCKDK	BC009872.1	2032	0.34979	branched chain ketoacid dehydrogenase kinase (BCKDK)	19729.9	8282	5982	3094.96	0.44817	0.3013434	0.05461	Hit	B05R02C11

Q13873	BMPR2	NM_001204.3	2033	0.34956	Bone morphogenetic protein receptor type-2	51813.4	7750	6195	3093.96	0.40952	0.3015579	0.03304	Hit	B33R10C01
Q9ESI7	DCX	NM_178152.1	2036	0.34928	Neuronal migration protein doublecortin	65787.3	8520	5266	3092.73	0.4628	0.3018225	0.10601	Hit	B34R13C22
Q8N302	AGGF1	BC032844.1	2037	0.34766	Angiogenic factor with G patch and FHA domains 1	10517.2	9259	6270	3085.65	0.47451	0.3033484	0.05466	Hit	B27R18C12
Q9NX09	DDIT4	NM_019058.1	2048	0.34376	DNA-damage-inducible transcript 4 protein	57212.4	10106	6586	3068.66	0.4994	0.3070599	0.08188	Hit	B37R16C16
Q8NC51	SERBP1	BC020555.1	2049	0.34331	SERPINE1 mRNA binding protein 1 (SERBP1)	54088.6	9168	8133	3066.72	0.44146	0.3074892	0.0823	Hit	B23R14C11
Q14088	RAB33A	NM_004794.1	2053	0.34218	RAB33A, member RAS oncogene family (RAB33A)	120808	14376	22951	3061.8	0.62369	0.3085784	0.02669	Hit	B12R14C22
Q96EY5	FAM125A	NM_138401.2	2065	0.33879	family with sequence similarity 125, member A (FAM125A)	19853.6	8149	5340	3047	0.6575	0.3118917	0.02011	Hit	B31R19C18
Q5MJ09	SPANXN3	NM_001009609.1	2080	0.33415	SPANX family, member N3 (SPANXN3)	13177.2	8595	6578	3026.77	0.44754	0.3165067	0.09785	Hit	B34R07C16
Q8N3C7	CLIP4	NM_024692.3	2085	0.33222	CAP-GLY domain containing linker protein family, member 4 (CLIP4)	56130	9258	6468	3018.39	0.4147	0.3184504	0.07825	Hit	B47R08C08
P48047	ATP5O	NM_001697.1	2098	0.32921	ATP synthase, H+ transporting, mitochondrial F1 complex, O subunit (oligomycin sensitivity conferring protein) (ATP5	17894.2	8718	5577	3005.28	0.54437	0.3215229	0.02568	Hit	B47R16C07
O14531	DPYSL4	NM_006426.1	2124	0.32143	dihydropyrimidinase-like 4 (DPYSL4)	53222.1	8564	6185	2971.37	0.42803	0.3296856	0.05625	Hit	B35R07C18
Q9NZU5	LMCD1	NM_014583.2	2130	0.32038	LIM and cysteine-rich domains 1 (LMCD1)	18284.5	7633	5471	2966.78	0.45383	0.3308131	0.12326	Hit	B13R17C10
Q96QA6	YPEL2	NM_001005404.3	2144	0.31698	Protein yippee-like 2	38224	7517	5002	2951.97	0.46539	0.3344956	0.08262	Hit	B10R18C10
Q14088	RAB33A	NM_004794.1	2148	0.31615	RAB33A, member RAS oncogene family (RAB33A)	86889.1	15862	23196	2948.36	0.62369	0.335401	0.02669	Hit	B12R14C21
O43865	AHCYL1	NM_006621.3	2152	0.31471	S-adenosylhomocysteine hydrolase-like 1 (AHCYL1)	64084.7	9012	5880	2942.1	0.53316	0.3369837	0.05186	Hit	B31R08C20
P35544	FAU	NM_001997.2	2158	0.31217	Finkel-Biskis-Reilly murine sarcoma virus (FBR-MuSV) ubiquitously expressed (FAU)	18528.5	7519	4968	2930.99	0.42852	0.3398156	0.10115	Hit	B34R12C11
Q9Y6V7	DDX49	NM_019070.1	2172	0.3083	DEAD (Asp-Glu-Ala-Asp) box polypeptide 49 (DDX49)	102935	7366	5002	2914.15	0.66403	0.3441793	0.02719	Hit	B37R03C01
P48047	ATP5O	NM_001697.1	2184	0.30461	ATP synthase, H+ transporting, mitochondrial F1 complex, O subunit (oligomycin sensitivity conferring protein) (ATP5	18700.2	8364	5248	2898.07	0.54437	0.348424	0.02568	Hit	B47R16C08
Q96G95	BCKDK	BC009872.1	2211	0.29699	branched chain ketoacid dehydrogenase kinase (BCKDK)	16872.4	7861	5760	2864.84	0.44817	0.3574501	0.05461	Hit	B05R02C12
P29083	GTF2E1	NM_005513.1	2216	0.2959	general transcription factor IIE, polypeptide 1, alpha 56kDa (GTF2E1)	12051.1	8393	6165	2860.09	0.44468	0.3587707	0.05002	Hit	B47R08C21
Q8N302	AGGF1	BC032844.1	2225	0.29496	Angiogenic factor with G patch and FHA domains 1	11569.1	8884	7033	2855.99	0.47451	0.3599123	0.05466	Hit	B27R18C11
O43852	CALU	NM_001219.2	2249	0.28992	calumenin (CALU)	64935.6	8769	6097	2834.04	0.51243	0.3661325	0.01495	Hit	B35R03C20
Q969D9	TSLP	NM_138551.1	2261	0.28629	thymic stromal lymphopoietin (TSLP), transcript variant 2	19521.9	8204	6280	2818.2	0.57729	0.3707213	0.00478	Hit	B05R05C06
Q96BT0	PHF20L1	NM_198513.1	2269	0.2845	PHD finger protein 20-like 1 (PHF20L1), transcript variant 3	74683.8	7851	5138	2810.43	0.73704	0.3730064	0.00102	Hit	B34R02C21
Q96BT0	PHF20L1	NM_198513.1	2272	0.28357	PHD finger protein 20-like 1 (PHF20L1), transcript variant 3	73146	7632	5358	2806.36	0.73704	0.3742085	0.00102	Hit	B34R02C22
Q9Y6V7	DDX49	NM_019070.1	2274	0.28308	DEAD (Asp-Glu-Ala-Asp) box polypeptide 49 (DDX49)	83199.9	7400	5148	2804.23	0.66403	0.3748427	0.02719	Hit	B37R03C02
Q969D9	TSLP	NM_138551.1	2276	0.28193	thymic stromal lymphopoietin (TSLP), transcript variant 2	19820.5	7971	7165	2799.21	0.57729	0.3763382	0.00478	Hit	B05R05C05
Q8WTQ4	C16orf78	NM_144602.1	2284	0.28018	chromosome 16 open reading frame 78 (C16orf78)	15042.2	7801	5929	2791.58	0.51464	0.3786303	0.01479	Hit	B15R02C15
O43852	CALU	NM_001219.2	2303	0.27631	calumenin (CALU)	49455.5	8919	7225	2774.73	0.51243	0.3837685	0.01495	Hit	B35R03C19
Q9UMR2	DDX19B	NM_007242.3	2309	0.27465	DEAD (Asp-Glu-Ala-As) box polypeptide 19B (DDX19B), transcript variant 1	71869.6	7415	5377	2767.49	0.40435	0.3860074	0.04015	Hit	B30R09C06
O43865	AHCYL1	NM_006621.3	2339	0.26695	S-adenosylhomocysteine hydrolase-like 1 (AHCYL1)	59923.9	8853	6752	2733.94	0.53316	0.3966482	0.05186	Hit	B31R08C19
Q8WTQ4	C16orf78	NM_144602.1	2340	0.26692	chromosome 16 open reading frame 78 (C16orf78)	0	7854	5340	2733.8	0.51464	0.3966942	0.01479	Hit	B15R02C16
Q9NX09	DDIT4	NM_019058.1	2341	0.26668	DNA-damage-inducible transcript 4 protein	59086.7	9135	6051	2732.77	0.4994	0.3970286	0.08188	Hit	B37R16C15
Q8TBG7	RPIB9	BC022520.1	2347	0.26508	RUN domain containing 3B (RPIB9)	49155	7927	6561	2725.78	0.63605	0.3993034	0.01341	Hit	B31R02C17
Q5VT25	CDC42BPA	NM_003607.1	2361	0.26159	Serine/threonine-protein kinase MRCK alpha	62141.3	7518	4606	2710.59	0.41581	0.4043131	0.11167	Hit	B34R09C18
Q8TCT6	UNQ1887	BC025781.1	2377	0.25882	signal peptide peptidase 3 (UNQ1887)	2794.79	11682	10494	2698.51	0.47848	0.408368	0.02618	Hit	B39R17C19
A4D1E9	GTPBP10	BC021573.1	2399	0.25475	GTP-binding protein 10	35852.8	7983	6075	2680.79	0.6374	0.4144267	0.01133	Hit	B31R09C11
O60488	ACSL4	NM_022977.1	2403	0.25342	acyl-CoA synthetase long-chain family member 4 (ACSL4), transcript variant 2	22016	8540	7597	2674.96	0.60308	0.4164486	0.00441	Hit	B23R08C08
Q8TBG7	RPIB9	BC022520.1	2404	0.25332	RUN domain containing 3B (RPIB9)	23957.8	8286	6692	2674.56	0.63605	0.4165882	0.01341	Hit	B31R02C18
P29083	GTF2E1	NM_005513.1	2414	0.25106	general transcription factor IIE, polypeptide 1, alpha 56kDa (GTF2E1)	67689.9	7840	6375	2664.68	0.44468	0.4200504	0.05002	Hit	B47R08C22
